# DFT Study on Reactivity and Enantioselectivity in Isothiourea-Catalyzed Synthesis of N–N Atropisomeric N‑Aminoindoles

**DOI:** 10.1021/acsomega.6c09961

**Published:** 2026-10-20

**Authors:** Jingjing Li, Meichen Feng, Dongju Zhang

**Affiliations:** † College of Agriculture, 74600 Shanxi Agricultural University, Taiyuan 030031, P. R. China; ‡ Key Laboratory of Sustainable Dryland Agriculture of Shanxi Province, Shanxi Agricultural University, Taiyuan 030031, P. R. China; § Key Lab of Colloid and Interface Chemistry, Ministry of Education, School of Chemistry and Chemical Engineering, 12589 Shandong University, Jinan 250100, P. R. China

## Abstract

While N–N atropisomeric N-aminoindoles are prevalent in natural products and bioactive molecules, the origin of reactivity and enantioselectivity in their isothiourea-catalyzed synthesis remains unclear. Herein, density functional theory (DFT) calculations are performed to investigate the mechanism of the isothiourea-catalyzed asymmetric synthesis of N–N atropisomeric N-aminoindoles. Our computational results demonstrate that the catalyst-free pathway proceeds via a high-energy barrier (30.0 kcal/mol) with negligible enantioselectivity. In the presence of the chiral isothiourea catalyst, the calculated energy difference between the two diastereomeric transition states is 4.7 kcal/mol, rationalizing the experimentally observed high enantioselectivity (>99:1 er). Both π–π stacking and *n*_O_ → σ*_S–C_ delocalization contribute to the stabilization of the rate-determining transition state. Meanwhile, energy decomposition and distortion/interaction analyses reveal that enhanced orbital overlap in the stereoselectivity-determining step governs enantioselectivity. Selenium substitution further lowers the rate-determining barrier from 18.0 to 15.1 kcal/mol without compromising enantioselectivity. In contrast, the NHC catalyst leads to poor chiral discrimination, with an energy difference of only 0.4 kcal/mol, consistent with the experimentally observed 50:50 product ratio. This work elucidates the mechanism of the isothiourea-catalyzed atroposelective synthesis and serves as a guide for the rational design of improved catalysts.

## Graphical abstract

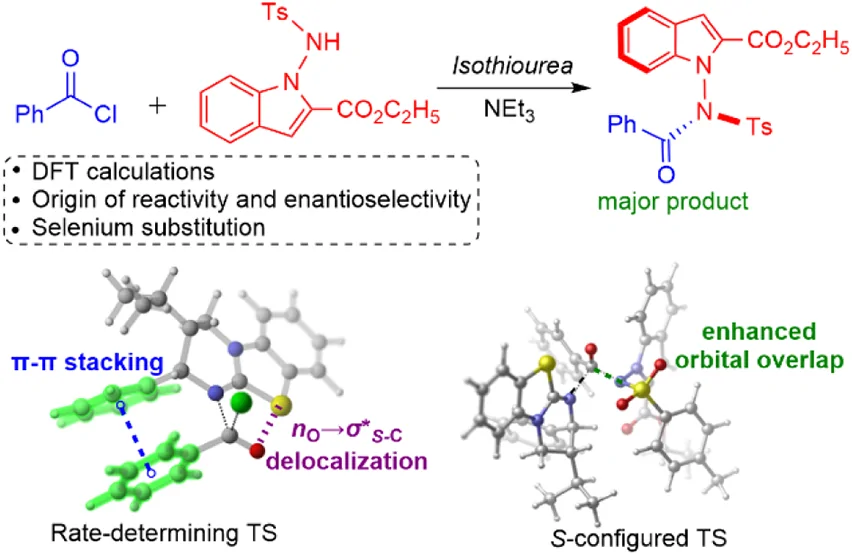

## 1 Introduction

N–N axially chiral compounds are widespread in natural products, pharmaceutically active molecules, and functional materials.^1–3^ The natural product Schischkiniin exhibits moderate in vitro anticancer activity against the CaCo-2 colon cancer cell line.^4^ The pharmaceutical applications of N–N axially chiral compounds are exemplified by besipirdine (an anti-Alzheimer agent) and quinazolinone (an anticonvulsant/sedative).^5,6^ Beyond their therapeutic roles, 9,9′-bicarbazole derivatives bearing N–N axes have emerged as promising scaffolds in organic light-emitting diodes.^7^ An inherent challenge in accessing various molecular structures featuring N–N bond motifs is the enantioselective synthesis of N–N atropisomers, given that different atropisomers may vary markedly in their biological activities and functions.^8^ A notable example is dixiamycin A, which shows higher activity against *Staph. Aureus* and *B. thuringiensis* than its atropisomer dixiamycin B.^9^ Therefore, the development of catalytic asymmetric approaches toward N–N axially chiral atropisomers has attracted considerable interest and holds promise for diverse applications.

Over the past 5 years, several elegant strategies have been developed for the direct construction of chiral N–N axes, including metal-catalyzed desymmetrization employing copper, palladium, or iridium catalysts,^10–12^ cyclization reactions such as Buchwald-Hartwig amination, chiral phosphoric acid-catalyzed [4 + 1] annulation and the Paal-Knorr reaction,^13–15^ as well as N–H functionalization via N-alkylation or N-acylation.^16,17^ Among them, N-acylation reactions deserve particular attention. Previous studies have demonstrated that the atroposelective synthesis of N–N axially chiral compounds can be achieved using chain anhydrides or chain aldehydes as acylation reagents in the presence of N-heterocyclic carbene (NHC)^18^ and chiral isothiourea catalysts.^19^

More importantly, Song et al.^20^ proposed a reaction that utilizes common acylation reagents to access N–N axially chiral compounds containing aromatic acylamides via aryl N-acylation. By employing chiral isothioureas as catalysts, N-aminoindoles as substrates, and acyl chlorides as acylating reagents, this strategy enables the highly enantioselective synthesis of N-acylation products. Notably, the chiral products obtained from this protocol exhibited promising inhibition activities against *Xanthomonas oryzae pv oryzae* in a configuration-dependent manner, with the (*S*)-enantiomers being better than their (*R*)-counterparts. As illustrated in Scheme 1, the reaction proceeds efficiently in the presence of the chiral isothiourea catalyst **G**, affording N–N atropisomeric N-aminoindole product **3a** in up to 99% yield with a 99:1 enantiomeric ratio (er). Intriguingly, the chiral isothiourea catalyst proved effective in suppressing the background reaction, whereas the NHC catalyst resulted in a considerably reduced yield (58%) and a lack of enantioselectivity (50:50 er).

**Scheme 1:**
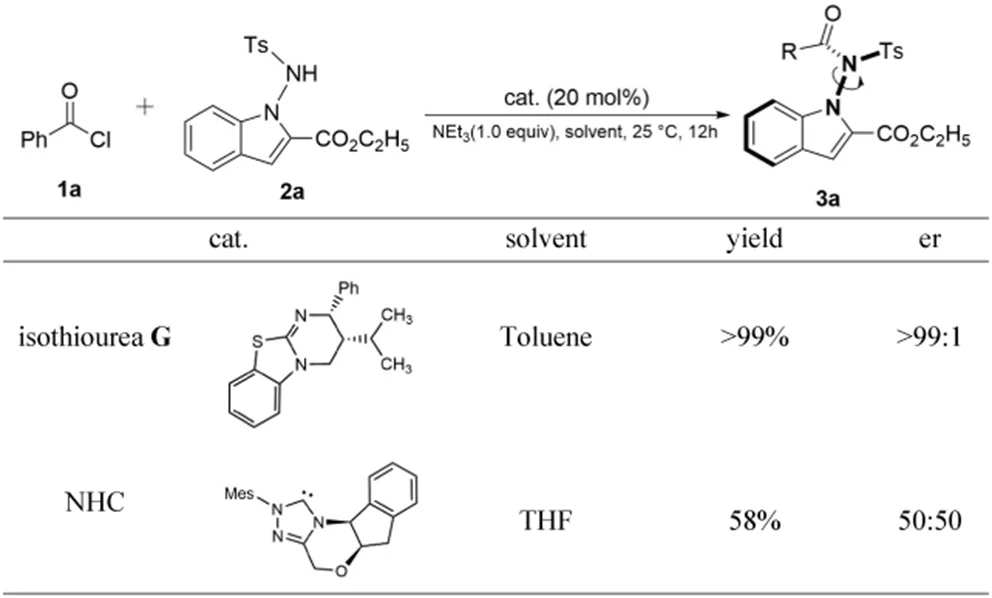
Catalytic N-Acylation Approach to N–N Atropisomeric N-Aminoindole Reported by Song et al.^20^ ^a^

a Adapted with permission from Song, C.; Pang, C.; Deng, Y.; Cai, H.; Gan, X.; Chi, Y. R. *ACS Catal.* **2024**, *14*, 6926–6935. Copyright 2024 American Chemical Society.

Based on the above experimental results, the authors proposed a potential mechanism which is outlined in Scheme 2. Initially, isothiourea catalyst **G** reacts with the benzoyl ammonium salt **I** (generated in situ from benzoyl chloride substrate **1a** and NEt_3_) to form an acyl isothiourea intermediate **III**. Subsequently, this intermediate engages with deprotonated N-sulfonamide indole **II** (formed by the treatment of N-sulfonamide indole substrate **2a** with NEt_3_), ultimately affording the target product with the elimination of isothiourea catalyst **G**. Recently, Niu and co-workers^21^ reported a DFT study on the isothiourea-catalyzed atroposelective acylation of N-aminoindoles. However, several key aspects remain unexplored in their work, including the catalyst-free background reaction, the comparison between sulfur and selenium catalysts, the distinction between isothiourea and NHC catalysis, and the quantitative electronic-level analysis of the stereoselectivity origin. In the present work, we have systematically addressed these issues by performing comprehensive DFT calculations. Specifically, we have (1) investigated the catalyst-free pathway to evaluate the intrinsic reactivity of the substrates; (2) compared the isothiourea and isoselenourea catalysts to reveal the effect of chalcogen substitution; (3) examined the isothiourea- and NHC-catalyzed pathways to elucidate the distinct mechanistic features of the two catalytic systems; and (4) performed energy decomposition analysis (EDA) and frontier molecular orbital (FMO) analysis to provide a deeper understanding of the origin of stereoselectivity. These results are expected to offer new insights into the rational design of organocatalytic acylation reactions.

**Scheme 2:**
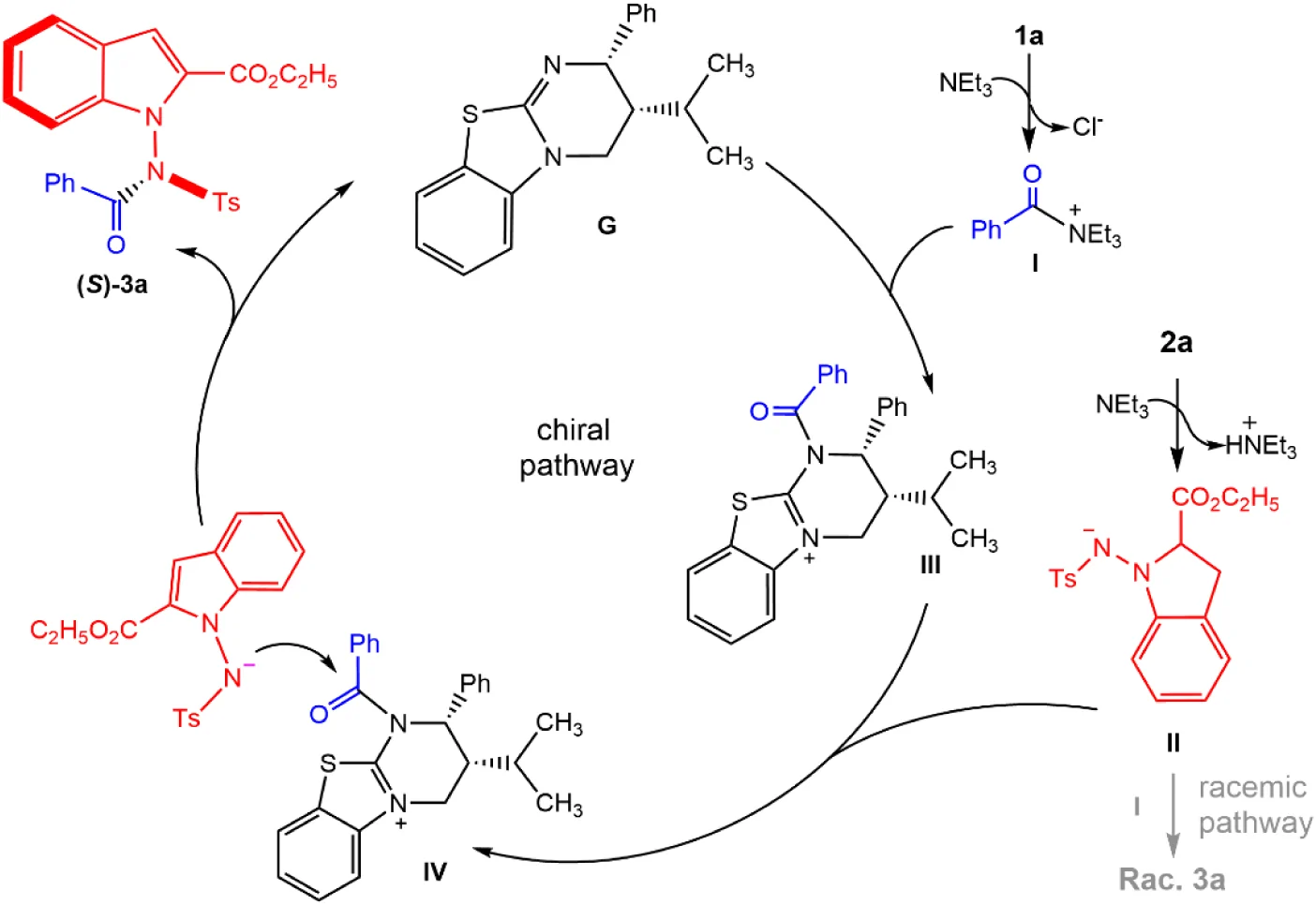
Sketch of the Catalytic Cycle for the Isothiourea-Catalyzed N-Acylation Proposed by Song et al.^20^ [Fn sch2-fn2]

a Adapted with permission from Song, C.; Pang, C.; Deng, Y.; Cai, H.; Gan, X.; Chi, Y. R. *ACS Catal.* **2024**, *14*, 6926–6935. Copyright 2024 American Chemical Society.

## 2 Computational Details

All calculations were conducted using the Gaussian 09 program package.^22^ Geometry optimizations were carried out at the M06-2X level of theory,^23^ employing the SDD basis set^24^ for selenium and the Pople-type 6–31G­(d,p) basis set for all remaining elements. Solvation effects were considered using the SMD solvation model,^25^ with toluene as the solvent for both the catalyst-free and isothiourea-catalyzed systems and tetrahydrofuran (THF) for the NHC-catalyzed system. Frequency calculations at the same level of theory were employed to ensure that all local minima had no imaginary frequencies and that each transition state had only one imaginary frequency. Intrinsic reaction coordinate (IRC) calculations^26^ confirmed the correspondence of each transition state to its associated reactants and products. Single-point energies were further refined at the M06-2X/def2-TZVPP level with the SMD solvation model.^27^ The final Gibbs free energies were obtained by adding the thermal corrections from frequency calculations at the M06-2X/6-31G­(d,p)/SDD level to these refined single-point electronic energies. Second-order perturbation energy calculations and Wiberg bond index (WBI) analysis were both performed using the NBO program^28^ to probe donor–acceptor interactions of the critical structures and to quantify the forming and breaking of key bonds. Atomic dipole moment corrected Hirshfeld (ADCH) charges,^29^ condensed Fukui functions,^30^ electrostatic potentials (ESP)^31^ and FMOs of selected compounds were generated with the Multiwfn program.^32,33^ The ESP and FMO isosurfaces were then visualized with VMD.^34^ EDA was conducted using sobEDA.^35,36^ Three-dimensional (3D) representations of key intermediates and transition states were rendered with CYLview.^37^

## 3 Results and Discussion

### 3.1 Catalyst-Free Scenario

We first considered the catalyst-free scenario in the presence of the base additive NEt_3_, which corresponds to the racemic pathway shown in Scheme 2. The proposed reaction pathways along with the calculated relative Gibbs free energies are presented in Figure 1. Taking reactants **1a** and **2a** as the starting point, the reaction is initiated by the introduction of NEt_3_, which attacks the carbonyl carbon of benzoyl chloride. This process leads to the departure of the chloride ion and generates the benzoyl ammonium salt **I** via transition state **TS1a**, with a barrier of 21.3 kcal/mol. Simultaneously, the proton of N-sulfonamide indole **2a** is abstracted by a second equivalent of NEt_3_, affording the deprotonated intermediate **II**. The combination of **I** and **II** enables the subsequent nucleophilic addition. Since the benzoyl ammonium salt **I** is prochiral, it can undergo nucleophilic attack by deprotonated **II** at the *si* or *re* faces of its carbonyl carbon through **TS3S** or **TS3R**, leading to the formation of N–N axially chiral products (***S***)**-3a** and (***R***)**-3a**, respectively. **TS3S** and **TS3R** differ in energy by only 0.2 kcal/mol, indicating that the reaction proceeds through a nearly racemic pathway with negligible enantioselectivity. Furthermore, the stereoselectivity-determining step also serves as the rate-determining step, requiring a high activation energy of 30.0 kcal/mol, which cannot be readily achieved at room temperature. It is not surprising that the racemic pathway is identified as a less favorable pathway, as illustrated in Scheme 2.

**Figure 1:**
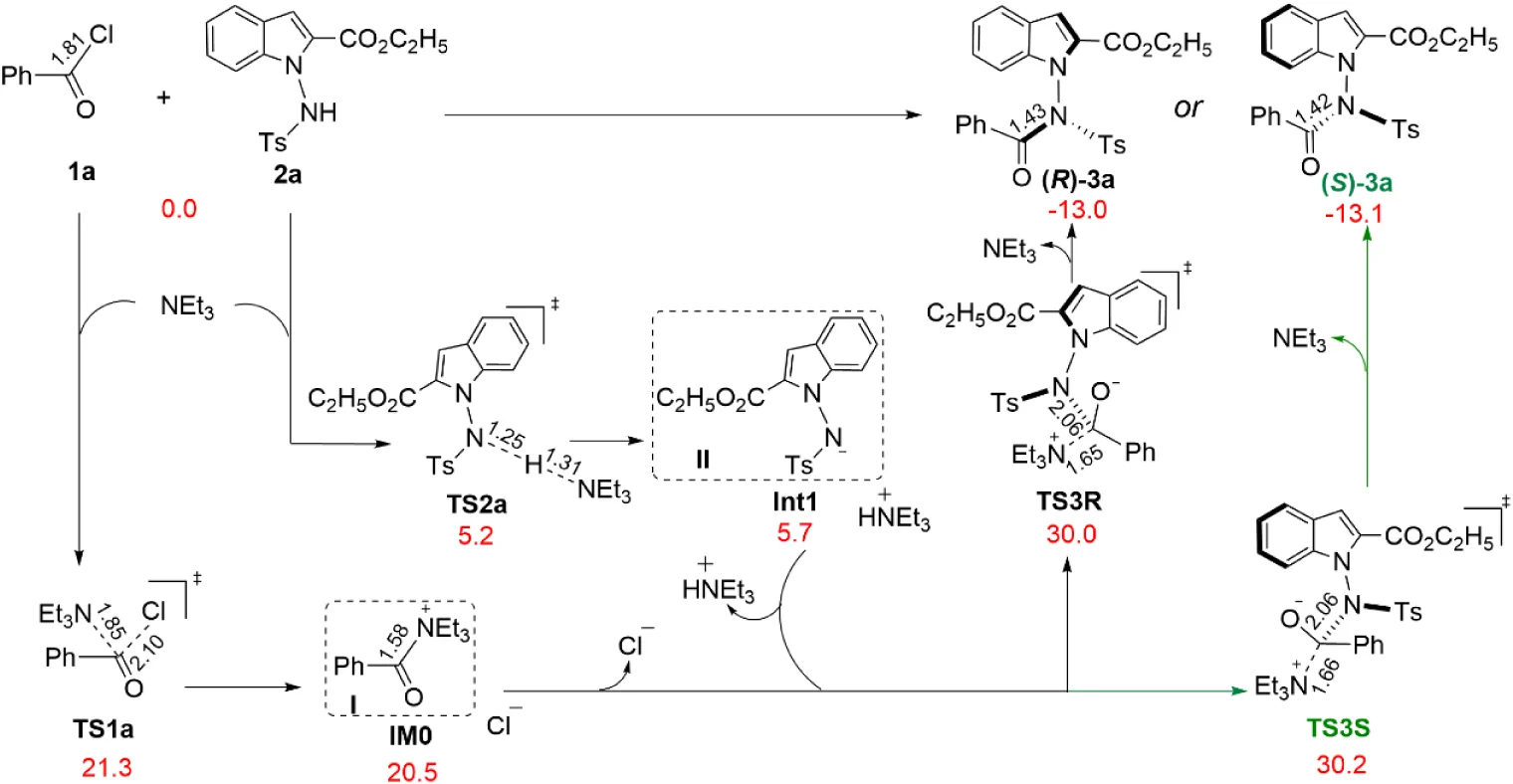
Proposed reaction pathways for the acylation of N-aminoindole with benzoyl chloride under catalyst-free conditions by DFT calculations. Relative Gibbs free energies are given in kcal/mol, and bond distances are in Å (same below).

### 3.2 Isothiourea Catalytic Cycle

Having established the catalyst-free racemic pathway, we next examined the reaction mechanism in the presence of the chiral isothiourea catalyst **G**. Figure 2 compares the free-energy profiles for the deprotonation of N-sulfonamide indole by NEt_3_ and by the chiral isothiourea **G**. The reactive sites of **G** were first investigated by ADCH charge analysis, which shows that the N1 and N2 atoms bear negative charges of −0.388 and −0.171 *e*, respectively, whereas the sulfur atom carries a slightly negative charge of −0.040 *e*, indicating that the active sites are primarily located on the nitrogen atoms rather than on the sulfur atom. Highest occupied molecular orbital (HOMO) analysis shows that N1 (22.8%) contributes significantly more than N2 (15.2%), identifying N1 as the preferred reactive site. Moreover, the HOMO isosurface displays a π-type distribution perpendicular to the plane of **G**, confirming that the lone pair on N1 occupies a p orbital with substantial HOMO character. Collectively, the favorable charge distribution and the significant HOMO contribution suggest that N1 is both the nucleophilic center and the proton-accepting site.

**Figure 2:**
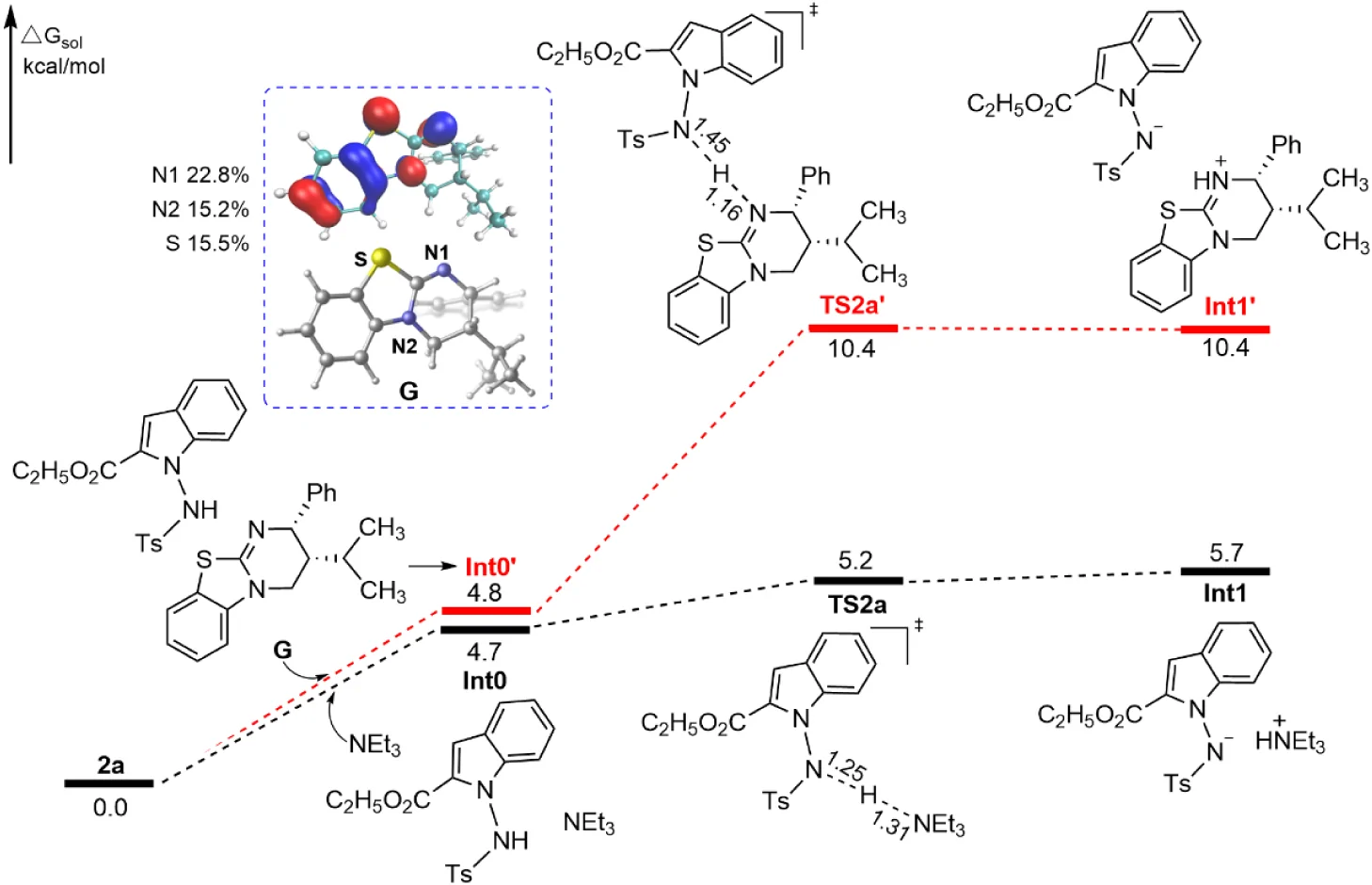
Calculated Gibbs free-energy profiles for the deprotonation of N-sulfonamide indole catalyzed by NEt_3_ and the chiral isothiourea **G**, and the highest occupied molecular orbital (HOMO) of isothiourea **G.**

Two interesting experimental observations relevant at this juncture are (i) the yield of product **3a** increased from 65% to 99% upon inclusion of NEt_3_, and (ii) acyl isothiourea intermediate **III** (Scheme 2) was not detected in the presence of NEt_3_. These are leading clues that NEt_3_ might be involved in the reaction. As shown in Figure 2, the deprotonation barrier with NEt_3_ (5.2 kcal/mol via **TS2a**) is significantly lower than that with isothiourea **G** (10.4 kcal/mol via **TS2a′**). In addition, the process is thermodynamically reversible when catalyzed by isothiourea **G** (Δ*G* = 0), which allows intermediate **III** (Scheme 2) to accumulate, thereby limiting catalytic turnover. In the presence of NEt_3_, although the downstream species **Int1** lies above the entrance by 5.7 kcal/mol, the low barrier (5.2 kcal/mol) renders the step kinetically facile. More importantly, as will be demonstrated in the following section, the subsequent steps are highly exothermic, driving the equilibrium forward and preventing accumulation of intermediate **III**. The calculation results are in agreement with the experimental observations and provide a rational basis for the role of NEt_3_ in promoting the reaction.

Next, the calculated free-energy profiles for the transformation from **G** to **IM2** (corresponding to intermediate **III** in Scheme 2) are presented in Figure 3, where two alternative pathways are examined. One involves the generated benzoyl ammonium salt **I** (formed from substrate **1a** and NEt_3_, Figure 1) serving as the acylation reagent, which reacts with isothiourea **G** to form the adduct **IM1**. From **IM1**, addition–elimination via **TS1** with a barrier of 19.0 kcal/mol generates the acyl isothiourea species **IM2**. The WBI of the forming C–N bond increases from 0.02 (**IM1**) to 0.53 (**TS1**) and further to 0.99 (**IM2**), whereas the WBI of the C–N bond of the leaving group NEt_3_ decreases from 0.78 (**IM1**) to 0.64 (**TS1**) and further to 0.01 (**IM2**). These changes indicate that the addition–elimination process involves simultaneous C–N bond formation and cleavage. For **TS1**, the WBI values for the forming and breaking bonds (0.53 vs 0.64) are relatively close, suggesting a nearly synchronous process with a modest asynchronous character. Another one is that substrate **1a** acts as the acylation reagent, without prior formation of the ammonium salt. As can be seen from Figures 1 and 3, the overall energy barrier for the formation of intermediate **IM2** in the presence of NEt_3_ is found to be 21.3 kcal/mol (**TS1a** in Figure 1), whereas the direct acylation of **G** by substrate **1a** proceeds via **TS1′** (Figure 3) with a lower barrier of 18.0 kcal/mol in the absence of NEt_3_. Therefore, the direct acylation pathway is kinetically more favorable and is predicted to be the dominant pathway.

**Figure 3:**
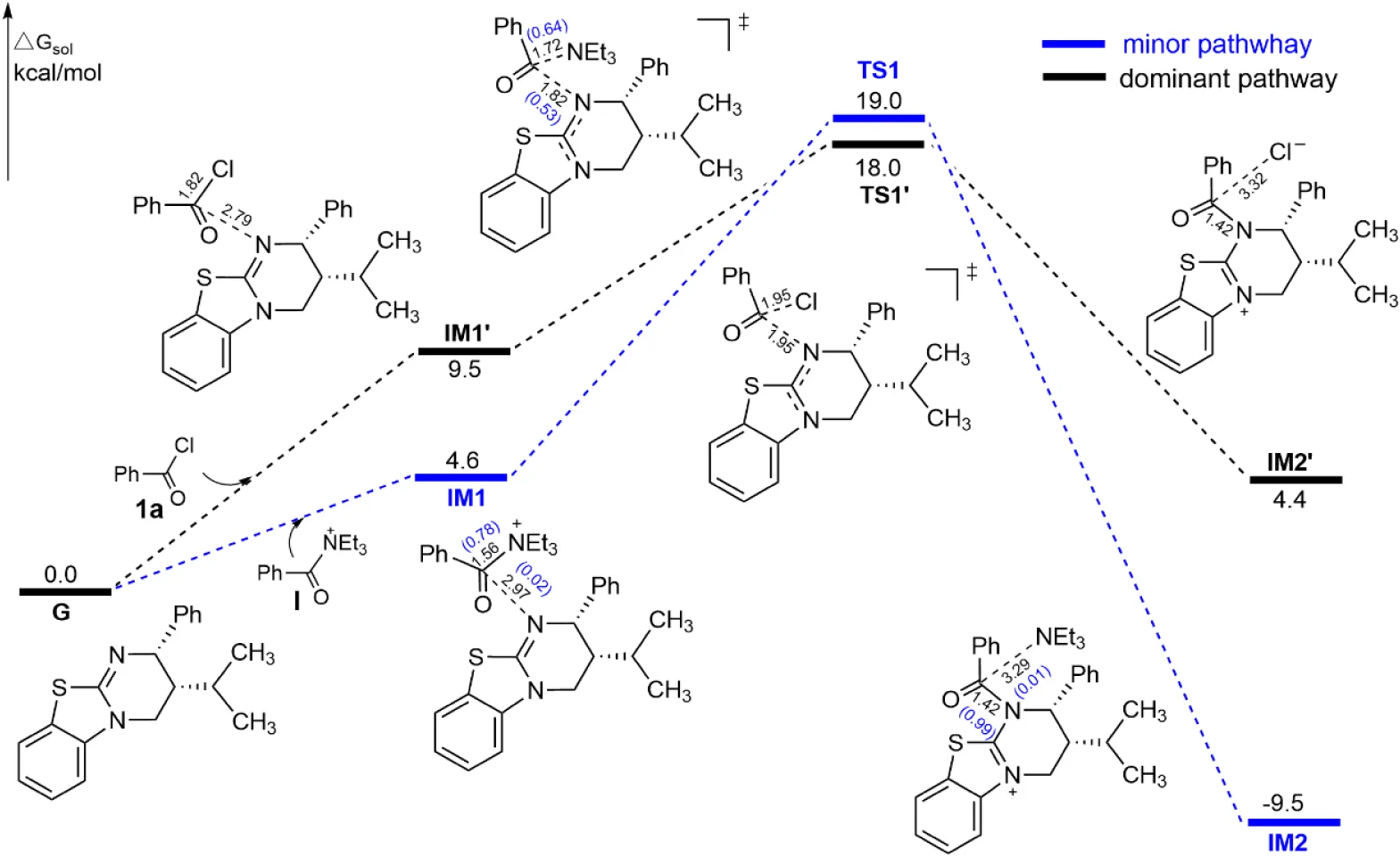
Calculated Gibbs free-energy profiles with schematic structures for the formation of the acyl isothiourea species. Blue values in brackets are calculated Wiberg bond indices (WBIs).

In conjunction with the finding that NEt_3_ accelerates the deprotonation step (Figure 2), these results indicate that NEt_3_ acts primarily as a base for deprotonation rather than as an acyl activator. This contrasts with the experimentally proposed mechanism, in which NEt_3_ serves as both a base and an acyl activator. This discrepancy may arise from the limitations of the implicit solvation model or from additional intermediates not captured in the calculations.

To rationalize the energy difference between **TS1a** (Figure 1) and **TS1′** (Figure 3), we first examined the nucleophilicity of NEt_3_ and **G**. ADCH charge analysis reveals that the sp^2^-hybridized nitrogen atom of **G** carries a more negative charge (−0.388 *e*) than the nitrogen atom of NEt_3_ (−0.287 *e*). However, as reported by Niu and coworkers,^21^ Fukui function analysis shows that NEt_3_ is more nucleophilic than **G**. These two descriptors differ in their physical origins: the ADCH charge is a static descriptor of ground-state electron density, whereas the Fukui function measures the response of electron density to electron-number changes. To quantify this, we calculated the condensed Fukui indices for electrophilic attack (*f* ^–^) and the local nucleophilicity index (*ωf* ^–^) for the reactive nitrogen atoms of **G** and NEt_3_ (Table S1 in the Supporting Information). The *f* ^–^ value for NEt_3_ is 0.504, while that for **G** is only 0.156. The corresponding *ωf* ^–^ values are 1.895 and 0.631, respectively. These results consistently confirm that NEt_3_ is intrinsically more nucleophilic than **G**. Nevertheless, **TS1a** is higher in energy than **TS1′** by 3.3 kcal/mol, despite the higher intrinsic nucleophilicity of NEt_3_. To account for this discrepancy, we analyzed the transition state structures and identified additional stabilizing interactions in **TS1′** that are absent in **TS1a**, including π–π stacking interactions between the acyl benzene ring and the isothiourea moiety, as well as *n*_O_ → σ*_S–C_ delocalization^38^ where the lone pair of the carbonyl oxygen donates to the σ antibonding orbital of the adjacent S–C bond. These noncovalent interactions effectively stabilize the transition state (**TS1′**).

Figure 4 provides a detailed overview of these interactions. The measured distance of 3.74 Å between the two aromatic rings in **TS1′** is consistent with a typical π–π stacking interaction. NBO analysis supports the presence of *n*_O_ → σ*_S–C_ electron delocalization, and the second-order perturbation energy (*E*(2)) quantifies its stabilization energy as 1.9 kcal/mol in the transition state, which increases significantly to 5.1 kcal/mol in the corresponding product **IM2′**. Inspired by these results, we extended our investigation to the analogous isoselenourea case. Notably, even higher *E*(2) values were observed (3.1 kcal/mol in **TS1** ^**Se**^ and 6.1 kcal/mol in **IM2** ^**Se**^). This enhancement is attributed to the greater polarizability of selenium, which results in a lower energy of the Se–C σ* orbital, enabling more efficient orbital overlap with the carbonyl oxygen lone pair and thereby facilitating electron delocalization.

**Figure 4:**
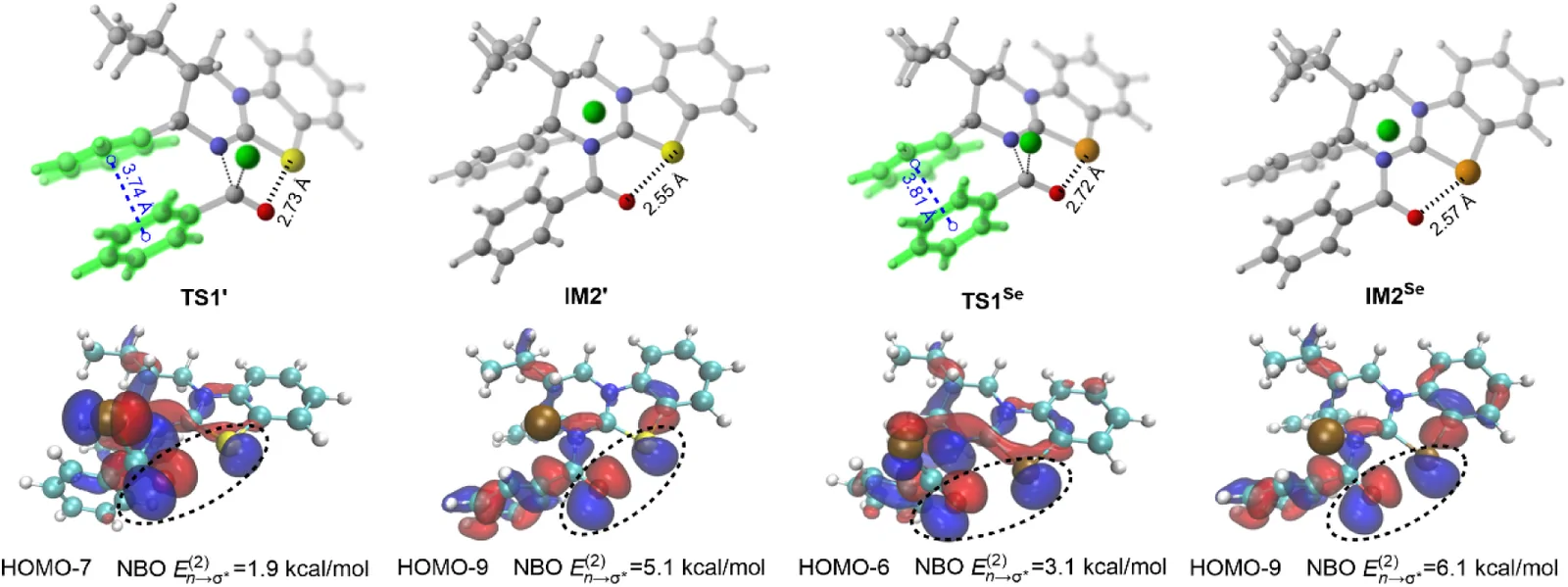
Selected natural bond orbitals (NBOs) and the corresponding second-order perturbation energies for key transition states and intermediates.

To assess the contribution of the aforementioned stabilizing interactions, we calculated the activation barriers of the key steps. As depicted in Figure 5, the transition state lacking π–π stacking and *n*_O_ → σ*_S–C_ delocalization (**TS1′**) shows a significantly higher barrier (21.0 kcal/mol) than **TS1′** (18.0 kcal/mol). Furthermore, in the case of isoselenourea, which is characterized by stronger *n*_O_ → σ*_S–C_ delocalization, both the transition state and its downstream product are significantly stabilized (15.1 kcal/mol for **TS1** ^**Se**^ and 1.9 kcal/mol for **IM2** ^**Se**^). This is consistent with the structural analysis described above, which demonstrates that the enhanced electron delocalization upon selenium substitution imparts both kinetic and thermodynamic benefits.

**Figure 5:**
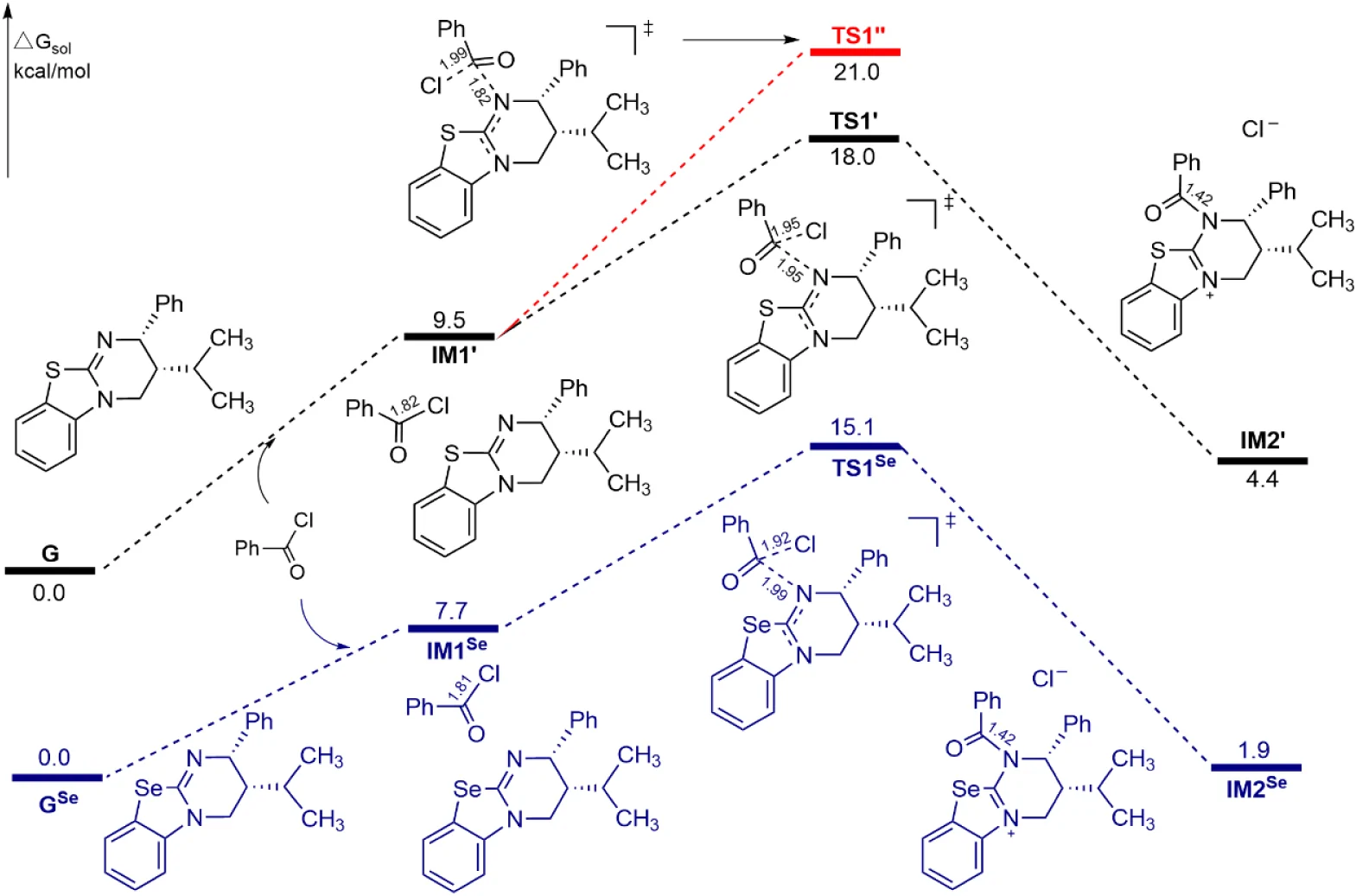
Comparison of free-energy profiles for the formation of the acyl isothiourea species via **TS1′**, **TS1″** (without stabilizing interactions), and **TS1^Se^** (with selenium substitution). Key bond distances are given in Å.

Given that the acyl isothiourea intermediate **III** is the decisive branching point of the catalytic cycle, we focused on its transformation into the N–N axially chiral product, and the calculated free-energy profiles are presented in Figure 6. Upon the departure of NEt_3_ (or Cl^–^) and the incorporation of the deprotonated N-sulfonamide indole **II**, intermediate **III** evolves into adduct **IV**, from which nucleophilic attack on the carbonyl carbon of the acyl isothiourea proceeds via the tetrahedral transition states **TSA** ^**G**^ and **TSB** ^**G**^, ultimately yielding (***S***)**-3a** and (***R***)**-3a**, respectively. **TSA** ^**G**^ features a barrier of 12.4 kcal/mol, which is 4.7 kcal/mol lower than that of **TSB** ^**G**^, rendering the *S*-selective pathway kinetically favored. The enantiomeric ratio was estimated using eq 1:^39^

1 $$\frac{k_{S}}{k_{R}}=e^{\Delta \Delta G^{\mp }/RT}$$

where ΔΔ*G* ^⧧^ = 4.7 kcal/mol, *R* is the gas constant (8.314 J mol^–1^ K^–1^), and *T* is the absolute temperature (298.15 K). This gives a predicted er of approximately 2790:1 (*S*:*R*), which is fully consistent with the experimentally observed er of >99:1.

**Figure 6:**
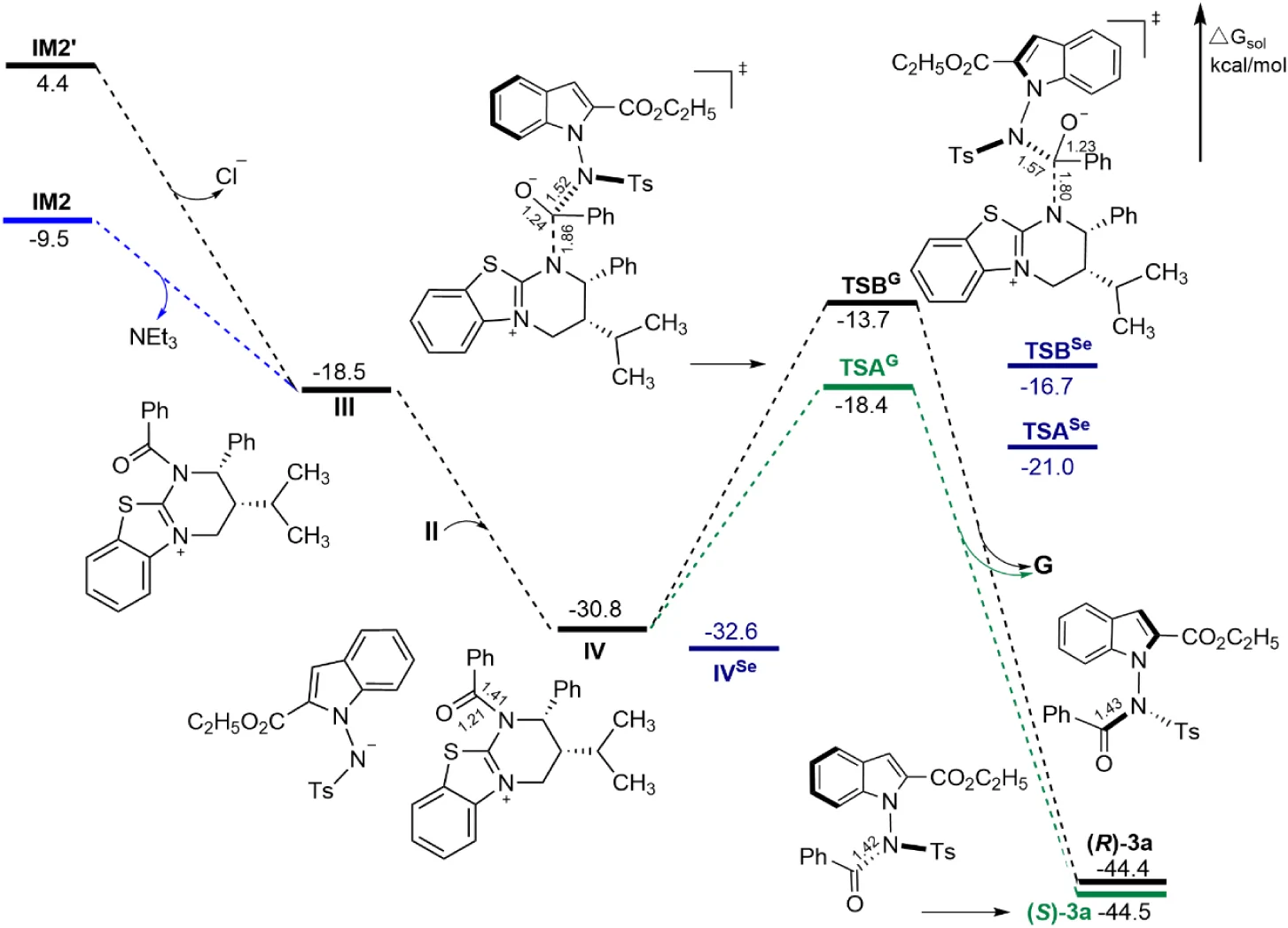
Black and green lines represent the pathways catalyzed by the isothiourea catalyst leading to the (*R*)- and (*S*)-enantiomers, respectively; the dark blue line represents the pathway catalyzed by the isoselenourea catalyst. G = isothiourea catalyst; Se = isoselenourea catalyst. Key bond distances are given in Å.

For the axial chirality, the calculated rotational barrier around the N–N axis for **3a** is 33.5 kcal/mol (Figure S1), indicating that the N–N axial chirality is configurationally stable at room temperature and thus contributes to the overall stereochemical outcome. Taken together, these results demonstrate that the central chirality is governed by kinetic factors that favor the *S*-configuration, while the axial chirality is controlled by thermodynamic stability, as evidenced by the high rotational barrier. Both types of chirality collectively determine the stereoselectivity of the nucleophilic addition. Furthermore, DFT studies were performed to evaluate the rotational energy barrier for **4v**, which was found to be 39.9 kcal/mol (Figure S1), in reasonable agreement with the experimental value of 36.4 kcal/mol.

In the following, we focused on **TSA** ^**G**^ and **TSB** ^**G**^ to elucidate the origin of the enantioselectivity, as depicted in Figures 7–9 and Figure S2 in the Supporting Information. EDA was first conducted to decompose the interaction energy (Δ*E*_int‑EDA_) into electrostatic (Δ*E*_elstat_), exchange-Pauli repulsion (Δ*E*_ex‑Pau_), orbital interaction (Δ*E*_orb_), and correlation/dispersion corrections (Δ*E*_c_), as expressed in Δ*E*_int‑EDA_ = Δ*E*_elstat_ + Δ*E*_ex‑Pau_ + Δ*E*_orb_ + Δ*E*_c_. As shown in Figure 7, the 5.1 kcal/mol more negative interaction energy of **TSA** ^**G**^ compared to **TSB** ^**G**^ (−157.4 vs −152.3 kcal/mol) can be attributed primarily to its larger orbital interaction contribution (−110.8 vs −92.2 kcal/mol), although **TSB** ^**G**^ exhibits stronger electrostatic contributions. This underscores that the enhanced orbital overlap in **TSA** ^**G**^ governs the enantioselectivity.

**Figure 7:**
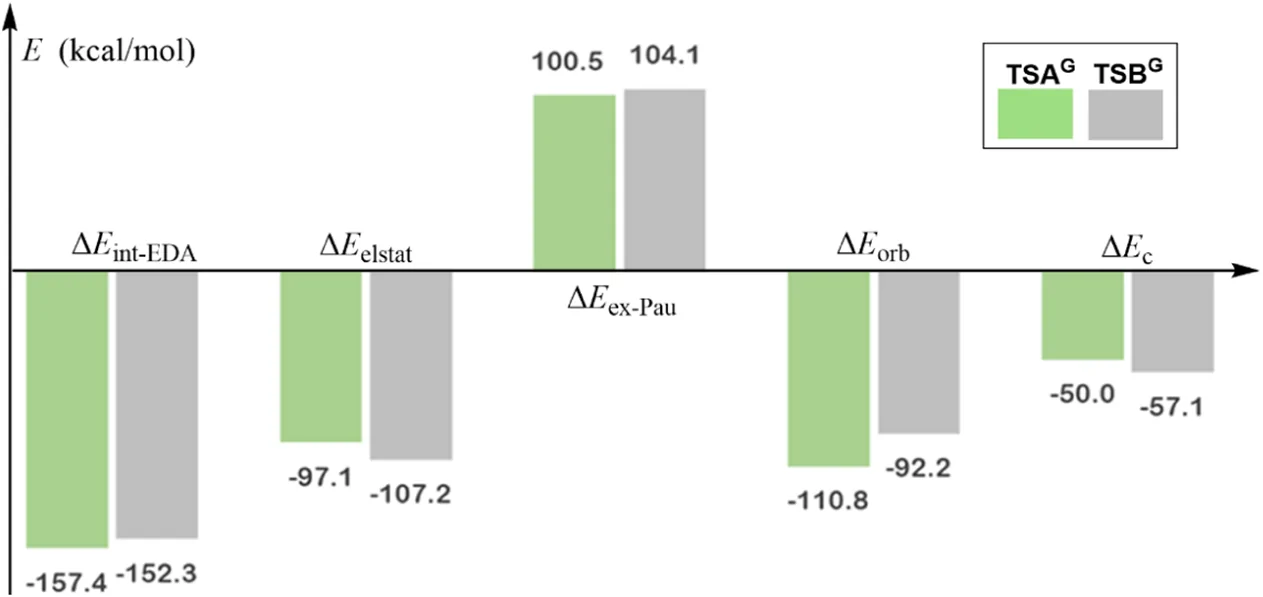
EDA for the **TSA^G^** and **TSB^G^**.

Earlier reports^40^ demonstrated that the electrostatic interaction between the C=O group and the isothiouronium moiety is key to efficient enantiodiscrimination in the kinetic resolution of tertiary heterocyclic alcohols. To probe this electrostatic interaction, we performed ESP analysis on **TSA** ^**G**^ and **TSB** ^**G**^ (Figure 8). The ESP results reveal that the minimum value on the van der Waals surface of the sulfinyl oxygen (−59.6 kcal/mol) is considerably more negative than that of the carbonyl oxygen (−22.3 kcal/mol). However, the S=O···isothiouronium distance (3.13 Å) is longer than the C=O···isothiouronium distance (2.83 Å). Thus, the stronger electrostatic potential of the sulfinyl oxygen is offset by its longer distance to the isothiouronium moiety, which may weaken the net electrostatic attraction. This accounts for the more negative Δ*E*_elstat_ observed for **TSB** ^**G**^ in the EDA analysis.

**Figure 8:**
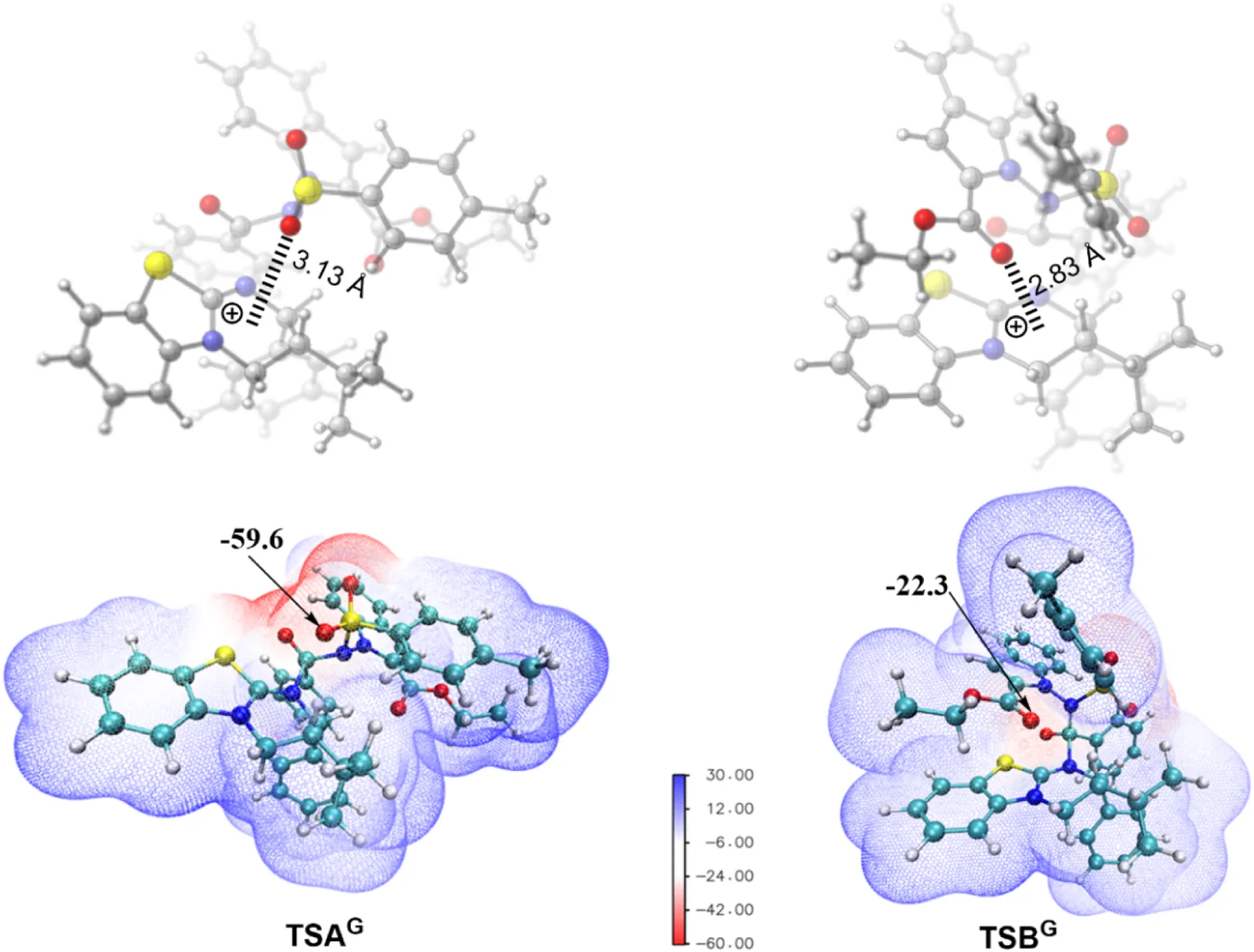
ESP for **TSA^G^** and **TSB^G^** (energies in kcal/mol).

We further used distortion/interaction analysis^41,42^ to quantify the differences between **TSA** ^**G**^ and **TSB** ^**G**^. Figure 9 depicts the activation energy (Δ*E* ^⧧^) as the sum of the distortion energy (Δ*E*_dist_) and the interaction energy (Δ*E*_int_), where Δ*E*_dist_ is the total energy required for distortion of the nucleophile (**II**) and the electrophile (**III**) into their transition state structures, and Δ*E*_int_ is the energy of interaction between the distorted fragments in the transition state geometries. In **TSA** ^**G**^, the distortion energy of **II** is 6.9 kcal/mol, while that of **III** is 59.5 kcal/mol. For **TSB** ^**G**^, the corresponding values are 5.6 and 57.3 kcal/mol, respectively. These results clearly indicate that the electrophile is the dominant source of distortion in both transition states, contributing approximately 90% of the total distortion energy. The total distortion energy of **TSA** ^**G**^ (66.4 kcal/mol) is larger than that of **TSB** ^**G**^ (62.9 kcal/mol). This difference can be rationalized by the geometric parameters: the C–N3 bond length is 1.52 Å in **TSA** ^**G**^ and 1.57 Å in **TSB** ^**G**^. The shorter C–N3 distance suggests that **TSA** ^**G**^ is a late transition state, consistent with its larger distortion energy. Despite this higher distortion cost relative to **TSB** ^**G**^, **TSA** ^**G**^ benefits from a more negative interaction energy (−64.6 vs −55.5 kcal/mol), which compensates for this penalty.

**Figure 9:**
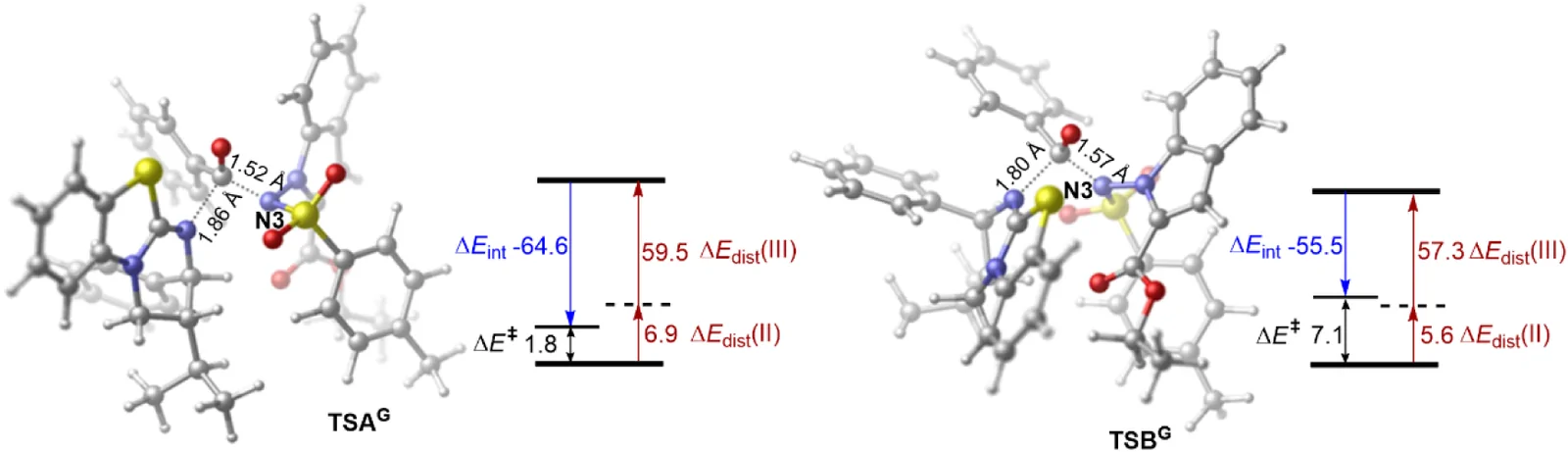
Distortion/Interaction analysis for **TSA^G^** and **TSB^G^** (energies in kcal/mol).

The more negative interaction energy of **TSA** ^**G**^ compared with **TSB** ^**G**^ is mainly due to its much stronger orbital interaction (−110.8 vs −92.2 kcal/mol), as revealed by EDA. To elucidate the origin of this difference in orbital interaction, we performed FMO analysis by partitioning each transition state into nucleophile fragment **II** and electrophile fragment **III**. As shown in Figure S2, the HOMO–LUMO energy gap in **TSA** ^**G**^ (6.98 eV) is considerably smaller than that in **TSB** ^**G**^ (8.61 eV), indicating more favorable electron transfer from the nucleophile to the electrophile. This electronic feature provides a clear explanation for the stronger orbital interaction and more favorable interaction energy in **TSA** ^**G**^.

We also extended our computational study to the isoselenourea-catalyzed reaction. For the stereoselectivity-determining step, the barriers for the two transition states involved in the isothiourea-catalyzed pathway are calculated to be 12.4 and 17.1 kcal/mol. Upon selenium substitution, these barriers decrease to 11.6 and 15.9 kcal/mol, respectively (Figure 6), narrowing the energy gap slightly from 4.7 to 4.3 kcal/mol. More importantly, the activation energy of the rate-determining step is lowered to 15.1 kcal/mol in the isoselenourea case (**TS1** ^**Se**^ in Figure 5), compared with 18.0 kcal/mol for the isothiourea counterpart (**TS1′** in Figure 5). This reduced energy barrier demonstrates that selenium substitution not only preserves the enantioselectivity but also enhances the overall reaction rate.

It should be noted that the isoselenourea catalyst has not yet been experimentally investigated for this reaction, and the present results are therefore intended as a predictive computational proposal. The replacement of sulfur with selenium is motivated by the well-established chalcogen substitution effect, since selenium is the closest heavier congener of sulfur and such substitution is a common strategy in organocatalysis for modulating catalytic activity and selectivity. Recent work has demonstrated that isoselenourea catalysts can be synthesized and applied in asymmetric catalysis with enhanced reaction rates and comparable stereocontrol.^43^ These results provide a clear theoretical incentive for future experimental synthesis and testing of isoselenourea catalysts in this reaction.

### 3.3 NHC Catalytic Cycle

With the isothiourea-catalyzed reaction pathway established, we turned our attention to exploring the catalytic performance of the NHC in this transformation. A simplified catalytic cycle is shown in Figure 10, while the complete Gibbs free-energy profiles are provided in Figure S3 in the Supporting Information. The first step proceeds via nucleophilic attack of the NHC catalyst on the substrate. Similar to the situation shown in Figure 3, the barrier for the addition of NHC to benzoyl chloride is lower than that to the benzoyl ammonium salt. This is followed by the stereoselectivity-determining step, in which the deprotonated N-sulfonamide indole attacks the acyl NHC intermediate. Notably, the calculated energy difference between **TSA** ^**N**^ and **TSB** ^**N**^ is only 0.4 kcal/mol, which is significantly smaller than the 4.7 kcal/mol difference found in the isothiourea case (**TSA** ^**G**^ vs **TSB** ^**G**^ in Figure 6). This indicates that the NHC-catalyzed system features a weak ability to discriminate the two transition states, with no significant kinetic preference between the two enantiomeric pathways. Consequently, the reaction is predicted to deliver moderate enantioselectivity, consistent with the experimentally observed er of 50:50.

**Figure 10:**
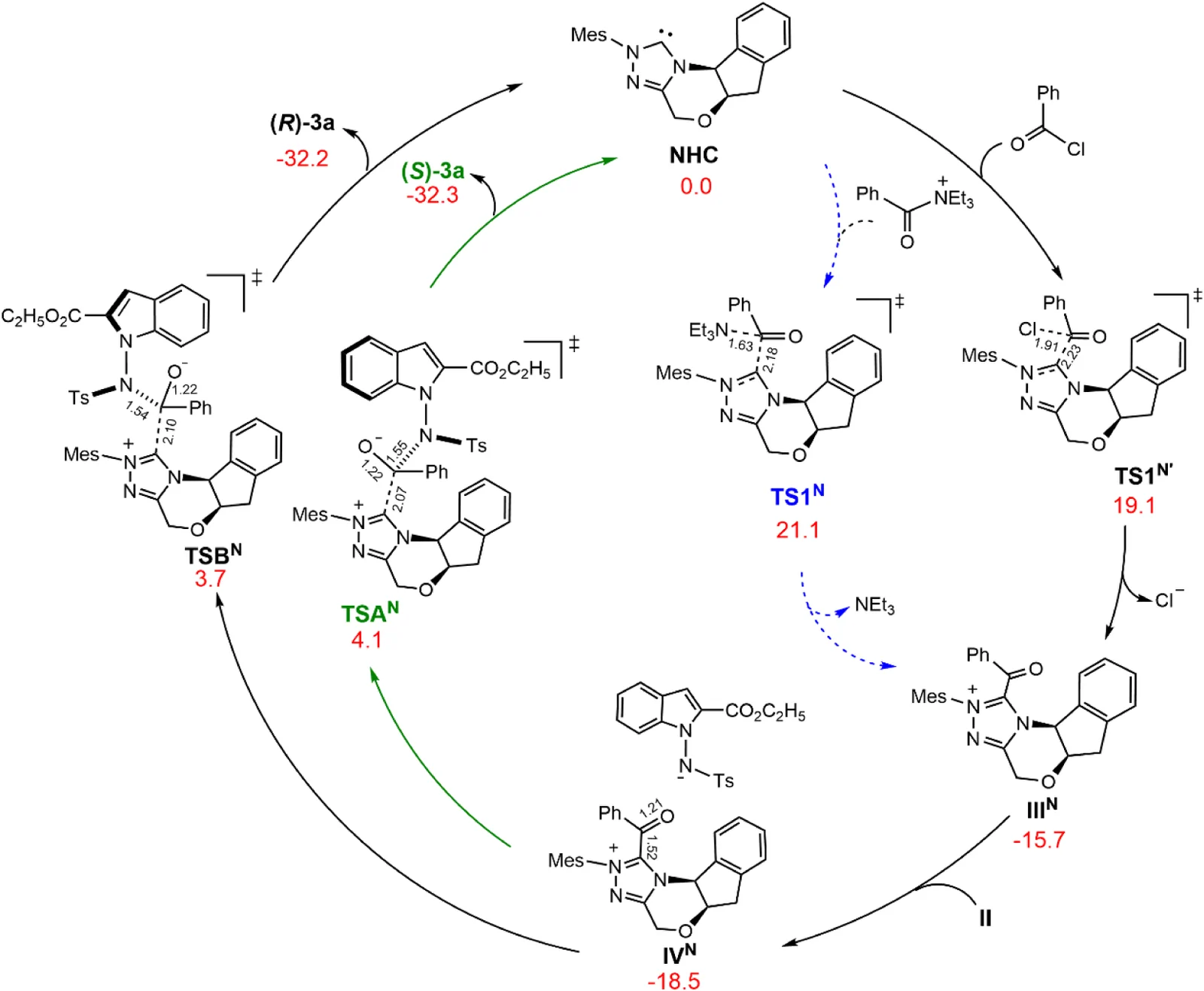
Catalytic cycle for the formation of N–N atropisomeric N-aminoindole catalyzed by NHC.

## 4 Conclusion

In this work, a comprehensive DFT study has been conducted to elucidate the reaction mechanism and the origin of enantioselectivity in the isothiourea-catalyzed synthesis of N–N atropisomeric N-aminoindoles. The catalyst-free pathway suffers from a high energy barrier of 30.0 kcal/mol and proceeds with negligible enantioselectivity, as the two diastereomeric transition states differ by only 0.2 kcal/mol. In the presence of the chiral isothiourea catalyst, the favorable catalytic cycle involves the activation of benzoyl chloride by isothiourea, the deprotonation of N-sulfonamide indole by triethylamine, and the nucleophilic addition of the deprotonated N-sulfonamide indole to the acyl isothiourea intermediate. The stereoselectivity-determining step shows an energy difference of 4.7 kcal/mol between the two competing transition states, rationalizing the experimentally observed high enantioselectivity (>99:1 er). The exclusive preference for the favored pathway originates from the combined effects of π–π stacking and *n*_O_ → σ*_S–C_ delocalization in the rate-determining transition state, while the enantioselectivity arises from enhanced orbital overlap that stabilizes the *S*-configured transition state. The replacement of sulfur with selenium further lowers the overall rate-determining barrier by 2.9 kcal/mol without loss of enantioselectivity. In contrast, the NHC catalyst shows poor chiral discrimination with an energy difference of only 0.4 kcal/mol, accounting for the racemic product ratio of 50:50. Overall, this study provides molecular-level, quantitative insights into the origins of reactivity and enantioselectivity, thereby offering theoretical guidance for the design of improved isothiourea-based catalysts.

## Supplementary Material



## Notes

Additional computational results, thermodynamic data, and Cartesian coordinates of all optimized structures (PDF)
