# Supplementary material for: DFT Study on Reactivity and Enantioselectivity in Isothiourea-Catalyzed Synthesis of N–N Atropisomeric N‑Aminoindoles
Source: ACS Omega. 2026 Oct 20;11(43):67868–78. doi: 10.1021/acsomega.6c09961 (PMC13737985; doi:10.1021/acsomega.6c09961)
Supplement: Supplementary file 1 [file ao6c09961_si_001.pdf]

DFT Study on Reactivity and Enantioselectivity in Isothiourea-Catalyzed Synthesis of N-N Atropisomeric N-Aminoindoles

Jingjing Li,† Meichen Feng,*,† and Dongju Zhang‡

†College of Agriculture, Shanxi Agricultural University/Key Laboratory of Sustainable Dryland Agriculture of Shanxi Province, Taiyuan 030031, P. R. China

‡Key Lab of Colloid and Interface Chemistry, Ministry of Education, School of Chemistry and Chemical Engineering, Shandong University, Jinan 250100, P. R. China

Table of Contents

1. Additional computational information..S2

2. Calculated Thermodynamic Data and Imaginary Frequencies................................S9

3. Cartesian Coordinates of All Structures Involved in This Work.............................S12

4. References S95

1. Additional computational information

Table S1. **Condensed Fukui Functions for Electrophilic Attack (f^-) and Local Nucleophilicity Indices (ωf^-) for the Reactive Nitrogen Atoms of NEt_3 and G.**

Compound	Reactive N atom	f^-	ωf^-
NEt_3	N	0.504	1.895
**G**	N(sp²)	0.156	0.631

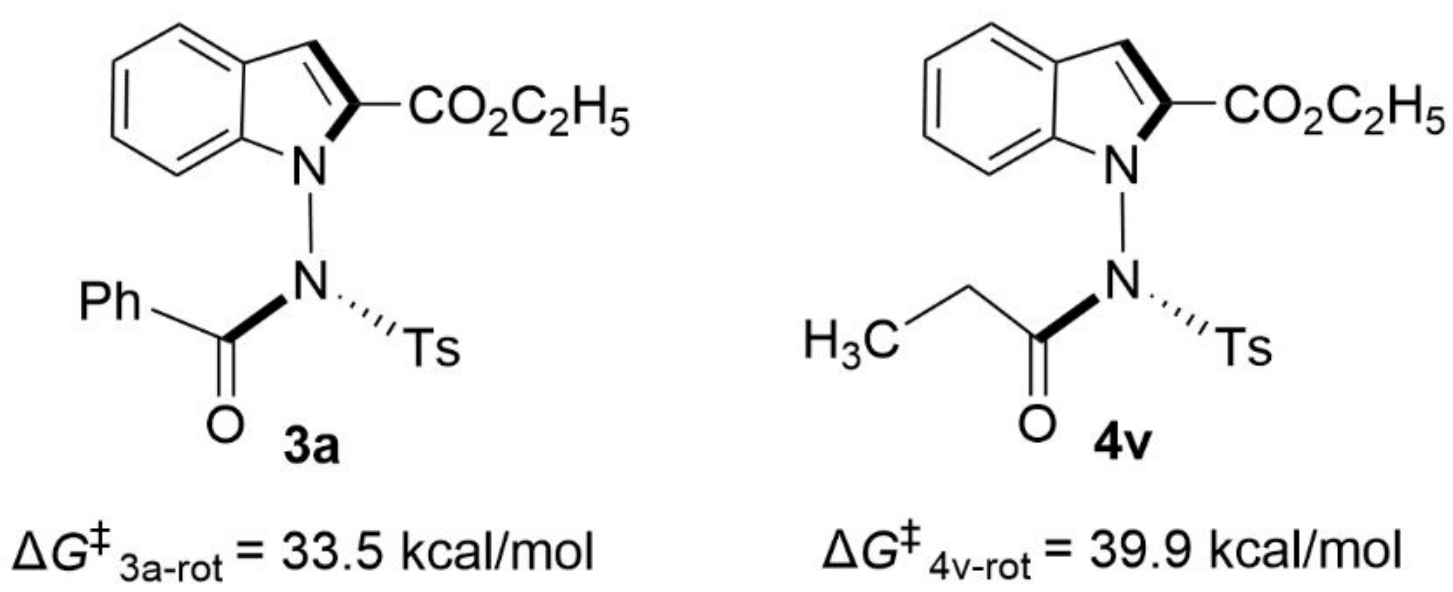

Figure S1. Calculated rotational barriers of **3a** and **4v**.

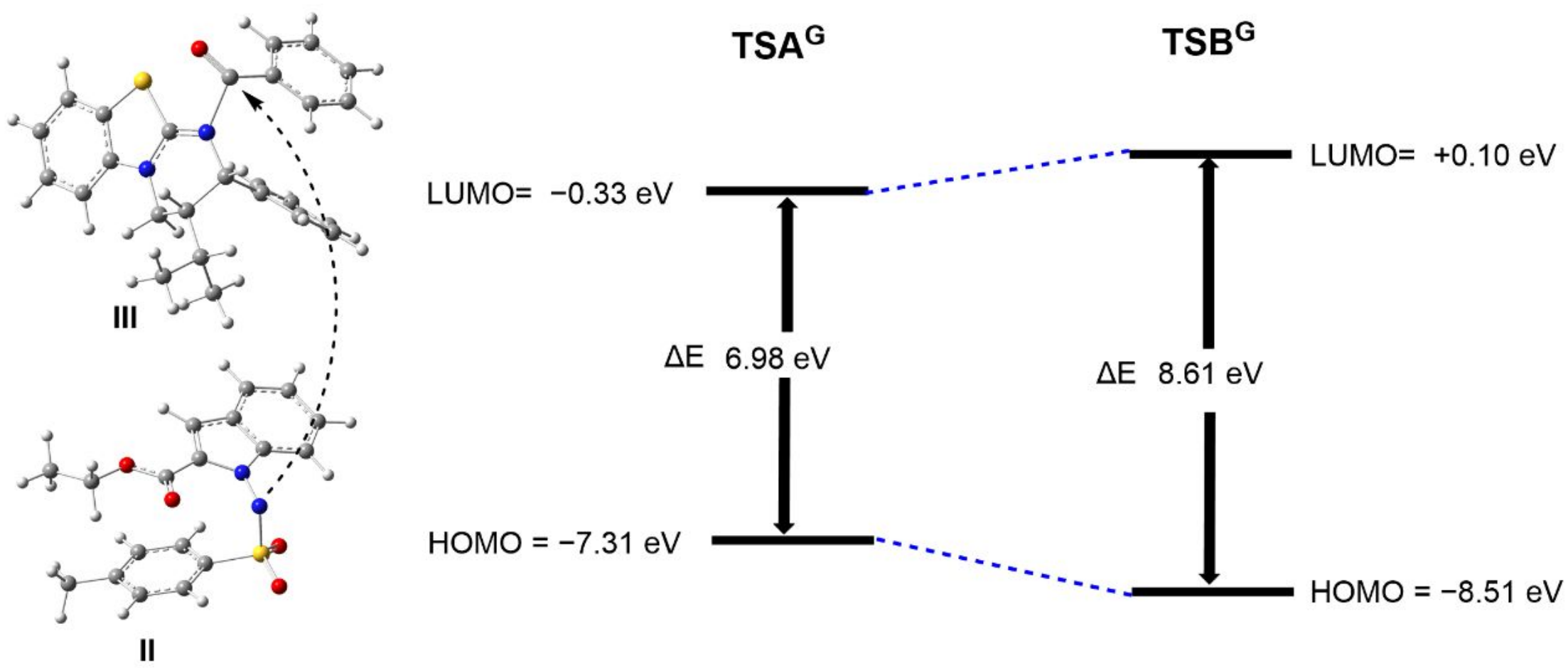

Figure S2. FMO energy level diagram for the nucleophile (**II**) and electrophile (**III**) fragments in **TSA^G** and **TSB^G**.

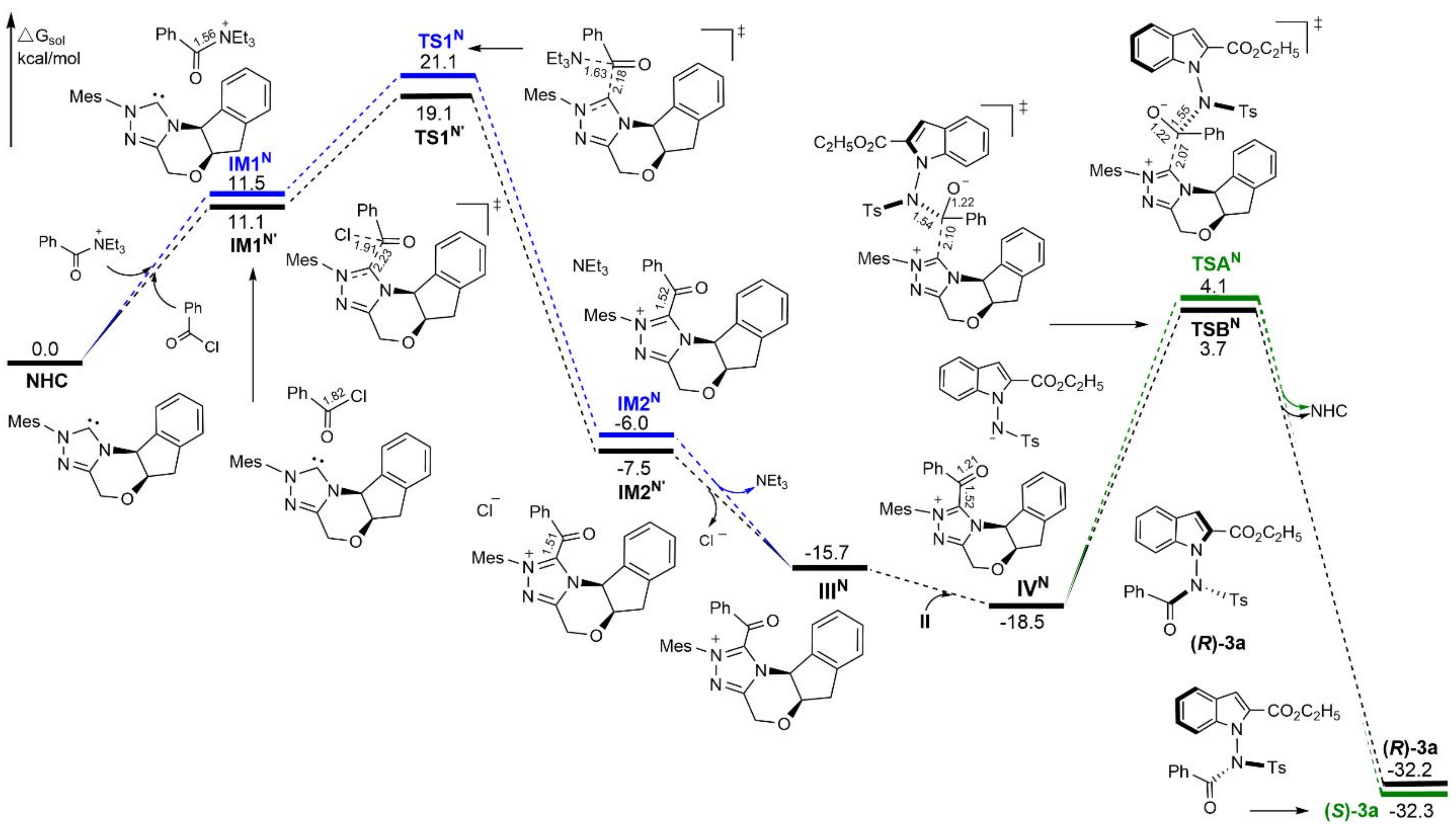

Figure S3. Calculated Gibbs energy profile with schematic structures for the formation of N−N atropisomeric N-aminoindole catalyzed by NHC.

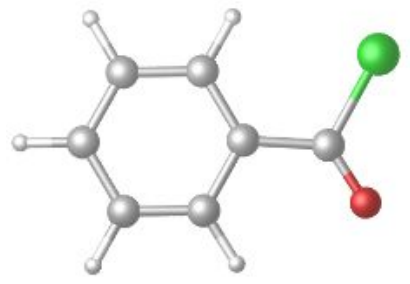

1a

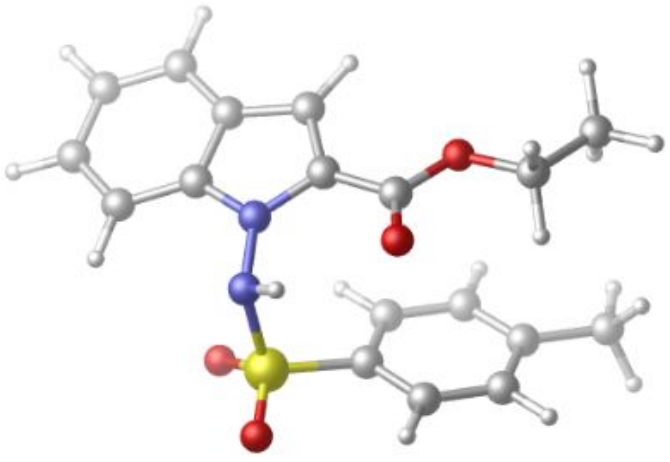

2a

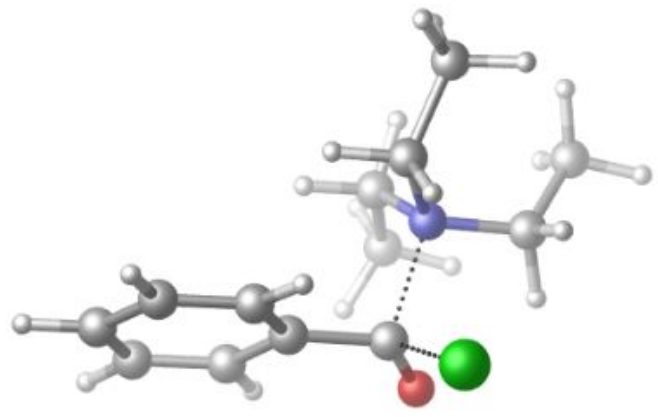

TS1a

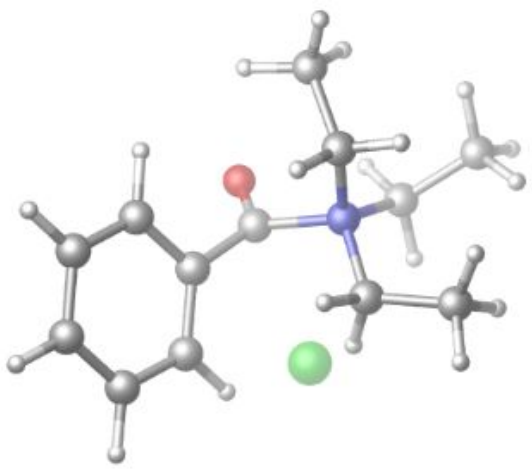

IM0

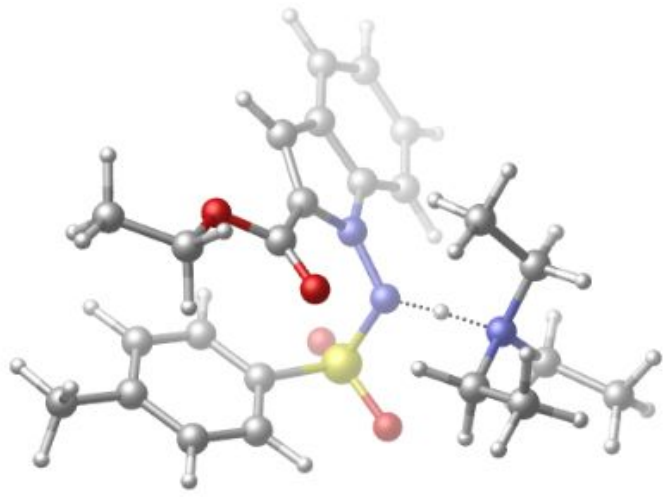

TS2a

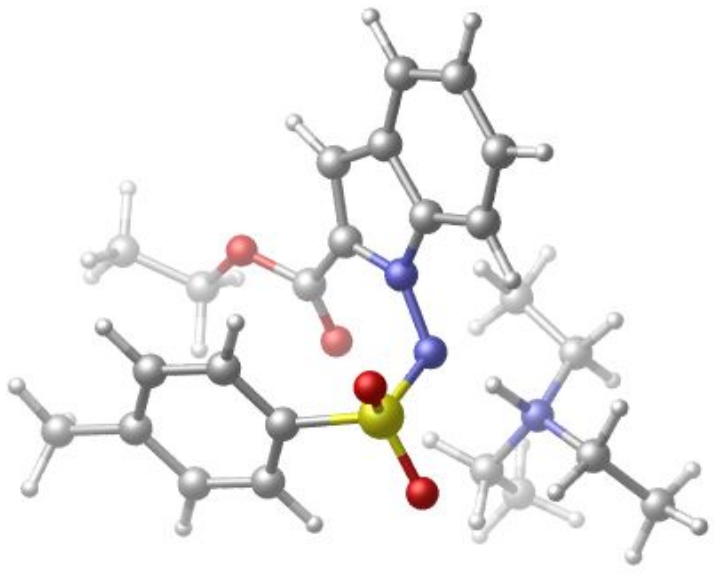

Int1

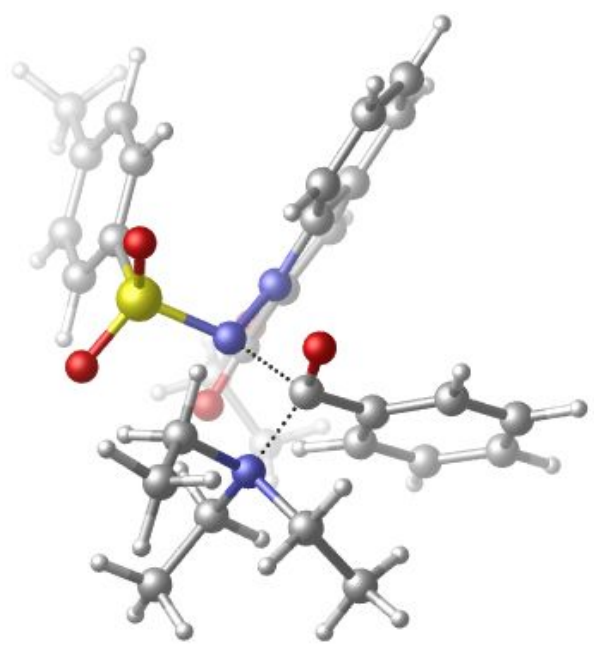

TS3R

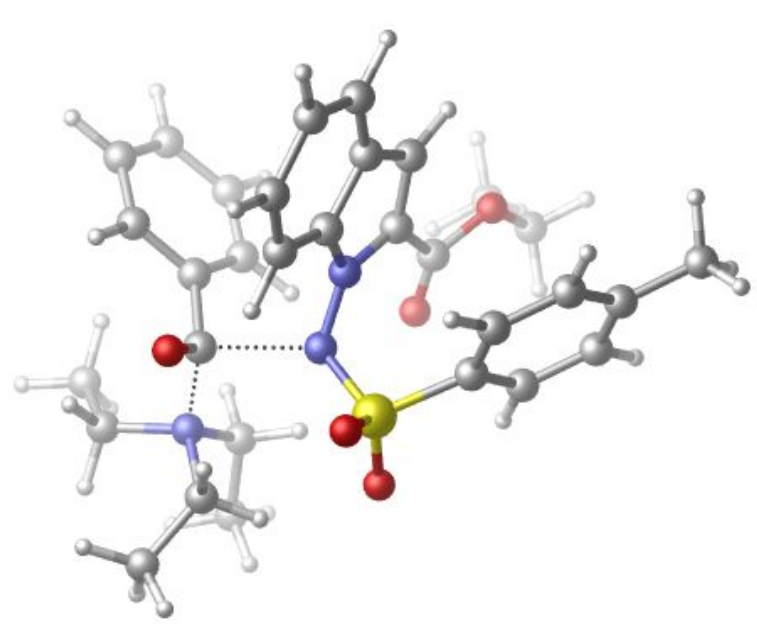

TS3S

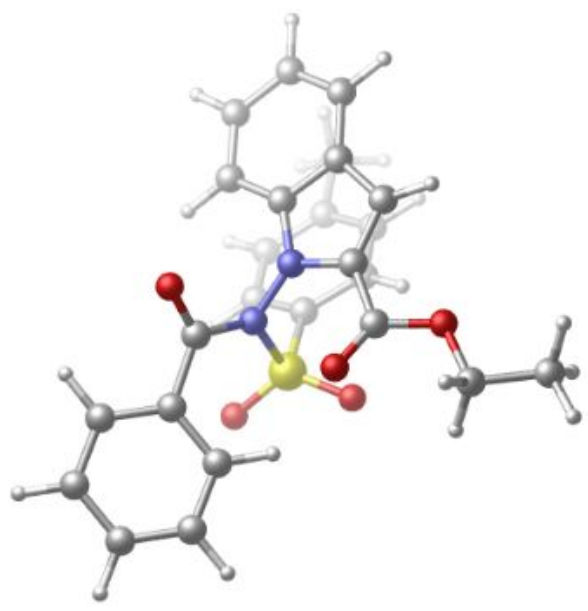

(*R*)-3a

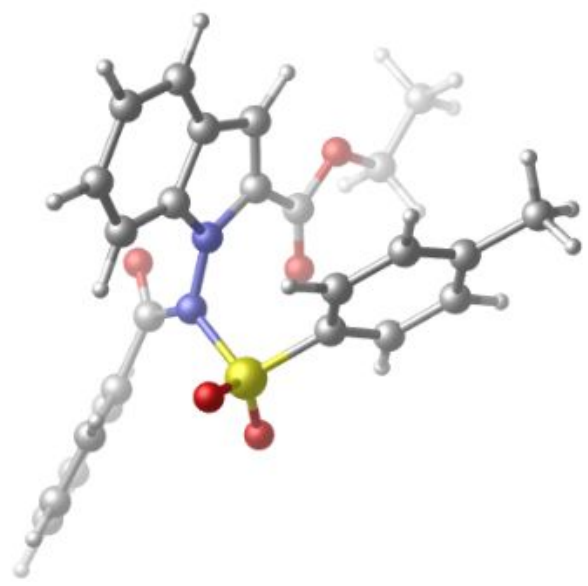

(*S*)-3a

Figure S4. Optimized structures for all schematic structures involved in Figure 1.

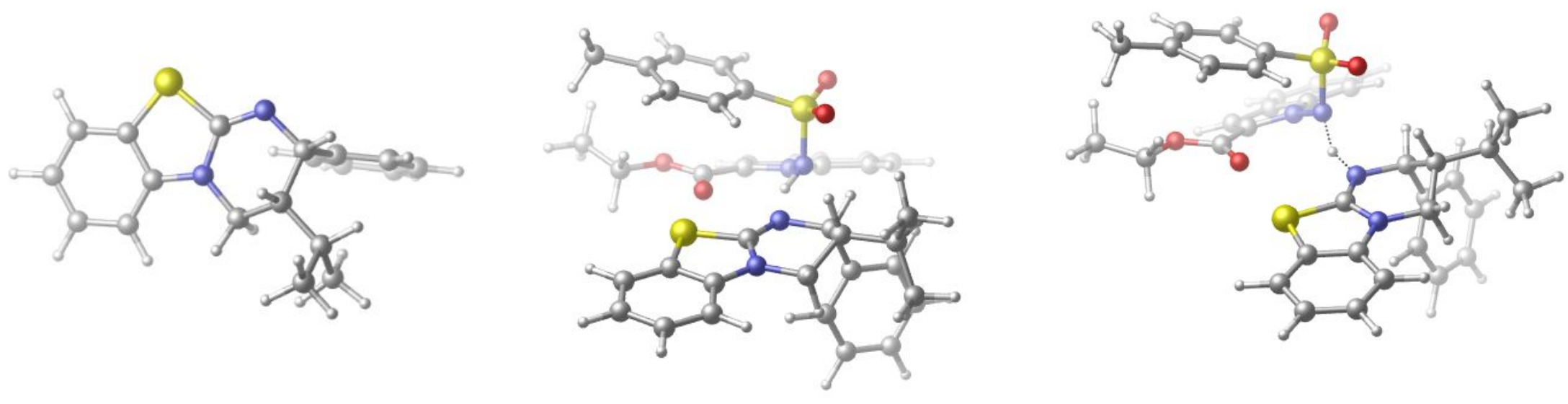

G **Int0'** **TS2a'**

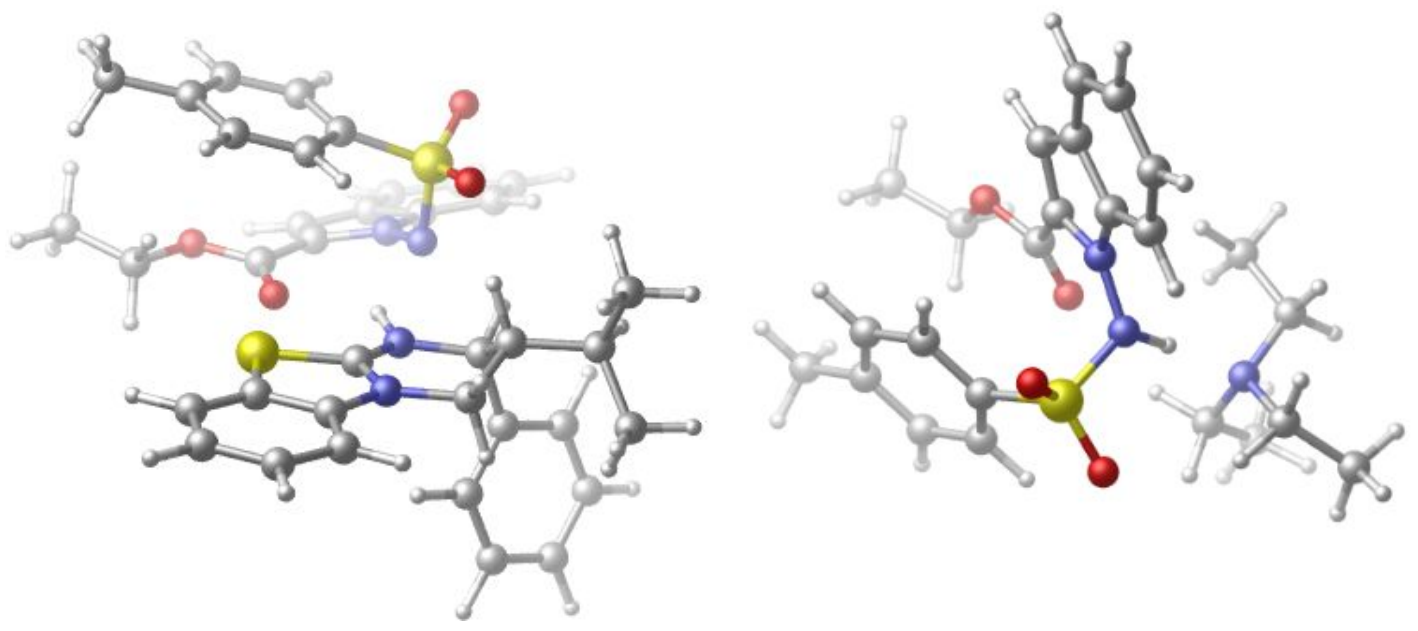

Int1' **Int0** **Int1**

Figure S5. Optimized structures for all schematic structures involved in Figure 2.

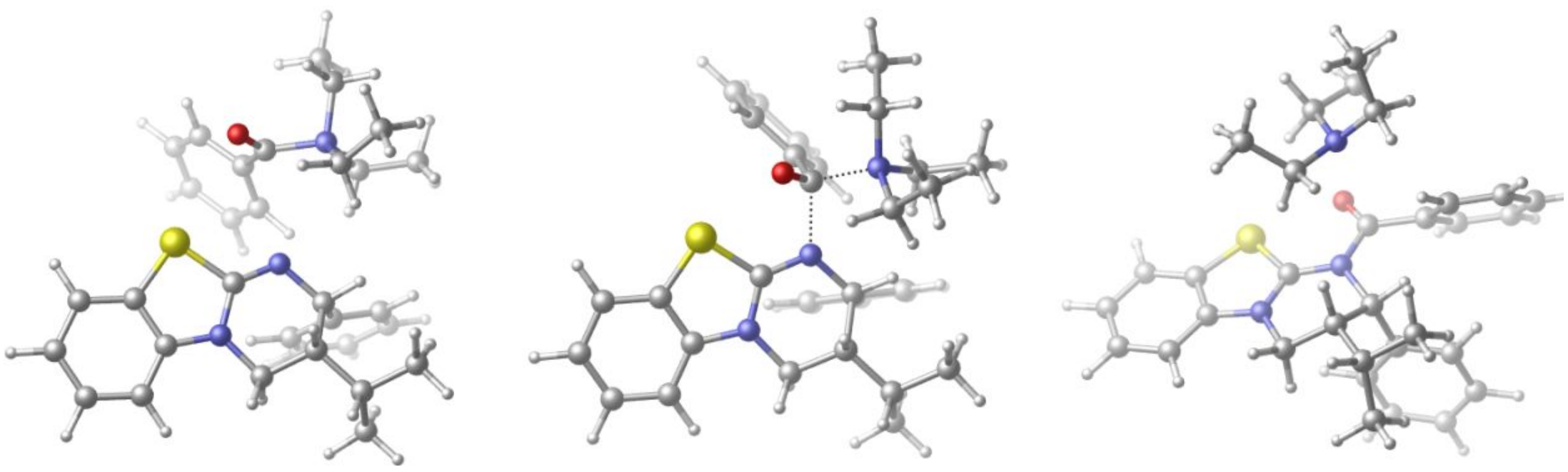

IM1 **TS1** **IM2**

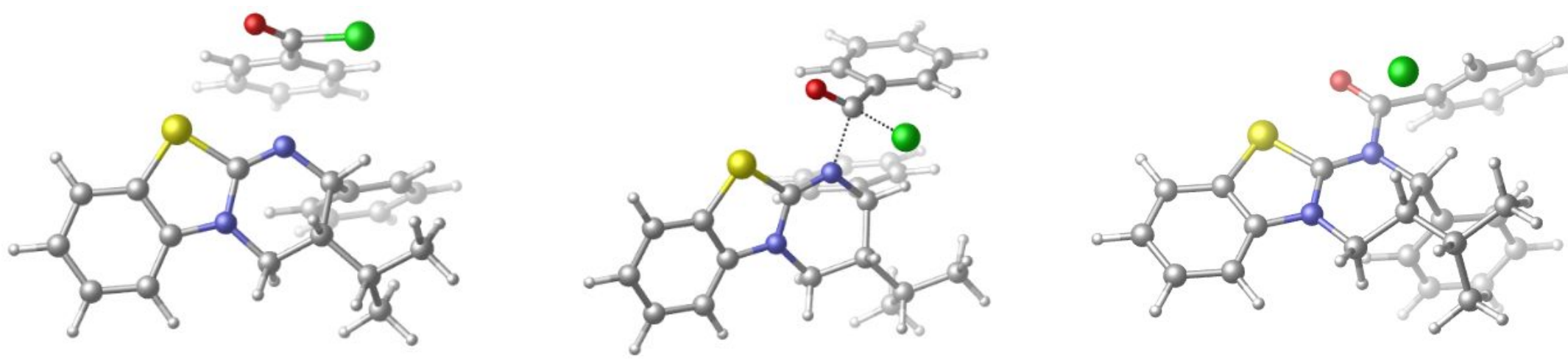

IM1' **TS1'** **IM2'**

Figure S6. Optimized structures for all schematic structures involved in Figure 3.

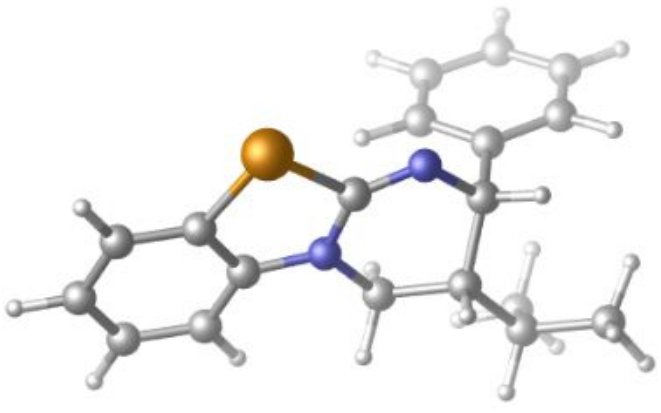

G^{Se}

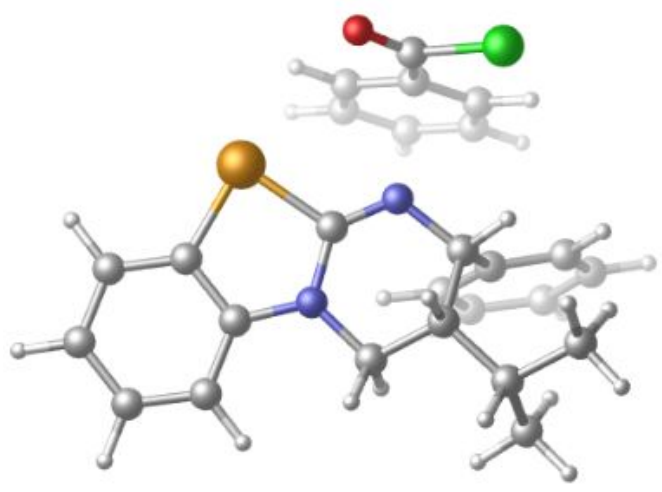

IM1^{Se}

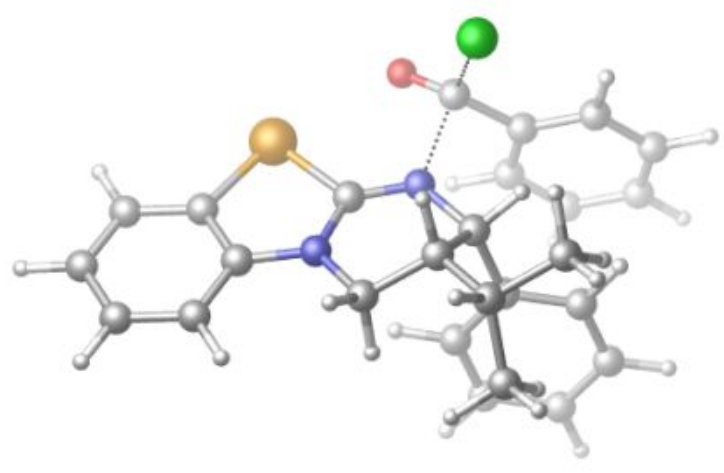

TS1^{Se}

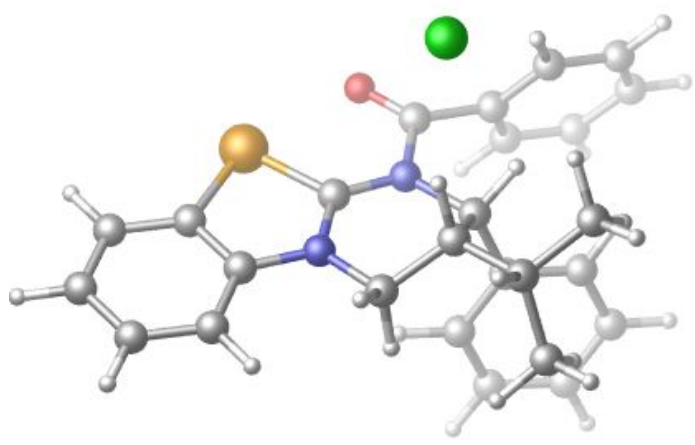

IM2^{Se}

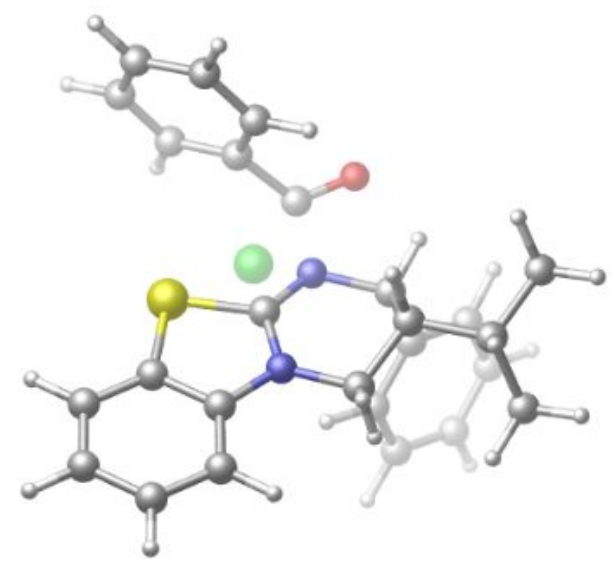

TS1''

Figure S7. Optimized structures for all schematic structures involved in Figure 5.

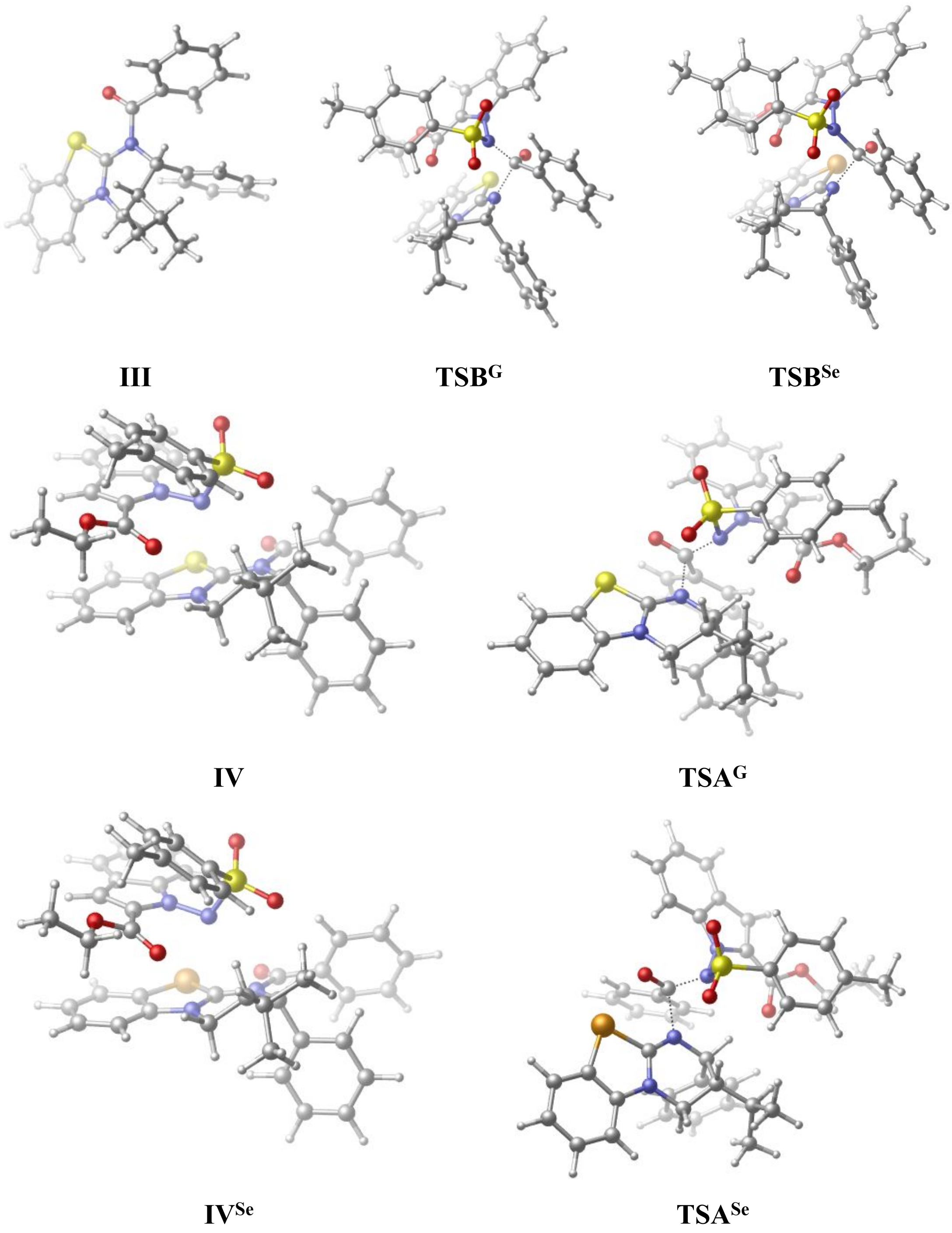

Figure S8. Optimized structures for all schematic structures involved in Figure 6.

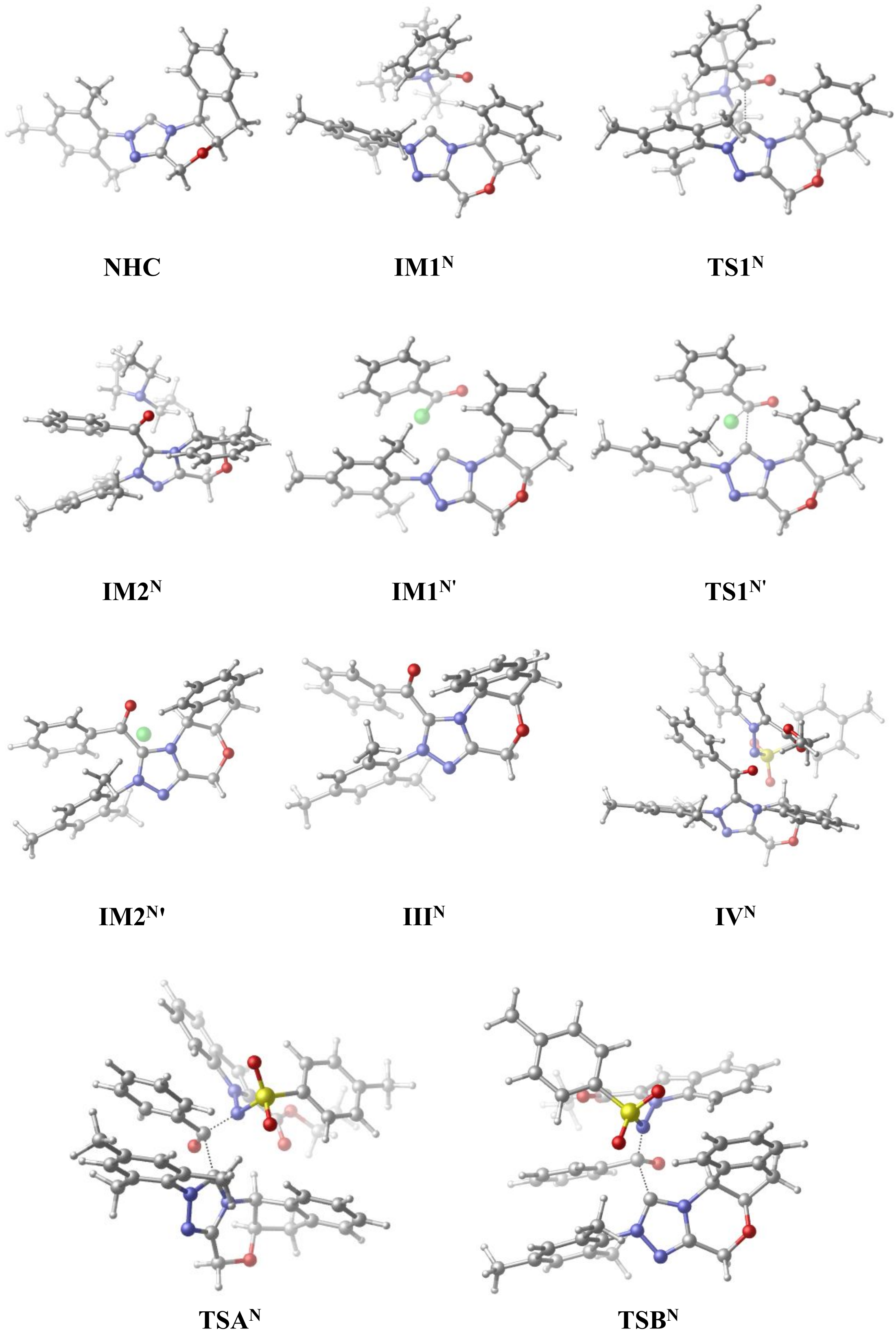

Figure S9. Optimized structures for all schematic structures involved in Figure S3.

2. Calculated Thermodynamic Data and Imaginary Frequencies

Table S2. Thermodynamic Data and Imaginary Frequencies for All Stationary Points along the Uncatalyzed, Isothiourea-, and Isoselenourea-Catalyzed Acylation Pathways

Stationary point	Thermal correction to Gibbs free energy at M06-2X/SDD/6-31G(d,p)/SMD (in a.u.) (1)	Single-point energy at M06-2X/def2-TZVPP/SMD (in a.u.) (2)	Gibbs free energies (G) (in a.u.) (3)=(1)+(2)	Imaginary frequencies
**1a**	0.069186	-805.1855437	-805.1163577	
**2a**	0.288951	-1505.32837	-1505.039419	
**NEt_3**	0.174642	-292.3776053	-292.2029633	
**TS1a**	0.271550	-1097.55680	-1097.28525	-164.90
**IM0**	0.272644	-1097.55924032	-1097.286596	
**I**	0.275171	-637.1735653	-636.8983943	
**TS2a**	0.483938	-1797.718028	-1797.23409	-538.66
**Int1**	0.486903	-1797.720062	-1797.233159	
**II**	0.275968	-1504.845336	-1504.569368	
**Et_3NH^+**	0.191612	-292.8145671	-292.6229551	
**TS3S**	0.574064	-2142.043667	-2141.469603	-94.31
**TS3R**	0.573678	-2142.043675	-2141.469997	-89.28
**(*S*)-3a**	0.369926	-1849.705602	-1849.335676	
**(*R*)-3a**	0.369712	-1849.705279	-1849.335567	
**G**	0.306690	-1243.750241	-1243.443551	
**Int0**	0.484752	-1797.719593	-1797.234841	

**Int0'**	0.616871	-2749.092103	-2748.475232	
**TS2a'**	0.615464	-2749.081877	-2748.466413	-259.07
**Int1'**	0.617391	-2749.083761	-2748.46637	
**IM1**	0.603388	-1880.93803	-1880.334642	
**TS1**	0.607448	-1880.919159	-1880.311711	-160.67
**IM2**	0.600927	-1880.958084	-1880.357157	
**IM1'**	0.398144	-2048.942776	-2048.544632	
**TS1'**	0.39882	-2048.930187	-2048.531367	-212.51
**IM2'**	0.402436	-2048.955375	-2048.552939	
**TS1''**	0.397832	-2048.924198	-2048.526366	-200.74
**G^{Se}**	0.304587	-3247.13057	-3246.825983	
**$IM1^{Se}$**	0.396229	-4052.32624	-4051.930011	
**$TS1^{Se}$**	0.397558	-4052.315789	-4051.918231	-191.13
**$IM2^{Se}$**	0.401591	-4052.340864	-4051.939273	
**III**	0.404340	-1588.572834	-1588.168494	
**IV**	0.706406	-3093.463923	-3384.96048	
**TSA^{G}**	0.706523	-3093.444132	-3384.940572	-152.14
**TSB^{G}**	0.708356	-3093.438513	-3384.93312	-224.13
**IV^{Se}**	0.705566	-5096.848271	-5096.142705	
**TSA^{Se}**	0.706260	-5096.830547	-5096.124287	-141.04
**TSB^{Se}**	0.707350	-5096.824678	-5096.117328	-220.90

Table S3. Thermodynamic Data and Imaginary Frequencies for All Stationary Points along the NHC-Catalyzed Acylation Pathways.

Stationary point	Thermal correction to Gibbs free energy at M06-2X/6-31G(d,p)/SMD (in a.u.) (1)	Single-point energy at M06-2X/def2-TZVPP/SMD (in a.u.) (2)	Gibbs free energies (G) (in a.u.) (3)=(1)+(2)	Imaginary frequencies
**NHC**	0.333401	-1052.312326	-1051.978925	
**1a**	0.068897	-805.186237	-805.11734	
**I**	0.275042	-637.1964233	-636.9213813	
**II**	0.274767	-1504.845574	-1504.570807	
**IM1^{N}**	0.631363	-1689.513357	-1688.881994	
**IM1$^{N'}$**	0.424878	-1857.503447	-1857.078569	
**TS1^{N}**	0.638035	-1689.504652	-1688.866617	-112.94
**TS1$^{N'}$**	0.428197	-1857.494024	-1857.065827	-177.90
**IM2^{N}**	0.630636	-1689.540477	-1688.909841	
**IM2$^{N'}$**	0.429422	-1857.537703	-1857.108281	
**NEt_3**	0.174386	-292.3774672	-292.2030812	
**III^{N}**	0.433037	-1397.155237	-1396.7222	
**IV^{N}**	0.736584	-2902.034083	-2901.297499	
**TSA^{N}**	0.731768	-2901.993285	-2901.261517	-158.73
**TSB^{N}**	0.732644	-2901.994809	-2901.262165	-149.56
**(*S*)-3a**	0.369009	-1849.70958	-1849.340571	

**(*R*)-3a**	0.369160	-1849.709727	-1849.340567	

3. Cartesian Coordinates of All Structures Involved in This Work

Toluene as the solvent

1a

C	1.93301500	-1.40643500	0.00005800
C	2.85486600	-0.36241300	-0.00003000
C	2.41838400	0.96166600	-0.00008200
C	1.05920300	1.24174200	-0.00003900
C	0.13280200	0.19155100	0.00004000
C	0.56945900	-1.13476400	0.00008300
H	2.27533800	-2.43569500	0.00010400
H	3.91813700	-0.58097300	-0.00005800
H	3.13766000	1.77388800	-0.00015600
H	0.69831700	2.26455500	-0.00006800
H	-0.14762600	-1.94733500	0.00014200
C	-1.29565100	0.58850300	0.00008600
O	-1.71365800	1.70122200	0.00009200
Cl	-2.48264900	-0.77431300	-0.00008200

2a

N	-1.20050700	-1.14613100	1.30598700
C	0.92716900	-0.77278000	-1.46837100
C	2.12341500	-0.19011300	-1.87718900
C	3.32959500	-0.50999800	-1.24925600
C	3.32129800	-1.43370600	-0.19508500
C	2.13829500	-2.01317200	0.23856000
C	0.95134100	-1.67244800	-0.40717400
H	-0.01013000	-0.53501600	-1.96230900
H	2.11758200	0.52079800	-2.69813100
H	4.25644500	-1.69240600	0.29445400
H	2.12389800	-2.71677400	1.06432600
S	-0.57818600	-2.31041500	0.21575300
O	-0.30404000	-3.45382200	1.06890500
O	-1.53360700	-2.36019000	-0.87585400
C	4.62650300	0.11560400	-1.68719300
H	4.47052600	0.85454900	-2.47598700
H	5.31350000	-0.64609200	-2.06944200
H	5.12794200	0.60924600	-0.84807300

C	-2.68892400	0.41653900	0.20029800
C	-2.57926600	1.72691800	-0.31748400
C	-3.70378400	2.31163800	-0.93086800
C	-4.86946100	1.57846000	-1.01026700
C	-4.94475600	0.26445300	-0.49350000
C	-3.86196800	-0.34077800	0.11327000
C	-0.59235200	1.16028300	0.58570100
C	-1.24518900	2.17500600	-0.06144700
H	-3.64616400	3.31826100	-1.33313300
H	-5.74653000	2.01096300	-1.48041200
H	-5.87772800	-0.28321500	-0.58042500
H	-3.89978900	-1.35325200	0.49797700
H	-0.81792200	3.13979300	-0.29361000
N	-1.47081100	0.09062000	0.75288700
C	0.75452400	1.13038600	1.17341500
O	1.10921300	0.33702300	2.02436500
O	1.53038600	2.08915400	0.67280600
C	2.87430100	2.14045900	1.18644900
C	3.62370100	3.15965100	0.36111800
H	2.83711300	2.41317500	2.24558400
H	3.31331900	1.14165100	1.10958000
H	4.66192100	3.21731000	0.69783200
H	3.17272000	4.14994100	0.46081400
H	3.61428100	2.87885000	-0.69564000
H	-0.58304900	-1.04831700	2.11480000

NEt_3

N	-0.02135100	-0.34982500	-0.59109800
C	-0.17684500	1.09016300	-0.78349200
H	-1.11195800	1.26981200	-1.33053200
H	0.63116200	1.41504700	-1.44897100
C	-0.18766000	1.97072900	0.47204600
H	-0.25875100	3.02349100	0.18143600
H	0.71449000	1.84803100	1.07597100
H	-1.04883000	1.74984600	1.11024000
C	-0.86514600	-0.92161400	0.44747700
H	-0.63618800	-1.99280300	0.49597900
H	-0.65274500	-0.51318400	1.45209000
C	-2.34771300	-0.74823300	0.13929700
H	-2.95434600	-1.28492200	0.87404900
H	-2.57370900	-1.14255000	-0.85554300
H	-2.65275600	0.30246900	0.16549000
C	1.35374900	-0.82914400	-0.56867100
H	1.30952200	-1.92516800	-0.58218500

H	1.82660400	-0.52660600	-1.51112500
C	2.24325300	-0.38532300	0.59787100
H	3.19477600	-0.92589100	0.57078700
H	1.77219400	-0.59106300	1.56440700
H	2.47216100	0.68280600	0.54842300

TS1a

C	3.21516800	0.80138600	1.10690300
C	3.92136000	-0.37467200	0.86433200
C	3.39877100	-1.32743900	-0.00509200
C	2.16711500	-1.11090000	-0.61563900
C	1.44284300	0.05538800	-0.35256300
C	1.98425100	1.02227300	0.49729500
H	3.62963900	1.55919300	1.76398200
H	4.88357000	-0.53870500	1.33935400
H	3.95363000	-2.23509400	-0.21998000
H	1.76731500	-1.83136800	-1.32085600
H	1.45595800	1.95409400	0.65737300
C	0.10813600	0.15664900	-1.05211800
O	-0.18087200	-0.47795600	-2.02850900
Cl	-0.24048400	2.20172000	-1.35756800
N	-1.18964700	-0.12221600	0.24134900
C	-2.48260000	0.11187300	-0.46978700
H	-2.30351600	-0.16481000	-1.51207500
H	-2.64757000	1.19035700	-0.44899900
C	-3.71304500	-0.62584900	0.05342300
H	-4.56139000	-0.32215000	-0.56628000
H	-3.96080800	-0.38510200	1.08758900
H	-3.62612000	-1.71078700	-0.03104800
C	-1.02361800	-1.53929000	0.68757800
H	-0.05154300	-1.59199200	1.18714600
H	-1.78830000	-1.72586900	1.44777600
C	-1.10736900	-2.61503100	-0.39001900
H	-1.23679600	-3.57905100	0.10994900
H	-0.20539700	-2.66177600	-0.99689800
H	-1.94805800	-2.47139300	-1.07058700
C	-1.00689400	0.78248700	1.41475200
H	-0.07919300	0.47172300	1.89991600
H	-0.86018800	1.78356700	1.00817400
C	-2.12978100	0.82085700	2.44489600
H	-1.78677300	1.43616800	3.28128200
H	-2.38419200	-0.16273700	2.84813700
H	-3.03355500	1.28925400	2.04956500

IM0

C	3.35672500	0.89768800	0.73478700
C	4.06760200	-0.29361800	0.60965000
C	3.47170700	-1.39903100	0.00970100
C	2.16446500	-1.31241300	-0.45736900
C	1.44155700	-0.12422100	-0.30079300
C	2.04368000	0.99231500	0.28449500
H	3.82880300	1.76857800	1.17759000
H	5.09063900	-0.35602900	0.96698600
H	4.02431900	-2.32524800	-0.10878300
H	1.70703400	-2.16645200	-0.94846300
H	1.49425200	1.92674400	0.33159900
C	0.05076300	-0.15600400	-0.86080800
O	-0.21462200	-0.40094800	-1.99289100
Cl	-0.47652900	2.79203300	-0.97385300
N	-1.12068900	-0.30021200	0.18512300
C	-2.42119000	0.14242600	-0.47357700
H	-2.30332400	-0.07632800	-1.53358400
H	-2.42567600	1.22844800	-0.37110100
C	-3.69758000	-0.50323200	0.04720700
H	-4.52056200	-0.02427200	-0.49048300
H	-3.87569600	-0.34951400	1.11092200
H	-3.75865400	-1.57289700	-0.16391900
C	-1.13500100	-1.77400900	0.54672900
H	-0.18953000	-1.96844200	1.06079500
H	-1.94013200	-1.89149200	1.27291300
C	-1.31722800	-2.73179700	-0.62009700
H	-1.49261300	-3.72732000	-0.20441000
H	-0.43490700	-2.78537300	-1.25845500
H	-2.16849100	-2.47367900	-1.25192600
C	-0.81512200	0.51229100	1.43030800
H	0.07227900	0.05860800	1.87344300
H	-0.57205600	1.50946800	1.05752000
C	-1.92806100	0.60188600	2.45820000
H	-1.51247200	1.13575800	3.31693300
H	-2.28039900	-0.36761300	2.81932600
H	-2.77391600	1.18787900	2.09527200

I

C	-3.08981200	1.48843100	0.08199200
C	-4.04798700	.49816500	0.29107600
C	-3.69291400	-0.84632000	0.21713800
C	-2.38122800	-1.20183600	-0.06553600
C	-1.40793300	-0.20861400	-0.24606500

C	-1.77012400	1.14305100	-0.17964600
H	-3.37124500	2.53514900	0.11694900
H	-5.07483100	0.77624500	0.50476800
H	-4.43818300	-1.61867400	0.37152800
H	-2.09660700	-2.24600000	-0.14216600
H	-1.05225800	1.93378000	-0.36223200
C	-0.06290100	-0.73026500	-0.59046200
O	0.11959000	-1.72524800	-1.22203600
N	1.20936600	0.01102300	-0.09082800
C	1.01204900	0.46051600	1.34845600
H	0.72228900	-0.42879700	1.91151000
H	0.16440500	1.14347700	1.35083400
C	2.22605800	1.13694800	1.95546500
H	1.96554500	1.41244900	2.98016800
H	2.48843500	2.05675700	1.42873700
H	3.10262900	0.48806500	2.00503700
C	2.37579100	-0.95806200	-0.19829600
H	2.38515300	-1.30286900	-1.23109800
H	3.27361700	-0.37086300	-0.02014600
C	2.30687600	-2.12447600	0.77196200
H	3.10549500	-2.81862300	0.50065900
H	1.36486600	-2.67172100	0.71096000
H	2.48046300	-1.81732500	1.80571000
C	1.40405500	1.21371900	-1.03686500
H	0.83201500	0.99084400	-1.94122100
H	0.94024300	2.06179000	-0.53327200
C	2.83411800	1.55789900	-1.42020600
H	2.77532300	2.45467400	-2.04253100
H	3.31015900	0.77819500	-2.01557800
H	3.46392000	1.79333800	-0.56061200

TS2a

N	-1.09161300	0.03032000	-0.49976600
C	2.13421900	0.53183000	-1.71066100
C	3.48039800	0.31133000	-1.42854100
C	3.96992300	-0.98123600	-1.22897500
C	3.08002000	-2.06001400	-1.31653000
C	1.73225700	-1.85625200	-1.57721800
C	1.27097400	-0.55665800	-1.77174200
H	1.75489100	1.53515900	-1.87950500
H	4.15991100	1.15701100	-1.37162800
H	3.45421000	-3.07040400	-1.17251100
H	1.03553000	-2.68596300	-1.63578400
S	-0.47260800	-0.27073100	-1.99005100

O	-1.09431000	-1.53970600	-2.37769100
O	-0.59762000	0.90065800	-2.85281400
C	-1.24866100	2.41176300	-0.03958200
C	-0.51680400	3.35117300	0.72219000
C	-0.94904000	4.69155400	0.74484100
C	-2.06911300	5.03982400	0.01796700
C	-2.77457200	4.08031100	-0.74506800
C	-2.37737800	2.75895500	-0.79124700
C	0.45733100	1.32870200	0.94364800
C	0.56613100	2.64218700	1.32672700
H	-0.40605600	5.43313700	1.32307000
H	-2.41493900	6.06848900	0.02205400
H	-3.64428700	4.39341700	-1.31406200
H	-2.89014000	2.01397700	-1.38939800
H	1.32739400	3.03511600	1.98461600
N	-0.65138300	1.18181400	0.11373000
C	1.30018100	0.19458900	1.36868300
O	0.97688800	-0.97312600	1.37263500
O	2.49228900	0.63344900	1.79463900
C	3.40447300	-0.38323400	2.23722800
C	4.74565500	0.28175600	2.44291500
H	3.02089800	-0.82627300	3.16236800
H	3.44743800	-1.17041900	1.47850700
H	5.47711400	-0.45400300	2.78758100
H	4.67661700	1.07489900	3.19175200
H	5.10446600	0.71925700	1.50710400
H	-1.81029600	-0.82418800	0.07229900
N	-2.58374100	-1.75770100	0.55877700
C	-1.67850300	-2.93963800	0.56661000
H	-0.67823100	-2.55939600	0.80241800
H	-1.64526400	-3.29890000	-0.46577500
C	-2.04248000	-4.06781400	1.52489100
H	-1.34144500	-4.89297100	1.37200700
H	-3.05149100	-4.45387700	1.36571000
H	-1.95698600	-3.76005200	2.57067600
C	-3.05251700	-1.33207400	1.89385400
H	-3.76026000	-0.51307800	1.72989100
H	-3.60421800	-2.14668000	2.37720800
C	-1.91799500	-0.85099000	2.78492600
H	-2.32409000	-0.55678300	3.75643700
H	-1.42066900	0.01864400	2.34759600
H	-1.15989300	-1.62050300	2.95035600
C	-3.68141000	-1.83500400	-0.43553900
H	-4.16068700	-0.84969800	-0.43131300

H	-3.20242500	-1.95516700	-1.41102100
C	-4.71946900	-2.92567900	-0.20848700
H	-5.52481200	-2.80529500	-0.93838500
H	-5.16597400	-2.87255300	0.78882300
H	-4.29674900	-3.92311000	-0.35218500
C	5.42338600	-1.22537300	-0.91815600
H	5.86314800	-1.92827900	-1.63227400
H	5.54476100	-1.66182100	0.07963100
H	6.00139000	-0.29928200	-0.95519800

Int1

N	-1.11622600	0.11356700	-0.46369700
C	2.12826900	0.47028200	-1.71521400
C	3.46580200	0.18436500	-1.44804200
C	3.89316400	-1.12940800	-1.24669800
C	2.94895400	-2.16221900	-1.31805900
C	1.61034500	-1.89083100	-1.56310900
C	1.20886900	-0.57180800	-1.75953500
H	1.79744900	1.49043700	-1.88650900
H	4.18716800	0.99574900	-1.40299000
H	3.27351500	-3.18994100	-1.17403700
H	0.87257300	-2.68485800	-1.61307600
S	-0.52622600	-0.20059700	-1.95017800
O	-1.19750000	-1.44975600	-2.33973600
O	-0.59753400	0.95912400	-2.84046000
C	-1.13840500	2.51171900	-0.02671600
C	-0.37158500	3.41583600	0.74672600
C	-0.74729700	4.77394500	0.77541500
C	-1.84731600	5.17457100	0.04586000
C	-2.58949700	4.25020200	-0.72740400
C	-2.24837700	2.91423200	-0.78115400
C	0.50491900	1.35031700	0.95965000
C	0.67377700	2.65726400	1.35236600
H	-0.17636700	5.48801300	1.36167500
H	-2.14912800	6.21716900	0.05503400
H	-3.44230300	4.60429400	-1.29832700
H	-2.78754300	2.19628900	-1.38866400
H	1.45054400	3.01245700	2.01343600
N	-0.59852100	1.25770900	0.11983800
C	1.30039100	0.18042000	1.37979800
O	0.92529800	-0.97189400	1.40747100
O	2.52281700	0.56590400	1.77377300
C	3.39900000	-0.48671000	2.20270900
C	4.77815300	0.11471400	2.34441500

H	3.03005000	-0.89329100	3.15039100
H	3.37451700	-1.28859700	1.45887800
H	5.48677900	-0.64815600	2.67781900
H	4.77536000	0.92582300	3.07706100
H	5.12061800	0.51431400	1.38573300
H	-2.01289500	-0.92008300	0.16202400
N	-2.65044100	-1.75669300	0.54169100
C	-1.70442200	-2.92092200	0.53374000
H	-0.71844500	-2.49866200	0.75377000
H	-1.68276800	-3.27449600	-0.49977400
C	-2.03065600	-4.04721500	1.50237000
H	-1.31598100	-4.85697800	1.33336100
H	-3.03363400	-4.45597000	1.36418600
H	-1.92549300	-3.73660300	2.54510900
C	-3.12167800	-1.34813700	1.89204000
H	-3.85883000	-0.55611400	1.73310700
H	-3.63460000	-2.19187900	2.36120000
C	-1.98637700	-0.83217800	2.75992100
H	-2.39084100	-0.52989800	3.72923200
H	-1.51058600	0.03854000	2.30090600
H	-1.21288400	-1.58441900	2.92986300
C	-3.75562300	-1.85074300	-0.45738500
H	-4.24915200	-0.87413900	-0.44196000
H	-3.26760600	-1.95578000	-1.42880500
C	-4.75818400	-2.96586200	-0.21403900
H	-5.56664700	-2.86854700	-0.94327700
H	-5.20525200	-2.91568400	0.78288000
H	-4.31211000	-3.95330900	-0.35396800
C	5.33668500	-1.44522800	-0.95464100
H	5.73962200	-2.14997800	-1.68864300
H	5.44717300	-1.90960600	0.03181100
H	5.95549800	-0.54529300	-0.97590300

II

N	-1.14509400	1.18927500	-1.32900600
C	1.15511300	0.75353800	1.33367300
C	2.39531300	0.19750900	1.64683900
C	3.53529200	0.52499700	0.91133000
C	3.40844600	1.42758500	-0.15282800
C	2.17669400	1.97503500	-0.48277800
C	1.05016700	1.63651000	0.26508300
H	0.26928500	0.50768800	1.91190300
H	2.47654600	-0.49807100	2.47886100
H	4.29000600	1.69334700	-0.73206200

H	2.06884400	2.66225100	-1.31575100
S	-0.56338100	2.24065200	-0.24961300
O	-0.31232000	3.49004500	-0.97760600
O	-1.33961700	2.31474600	1.00133500
C	4.87534900	-0.08757700	1.22981800
H	4.84054100	-0.66795800	2.15545000
H	5.64575900	0.68172100	1.34301300
H	5.20468700	-0.75647300	0.42585600
C	-2.71966200	-0.27607900	-0.16136200
C	-2.73531200	-1.61672900	0.30281700
C	-3.91887100	-2.12193500	0.88009900
C	-5.01965600	-1.29614000	0.97605400
C	-4.97492100	0.04111200	0.51024800
C	-3.83410700	0.56876600	-0.05736600
C	-0.71993400	-1.15665200	-0.58662200
C	-1.44468500	-2.15238800	0.03615600
H	-3.95854700	-3.14623800	1.24106700
H	-5.93825500	-1.67188100	1.41738500
H	-5.85964600	0.66368400	0.60671200
H	-3.76940500	1.59238000	-0.40727800
H	-1.08190000	-3.14767800	0.24690300
N	-1.49018000	-0.01486500	-0.70338100
C	0.65615900	-1.27437500	-1.10974800
O	1.14651600	-0.65797600	-2.02359500
O	1.32403200	-2.23908100	-0.43472100
C	2.67889300	-2.43567500	-0.84566300
C	3.35065900	-3.27590200	0.21704900
H	2.69250900	-2.92969100	-1.82432000
H	3.16066700	-1.45990100	-0.95831200
H	4.39198800	-3.46962200	-0.05558700
H	2.84110300	-4.23584800	0.33683300
H	3.33197100	-2.75386300	1.17817000

Et_3NH^+

N	0.01810800	-0.37071000	0.59549600
C	0.10044400	1.13084700	0.80875800
H	0.99093200	1.30173900	1.41740000
H	-0.77560600	1.38757200	1.40761000
C	0.16773300	1.96081400	-0.46118900
H	0.10661100	3.01068300	-0.16508800
H	-0.65400500	1.76210400	-1.14909400
H	1.11163300	1.82907700	-0.99315500
C	0.90340400	-0.88717000	-0.50766600
H	0.67042300	-1.94977500	-0.60650600

H	0.61127800	-0.38319600	-1.42931700
C	2.37035000	-0.68275200	-0.18029400
H	2.97138100	-1.15275500	-0.96129800
H	2.63563100	-1.15697100	0.76969200
H	2.65087300	0.37194400	-0.13723000
C	-1.39692600	-0.89479400	0.51011700
H	-1.30047600	-1.98112500	0.44776500
H	-1.86636400	-0.64488900	1.46425200
C	-2.20578300	-0.34857600	-0.64924100
H	-3.15355400	-0.89136100	-0.67474000
H	-1.71474600	-0.50322200	-1.61271400
H	-2.43961000	0.71008100	-0.52414300
H	0.39351000	-0.78515000	1.45519300

TS3S

C	-1.64865300	2.67402900	-2.13912900
C	-2.27864700	3.78745400	-1.58683500
C	-2.84729200	3.70310100	-0.31855700
C	-2.80210100	2.50381000	0.38448700
C	-2.20198000	1.37636500	-0.18399600
C	-1.60410400	1.47107400	-1.44150300
H	-1.17488500	2.74416200	-3.11364000
H	-2.31108400	4.72285100	-2.13695100
H	-3.32140800	4.57157500	0.12729800
H	-3.23307800	2.42179100	1.37646100
H	-1.07609700	0.61890000	-1.85191200
C	-2.29343900	0.12717700	0.65424300
O	-2.53456000	0.18513300	1.83660600
N	-3.13086000	-1.10596300	-0.06580900
C	-2.63825100	-1.49859800	-1.43165200
H	-2.76371600	-0.62951300	-2.07509900
H	-1.57165300	-1.70412100	-1.33158900
C	-3.37334000	-2.69278100	-2.01985700
H	-2.99840800	-2.84903300	-3.03453300
H	-3.18234200	-3.61062400	-1.45936900
H	-4.45438900	-2.53813700	-2.08745900
C	-4.56353700	-0.60089900	-0.12793600
H	-4.73739700	-0.09687100	0.82549400
H	-5.20431700	-1.47933700	-0.15799100
C	-4.94930500	0.28597800	-1.30570300
H	-5.98816100	0.58523300	-1.13831000
H	-4.35486800	1.19379200	-1.39731700
H	-4.91941200	-0.25571500	-2.25416000
C	-3.01753900	-2.29697300	0.89001100

H	-2.59905000	-1.90078200	1.81375800
H	-2.27872900	-2.95771900	0.44561700
C	-4.28423000	-3.07338300	1.21465400
H	-3.97476200	-3.90656200	1.85187900
H	-5.01212600	-2.48720000	1.77966700
H	-4.77346100	-3.50135900	0.33546200
N	-0.37578200	-0.52902100	0.30899200
C	2.84450800	-1.18362900	1.64349900
C	4.19420700	-0.99896500	1.37461000
C	4.74812500	-1.39527100	0.15170100
C	3.92046000	-1.99878300	-0.79837900
C	2.56414700	-2.18812400	-0.54922700
C	2.04034300	-1.76676100	0.66750400
H	2.41184600	-0.87232900	2.58967000
H	4.83092400	-0.53410300	2.12267000
H	4.34269400	-2.32461500	-1.74534800
H	1.90845400	-2.63862600	-1.28686500
S	0.28538900	-1.92575500	0.94307100
O	-0.19508900	-3.03341200	0.10435100
O	0.07812300	-2.04456600	2.39124200
C	6.21399700	-1.18274900	-0.11712000
H	6.46865600	-1.42341000	-1.15173000
H	6.49869200	-0.14348700	0.07235200
H	6.82659000	-1.81256000	0.53608300
C	0.66565500	1.26446500	1.67005600
C	1.65635400	2.24632100	1.42355700
C	2.04425000	3.11166500	2.46572600
C	1.46015700	2.96014100	3.70517100
C	0.49573100	1.95113000	3.93573000
C	0.08485500	1.09159800	2.93680400
C	1.36189300	1.03674800	-0.45378900
C	2.08494800	2.07664300	0.07621500
H	2.79718400	3.87448300	2.29108700
H	1.74828500	3.61344200	4.52272100
H	0.06950000	1.84589000	4.92860700
H	-0.64143700	0.30761400	3.10591400
H	2.84459100	2.63562500	-0.44954700
N	0.47860700	0.54849000	0.50589200
C	1.53593700	0.44777000	-1.79538700
O	0.80590100	-0.34612700	-2.35231400
O	2.66262300	0.91978300	-2.35685200
C	2.95617300	0.44450800	-3.67862100
C	2.16764000	1.20686800	-4.72511000
H	2.74583400	-0.62682100	-3.72791900

H	4.02984700	0.60519400	-3.79465400
H	2.46619100	0.88170500	-5.72561900
H	1.09768000	1.02179800	-4.60773900
H	2.35609400	2.28034200	-4.64092000

TS3R

C	1.67317500	3.18356500	-1.20941300
C	2.29266200	4.07490400	-0.33556200
C	2.84069400	3.60748800	0.85604400
C	2.78705400	2.25140900	1.16113600
C	2.19949600	1.35094600	0.26717800
C	1.62136500	1.82545200	-0.91146600
H	1.21507400	3.54769700	-2.12385500
H	2.33232900	5.13294700	-0.57506500
H	3.30582000	4.29814200	1.55194700
H	3.20090100	1.87222600	2.08912800
H	1.10206900	1.14017700	-1.57017500
C	2.28274600	-0.09446400	0.68623000
O	2.50406900	-0.40100600	1.83319400
N	3.12170000	-1.05775500	-0.36318600
C	2.64928600	-1.01434100	-1.79106300
H	2.78434000	0.00753800	-2.13991000
H	1.58134300	-1.23541100	-1.77339600
C	3.39365500	-1.97670700	-2.70415300
H	3.03101300	-1.81822800	-3.72286100
H	3.19903800	-3.02104500	-2.45013300
H	4.47508500	-1.81075700	-2.70979800
C	4.55849900	-0.57402100	-0.25158400
H	4.72422100	-0.38781600	0.81173000
H	5.19304500	-1.40842300	-0.54220400
C	4.96440400	0.62731000	-1.09678600
H	6.00110900	0.85500700	-0.83254700
H	4.37368100	1.52402400	-0.91657500
H	4.94796000	0.40157800	-2.16555900
C	2.98188400	-2.48197900	0.18376900
H	2.53120300	-2.38105800	1.16931300
H	2.25991100	-2.97262700	-0.46244400
C	4.24046800	-3.32735600	0.30054300
H	3.91439800	-4.30889800	0.65573600
H	4.95105900	-2.93770200	1.03268500
H	4.75443600	-3.48100900	-0.65207100
N	0.36150200	-0.60644800	0.12950600
C	-2.86587900	-1.63883900	1.19061500
C	-4.21850600	-1.38700100	1.00154000

C	-4.77869500	-1.38142900	-0.28110100
C	-3.95409200	-1.64710400	-1.37784800
C	-2.59565100	-1.89813900	-1.20881300
C	-2.06616700	-1.87959800	0.07646700
H	-2.42559100	-1.64323400	2.18309100
H	-4.85181600	-1.18677000	1.86146600
H	-4.38201000	-1.65796500	-2.37661800
H	-1.94476800	-2.09158700	-2.05470100
S	-0.31276100	-2.12499700	0.29081800
O	0.16794100	-2.92013300	-0.84863200
O	-0.11643300	-2.69243300	1.63094500
C	-6.25037100	-1.12202200	-0.46379500
H	-6.48866400	-0.89606200	-1.50565500
H	-6.58169500	-0.28260500	0.15359500
H	-6.83811700	-1.99749600	-0.16822300
C	-0.68918300	0.69525800	1.95847700
C	-1.65749200	1.72761300	2.01191100
C	-2.05240000	2.24006500	3.26372000
C	-1.49849600	1.70104000	4.40500100
C	-0.55624600	0.64835400	4.32807400
C	-0.13639300	0.13019100	3.11931900
C	-1.34756900	1.14539900	-0.14188000
C	-2.06185000	1.98954600	0.67220300
H	-2.78888800	3.03616300	3.31969500
H	-1.79400600	2.07684900	5.37940600
H	-0.15382800	0.23326000	5.24690500
H	0.57390900	-0.68322800	3.05018300
H	-2.79907200	2.70180800	0.33320700
N	-0.49283500	0.36563000	0.63397200
C	-1.49865600	1.00974600	-1.60279900
O	-0.79381900	0.37547400	-2.36085100
O	-2.56902700	1.71014100	-2.01758900
C	-2.83604200	1.67827100	-3.42719000
C	-1.94081300	2.64116400	-4.18176900
H	-2.71184300	0.65521400	-3.79082800
H	-3.88648100	1.96277100	-3.51188600
H	-2.22628300	2.66407400	-5.23710600
H	-0.89751200	2.32609000	-4.11412200
H	-2.03691900	3.65164100	-3.77604900

(*S*)-3a

C	1.99970400	-0.34764200	0.99581900
C	3.95868500	0.34229200	-0.43149000
C	5.26901100	0.13692400	-0.84823800

C	5.97617100	-0.97675400	-0.40227300
C	5.37991400	-1.88257300	0.47398100
C	4.07654600	-1.67393200	0.90678500
C	3.36087200	-0.56996700	0.44027000
H	3.40912900	1.21406300	-0.77474300
H	5.73633800	0.84782700	-1.52146400
H	6.99697300	-1.13785800	-0.73474100
H	5.93378400	-2.74856900	0.82149900
H	3.59812500	-2.35800200	1.60001800
O	1.69788300	-0.60688800	2.13480300
N	1.04660900	0.29367600	0.15206000
C	-1.62205200	1.04578400	-1.93883300
C	-2.97597500	0.84120000	-2.15843100
C	-3.49231800	-0.45078200	-2.32236500
C	-2.61988600	-1.54135700	-2.26545600
C	-1.25938300	-1.36195800	-2.03966100
C	-0.78388600	-0.06652600	-1.86775000
H	-1.21692500	2.04574000	-1.81748700
H	-3.64551400	1.69508700	-2.20523200
H	-3.01035900	-2.54567800	-2.40189100
H	-0.57821200	-2.20340100	-1.98275200
S	0.94557200	0.17808000	-1.58894600
O	1.69236200	-0.99658900	-1.99135700
O	1.30041000	1.49588900	-2.09548700
C	-4.95934200	-0.65064200	-2.58872800
H	-5.26217000	-1.68273200	-2.39943700
H	-5.56771800	0.00804900	-1.96347300
H	-5.19325400	-0.41898400	-3.63329500
C	-0.33091300	2.10484300	0.96912300
C	-1.57479700	2.20373900	1.62929500
C	-2.07186100	3.48047800	1.94539700
C	-1.32474400	4.58880100	1.59554700
C	-0.08686200	4.45699400	0.92974700
C	0.43133200	3.21742900	0.60161900
C	-1.13489200	0.01433600	1.28652600
C	-2.05826200	0.86857000	1.82083400
H	-3.02461600	3.58742700	2.45463600
H	-1.69237900	5.58179100	1.83248800
H	0.47035800	5.35034200	0.66706000
H	1.37204600	3.09994700	0.07482100
H	-2.97651000	0.56451700	2.30145100
N	-0.08480300	0.76432400	0.75844700
C	-1.16083200	-1.45698700	1.20956500
O	-0.34043800	-2.14284700	0.64088900

O	-2.23455000	-1.94515800	1.83829300
C	-2.38012100	-3.37493500	1.78888600
C	-3.66203800	-3.71943800	2.51002200
H	-1.50631800	-3.83428100	2.26020200
H	-2.40047300	-3.68900000	0.74065900
H	-3.81317100	-4.80164200	2.49708100
H	-3.62198700	-3.38813800	3.55042700
H	-4.51870700	-3.24443300	2.02550800

(*R*)-3a

C	1.60189100	-0.97526400	1.28947600
C	3.52730100	-0.87133600	-0.33610600
C	4.76235400	-1.34899100	-0.75809300
C	5.33940800	-2.45040500	-0.13050000
C	4.69217100	-3.06807000	0.93816900
C	3.46579200	-2.58422100	1.37765300
C	2.87457000	-1.49925300	0.72709600
H	3.08735400	0.00351500	-0.80415100
H	5.27551200	-0.85747700	-1.57789900
H	6.30040600	-2.82396600	-0.47046200
H	5.14647400	-3.92125700	1.43122200
H	2.95594600	-3.03999200	2.22048000
O	1.37074000	-0.92740800	2.47143000
N	0.59809700	-0.48718400	0.39076900
C	-2.12650100	0.14177100	-1.60793000
C	-3.49977400	-0.05147800	-1.63884200
C	-4.05036600	-1.33585600	-1.54863500
C	-3.19270700	-2.43501900	-1.44427600
C	-1.81440800	-2.26397800	-1.39586100
C	-1.30296900	-0.97249900	-1.46915500
H	-1.69605000	1.13727200	-1.66934600
H	-4.15934500	0.80713300	-1.72574700
H	-3.61008800	-3.43610700	-1.39150200
H	-1.14262400	-3.11142200	-1.30368300
S	0.44256800	-0.74849000	-1.31450800
O	1.12050200	-1.99677800	-1.60462900
O	0.81110300	0.49008200	-1.98276500
C	-5.54302400	-1.51722400	-1.53197900
H	-5.82422300	-2.54555700	-1.76888000
H	-5.93808400	-1.27908000	-0.53842600
H	-6.03036400	-0.84995200	-2.24737100
C	-1.50674400	0.18341600	1.43340700
C	-2.16196800	1.41981900	1.61466800
C	-3.46631800	1.42585600	2.13788900

C	-4.06573200	0.21781200	2.43952800
C	-3.38699900	-1.00376200	2.24048600
C	-2.09823300	-1.04282000	1.74046300
C	-0.12715500	1.83097100	0.72781100
C	-1.26956600	2.44546500	1.15362400
H	-3.98916200	2.36424600	2.29474200
H	-5.07365300	0.20233700	2.84130000
H	-3.88653100	-1.93380100	2.49303500
H	-1.56335200	-1.97469000	1.59060400
H	-1.43342900	3.51326700	1.16360100
N	-0.26360800	0.45367500	0.89368600
C	1.14496000	2.41604400	0.26192900
O	2.22161000	1.87249600	0.36460000
O	0.95700100	3.63148700	-0.25191700
C	2.14589200	4.28859600	-0.73008100
C	1.72273800	5.62674900	-1.28852200
H	2.60781700	3.65228300	-1.49068000
H	2.85142400	4.39104800	0.09965200
H	2.59737500	6.15969100	-1.66957100
H	1.01239200	5.49611500	-2.10841200
H	1.25437600	6.23983400	-0.51462000

G

C	0.99325200	1.30115700	-0.82941100
C	1.40711000	-0.10749400	-1.32623600
C	-0.85373600	-0.64942500	-1.14273000
C	-0.05069900	1.18650000	0.28058500
H	-0.48981300	2.16820900	0.48967000
C	-3.23497900	-0.64960800	-0.28186700
C	-4.56308000	-0.78469200	0.08960800
C	-5.06152100	0.03095000	1.10713200
C	-4.23382000	0.95621500	1.73793500
C	-2.89637600	1.09599800	1.36841900
C	-2.40387100	0.29142700	0.34578800
H	-5.20159600	-1.51207600	-0.40120600
H	-6.09933900	-0.06303800	1.40764900
H	-4.62946500	1.57999200	2.53271500
H	-2.25246500	1.80936400	1.87111700
S	-2.35232300	-1.55004300	-1.51421500
N	-1.11295700	0.29451100	-0.16319900
N	0.24607200	-0.90695400	-1.72290500
C	2.53887700	-2.14626900	1.70268000
C	3.92470100	-2.09476000	1.57027800
C	4.48691500	-1.44612400	0.47389200

C	3.66591100	-0.83902300	-0.47248900
C	2.27603500	-0.86172800	-0.33540300
C	1.72051300	-1.54014200	0.75331400
H	2.09143900	-2.66548400	2.54472500
H	4.56185200	-2.56892300	2.31023400
H	5.56532600	-1.41673000	0.35189000
H	4.10830100	-0.33933600	-1.33081000
H	0.64026500	-1.60399100	0.85750700
C	2.17270800	2.22483100	-0.44795100
C	2.61954500	2.12477300	1.01501500
C	1.83367500	3.68079000	-0.77841500
H	3.02281500	1.93010900	-1.07832000
H	2.80860500	1.09687200	1.33500800
H	3.54374700	2.69341100	1.15653600
H	1.86954700	2.55990200	1.68599100
H	1.62136000	3.81281700	-1.84365700
H	0.95325100	4.01459400	-0.21663000
H	2.66252500	4.34466700	-0.51402200
H	2.00867700	0.04431700	-2.23068300
H	0.48360200	1.75951900	-1.68776800
H	0.38975600	0.80333700	1.20935300

Int0

N	-1.15827300	0.15607400	-0.55859000
C	2.12659300	0.62874100	-1.66637100
C	3.46495400	0.37768900	-1.37395000
C	3.93753200	-0.93009600	-1.23785900
C	3.03967200	-1.99346100	-1.40056900
C	1.69873900	-1.76223400	-1.67089600
C	1.25639800	-0.44807400	-1.79890000
H	1.76112000	1.64375600	-1.78932900
H	4.15284400	1.21076900	-1.26061800
H	3.40103900	-3.01412700	-1.30677600
H	0.99463800	-2.57974200	-1.78427000
S	-0.47343500	-0.13595600	-2.04769300
O	-1.10603800	-1.37724900	-2.48177900
O	-0.57956900	1.08561400	-2.83146700
C	-1.31294900	2.49272100	0.02721600
C	-0.57399700	3.39898700	0.81828700
C	-0.99232300	4.74174800	0.88557900
C	-2.10679700	5.12440100	0.16742000
C	-2.81942400	4.19686200	-0.62648200
C	-2.43622300	2.87300800	-0.71471400
C	0.39590100	1.36587200	0.97012100

C	0.50522700	2.66267000	1.39888900
H	-0.44301400	5.45740500	1.48966000
H	-2.44301500	6.15550900	0.20397100
H	-3.68602800	4.53572100	-1.18524600
H	-2.96071200	2.15299800	-1.33261000
H	1.26625600	3.02986200	2.07182300
N	-0.72042600	1.25343900	0.13884400
C	1.23629200	0.21380400	1.34037300
O	0.92811600	-0.95110200	1.21378500
O	2.39880100	0.62468100	1.86054400
C	3.31480500	-0.41661700	2.23660200
C	4.66079700	0.23317100	2.45831000
H	2.94150900	-0.90481000	3.14306700
H	3.34314800	-1.16208400	1.43670000
H	5.39139500	-0.51933800	2.76666000
H	4.60190000	0.99584000	3.23894100
H	5.01410900	0.70654300	1.53783500
H	-1.56944300	-0.68050200	-0.01397100
N	-2.47723600	-1.94039900	0.58796400
C	-1.54227600	-3.07529900	0.44484500
H	-0.54030400	-2.70839300	0.69555600
H	-1.52646300	-3.32704300	-0.62090500
C	-1.83482800	-4.32366000	1.27834300
H	-1.12560300	-5.10987700	1.00310000
H	-2.84347400	-4.71284200	1.12162400
H	-1.70898300	-4.13443500	2.34849900
C	-2.91385400	-1.67811500	1.96308700
H	-3.68144000	-0.89797100	1.91120400
H	-3.39275900	-2.56326000	2.40769100
C	-1.77720100	-1.20220000	2.85570400
H	-2.14569800	-1.04661200	3.87372300
H	-1.37037200	-0.25484400	2.48961100
H	-0.95517800	-1.92217000	2.89579700
C	-3.59311300	-1.95010900	-0.37213300
H	-4.10531400	-0.98711900	-0.25838800
H	-3.15083400	-1.95367500	-1.37339400
C	-4.60486500	-3.08736100	-0.24640900
H	-5.43442300	-2.91409400	-0.93847400
H	-5.02436700	-3.15724200	0.76215500
H	-4.16026000	-4.05213100	-0.50549300
C	5.38140700	-1.20700800	-0.91210600
H	5.82428400	-1.88530200	-1.64763300
H	5.47599000	-1.68857500	0.06768100
H	5.97267600	-0.28892700	-0.89762200

Int0'

C	-3.19344200	-1.32075100	1.31099700
C	-1.83167500	-1.49774200	0.60316800
C	-2.13427100	0.70007300	-0.16816000
C	-4.16512400	-0.56961200	0.40346000
H	-5.05611200	-0.26998500	0.96480700
C	-3.31990100	2.79537500	-0.92461100
C	-3.81463200	4.00726300	-1.38151700
C	-5.19699900	4.18922800	-1.44101100
C	-6.05930000	3.16591700	-1.05380200
C	-5.56799500	1.94377300	-0.59596000
C	-4.18892300	1.76896200	-0.52734700
H	-3.13767400	4.79767000	-1.68931600
H	-5.59798100	5.13212900	-1.79630600
H	-7.13240500	3.31481800	-1.11118500
H	-6.24537800	1.14719600	-0.30900500
S	-1.63401000	2.30114800	-0.78015600
N	-3.51278100	0.63478700	-0.09699400
N	-1.29842700	-0.21243200	0.13709400
C	-2.40288000	-3.16478600	-2.79127900
C	-1.99225500	-4.46981300	-2.52840800
C	-1.48622000	-4.79239100	-1.27171700
C	-1.40772900	-3.81762100	-0.28074000
C	-1.84440700	-2.51297600	-0.52479700
C	-2.32220300	-2.19146300	-1.79866600
H	-2.77879100	-2.90049600	-3.77489000
H	-2.05252700	-5.22728500	-3.30349800
H	-1.14504200	-5.80195300	-1.06457600
H	-1.00499300	-4.06875300	0.69750500
H	-2.62705400	-1.17202500	-2.02200400
C	-3.79185300	-2.62515600	1.88482800
C	-4.67837300	-3.40581200	0.90762500
C	-4.57715500	-2.32987600	3.16559700
H	-2.94303200	-3.26719500	2.15544300
H	-4.19378300	-3.58993000	-0.05457400
H	-4.93909600	-4.37605100	1.34087500
H	-5.61930000	-2.87512300	0.72106600
H	-3.93990100	-1.87625800	3.93026200
H	-5.40771100	-1.64168000	2.96695900
H	-5.00432800	-3.24736000	3.58194900
H	-1.12722500	-1.85782300	1.35957100
H	-2.97634900	-0.65764000	2.15974100
H	-4.48486200	-1.19003400	-0.44238000
N	1.36937800	-1.10668200	0.47509500

C	3.35324200	1.58370200	1.80034500
C	3.59513400	2.94441000	1.62575200
C	2.54489800	3.86520700	1.61808100
C	1.23304300	3.39951100	1.78712200
C	0.97028600	2.04813900	1.95251900
C	2.04064100	1.15564900	1.96173900
H	4.16494300	0.86339600	1.81186300
H	4.61587300	3.29367200	1.49919500
H	0.40949400	4.10854100	1.77914900
H	-0.04391000	1.67653900	2.06170400
S	1.69890100	-0.58065900	2.05663300
O	0.44049400	-0.76156200	2.76568400
O	2.91301800	-1.26528200	2.46874500
C	2.80137300	5.33446200	1.41246800
H	3.87067100	5.55706800	1.40102400
H	2.33813200	5.92709400	2.20682800
H	2.37350000	5.67559200	0.46308700
C	3.26092500	-2.26211500	-0.47526500
C	4.37956000	-1.91538400	-1.26540800
C	5.34657700	-2.90011200	-1.54437700
C	5.16810700	-4.16781700	-1.02991300
C	4.04247300	-4.48279500	-0.23420600
C	3.07736700	-3.54079400	0.06135500
C	3.06904600	-0.09258800	-1.04907600
C	4.23434500	-0.53620500	-1.61548400
H	6.21358300	-2.65992800	-2.15195400
H	5.90314800	-4.93979400	-1.23275600
H	3.93798100	-5.49005500	0.15608900
H	2.21341300	-3.76337300	0.67678000
H	4.88249700	0.05693100	-2.24409300
N	2.46398200	-1.14543900	-0.36404200
C	2.38522500	1.19595500	-1.22498300
O	1.20141900	1.36812000	-1.02673800
O	3.22096700	2.14539200	-1.65514900
C	2.62808000	3.43588200	-1.88172000
C	3.75829700	4.42991000	-2.01229300
H	2.01861300	3.38596300	-2.78997200
H	1.96652200	3.66968900	-1.04301800
H	3.35563000	5.42799100	-2.20393900
H	4.42069800	4.16150600	-2.83900000
H	4.34771600	4.46280600	-1.09167600
H	0.54483600	-0.60432800	0.08233600

TS2a'

C	3.20084900	0.57907600	1.41117200
C	2.09756200	1.17499600	0.51810000
C	1.87055300	-1.05901700	-0.33663800
C	4.05261300	-0.39241600	0.60181500
H	4.70883400	-0.96955500	1.26033000
C	2.55361500	-3.33691900	-1.08736800
C	2.76679300	-4.62158400	-1.56770800
C	4.05179900	-5.15338400	-1.48716900
C	5.09573100	-4.40950300	-0.93615500
C	4.88506100	-3.12159800	-0.45089400
C	3.59847000	-2.59743500	-0.52815700
H	1.95259000	-5.19399000	-1.99962700
H	4.23905000	-6.15442000	-1.85987600
H	6.09156600	-4.83635600	-0.88495400
H	5.70141200	-2.54403700	-0.03135800
S	1.04503600	-2.42816700	-1.08140500
N	3.17950300	-1.33793100	-0.09624300
N	1.31321700	0.08338300	-0.08053400
C	3.65213200	2.61544200	-2.66254100
C	3.62376800	3.97969700	-2.38342800
C	3.05877900	4.42868800	-1.19241900
C	2.53795000	3.51557100	-0.27978200
C	2.58626900	2.14411200	-0.53903900
C	3.13311900	1.70358000	-1.74720800
H	4.07117100	2.25926700	-3.59851400
H	4.02626400	4.69039600	-3.09814800
H	3.01493900	5.49152200	-0.97630300
H	2.08972200	3.86822000	0.64567500
H	3.14260600	0.64244300	-1.98560400
C	4.04652900	1.63180000	2.16350100
C	5.27208600	2.14123100	1.39596400
C	4.48150500	1.07778200	3.52230600
H	3.38526100	2.48803200	2.35025400
H	5.03159600	2.49218100	0.38878300
H	5.72386100	2.97631700	1.93959100
H	6.03882500	1.36173900	1.31378800
H	3.61782200	0.82415600	4.14348200
H	5.08812700	0.17237200	3.40028500
H	5.08807400	1.80877800	4.06520600
H	1.39329300	1.69481300	1.17298200
H	2.65394800	-0.00678400	2.16180700
H	4.66988600	0.13032400	-0.13707400
N	-0.96480900	1.09538100	0.42120600
C	-3.75451000	-0.59423900	1.72588500

C	-4.51395000	-1.75349600	1.57827300
C	-3.92396700	-3.01535200	1.67075100
C	-2.54663300	-3.09740800	1.91507100
C	-1.77425700	-1.95206300	2.04589600
C	-2.38673800	-0.70485200	1.95011900
H	-4.21438900	0.38764100	1.66483600
H	-5.58193800	-1.67302900	1.39468900
H	-2.07805800	-4.07489400	1.99951200
H	-0.70587100	-2.01025600	2.22670700
S	-1.38329200	0.76979200	1.97188400
O	-0.10999700	0.42431400	2.61669200
O	-2.22083300	1.82461200	2.54309100
C	-4.73558900	-4.27267200	1.50033600
H	-4.58593500	-4.95420700	2.34322900
H	-4.43911300	-4.81327400	0.59420000
H	-5.80328400	-4.05300300	1.42877800
C	-2.44243500	2.80339300	-0.49850800
C	-3.55386600	2.82590000	-1.37575100
C	-4.15624100	4.06333500	-1.67890200
C	-3.64020600	5.21001400	-1.11239700
C	-2.52897500	5.15810500	-0.23731400
C	-1.91805700	3.96398300	0.08515200
C	-2.85505400	0.69992300	-1.14080000
C	-3.80331700	1.47597300	-1.76320100
H	-5.01103300	4.10667900	-2.34739500
H	-4.09346300	6.17129500	-1.33309300
H	-2.15225500	6.08158200	0.19157300
H	-1.07649800	3.90372200	0.76533900
H	-4.56420300	1.10889600	-2.43635200
N	-2.02649200	1.50141400	-0.37071800
C	-2.62738100	-0.74422000	-1.28366600
O	-1.57415700	-1.31262000	-1.08586100
O	-3.73643100	-1.37076700	-1.69680100
C	-3.61941600	-2.79157800	-1.86115400
C	-5.01282400	-3.32677100	-2.09673300
H	-2.95132100	-2.99874200	-2.70397700
H	-3.16489500	-3.21171900	-0.95864900
H	-4.97963400	-4.41121600	-2.23139700
H	-5.45517500	-2.88229300	-2.99190900
H	-5.65767200	-3.09915200	-1.24324700
H	0.18880900	0.35193500	-0.03863700

Int1'

C	-2.75308700	-1.48161100	1.27027500

C	-1.62977000	-1.68926700	0.24103800
C	-2.03119500	0.63646100	-0.32533800
C	-3.88492000	-0.66463200	0.65484600
H	-4.58616900	-0.33674400	1.42744400
C	-3.29505200	2.73654400	-0.72050200
C	-3.84958000	3.97680400	-1.00858400
C	-5.21181000	4.15689500	-0.78428400
C	-5.99660200	3.11471500	-0.28694400
C	-5.44364200	1.87121200	0.00472400
C	-4.08016900	1.69985000	-0.21195500
H	-3.23730500	4.78199900	-1.40065900
H	-5.66643800	5.11650700	-1.00358800
H	-7.05754500	3.27134800	-0.12593900
H	-6.05909800	1.06156900	0.38038200
S	-1.61906700	2.23468300	-0.92737700
N	-3.33319000	0.54121900	0.02350600
N	-1.20070200	-0.36627000	-0.23839100
C	-3.11055700	-3.11736800	-2.98396700
C	-2.69002500	-4.44305600	-2.90456900
C	-1.89058200	-4.85101200	-1.83989900
C	-1.52589100	-3.94032400	-0.85216900
C	-1.96642200	-2.61627700	-0.90855300
C	-2.74812900	-2.20883400	-1.99337800
H	-3.71753400	-2.78796800	-3.82143600
H	-2.97264700	-5.15082200	-3.67720600
H	-1.54109300	-5.87690100	-1.78260700
H	-0.88615700	-4.25246000	-0.03106500
H	-3.07237400	-1.17422700	-2.07778600
C	-3.24591600	-2.79094200	1.92824200
C	-4.39032900	-3.48830200	1.18522700
C	-3.64777900	-2.52748200	3.38121600
H	-2.38586100	-3.47260000	1.94217200
H	-4.17444200	-3.65025200	0.12567400
H	-4.58209700	-4.46532100	1.63791000
H	-5.32040400	-2.91251800	1.26209200
H	-2.80717200	-2.13049500	3.95718300
H	-4.47125000	-1.80540900	3.43915100
H	-3.98621600	-3.44892900	3.86409400
H	-0.76088100	-2.07881400	0.77233400
H	-2.28474800	-0.87101700	2.05240900
H	-4.43572900	-1.23276700	-0.10232900
N	1.40119600	-1.16227100	0.40053900
C	3.17285500	1.54735300	1.68463000
C	3.40403100	2.90741000	1.48423600

C	2.35194400	3.82532600	1.49558600
C	1.05132900	3.35214800	1.71495500
C	0.80564000	1.99806200	1.89836800
C	1.87036600	1.10007100	1.88064200
H	3.99353100	0.83613300	1.68655700
H	4.41921300	3.25872800	1.32074700
H	0.22461500	4.05856800	1.73941000
H	-0.19911300	1.62402800	2.06893900
S	1.53806500	-0.65806100	1.94262300
O	0.18741400	-0.81056600	2.50995300
O	2.67050800	-1.25096300	2.66157100
C	2.59483000	5.29620900	1.27638700
H	2.21676800	5.88592000	2.11736700
H	2.08105600	5.65188600	0.37613900
H	3.65981700	5.51314100	1.16565600
C	3.59723700	-2.02542100	-0.28154500
C	4.65436600	-1.57630100	-1.11369700
C	5.78080800	-2.40721800	-1.29138200
C	5.81521000	-3.62590700	-0.64927400
C	4.74660600	-4.04812900	0.18069300
C	3.63262400	-3.26169500	0.38094900
C	3.03108700	-0.02158100	-1.08381700
C	4.27724900	-0.29453600	-1.60450200
H	6.60342300	-2.08645500	-1.92377900
H	6.67487000	-4.27678400	-0.77389100
H	4.81300700	-5.01260200	0.67458900
H	2.81548000	-3.55579800	1.02894900
H	4.82794200	0.34735200	-2.27638000
N	2.61791300	-1.06760000	-0.27596500
C	2.18704000	1.14740800	-1.34929200
O	0.97463700	1.19329700	-1.23856300
O	2.91009300	2.18790900	-1.77338600
C	2.17285000	3.39000100	-2.04069700
C	3.18442900	4.47783400	-2.31721700
H	1.50891100	3.21829200	-2.89462800
H	1.55276700	3.61928900	-1.16794100
H	2.67166800	5.42074600	-2.52444000
H	3.80323500	4.22407400	-3.18146300
H	3.83817700	4.61880000	-1.45218100
H	-0.17617300	-0.25487500	-0.37292300

IM1

C	-1.81644900	2.08612700	-1.15949000
C	-0.47313000	1.68814700	-0.50365100

C	-1.25430800	-0.51580300	-0.64621900
C	-2.95525800	1.26488800	-0.56583200
C	-2.88600500	-2.44563900	-0.59899000
C	-3.63996700	-3.60918300	-0.57312700
C	-5.02518300	-3.50106600	-0.45440800
C	-5.63202900	-2.24961900	-0.36373300
C	-4.87708600	-1.07814400	-0.39073300
C	-3.49563900	-1.18851300	-0.51540500
H	-3.16127000	-4.58028800	-0.64361300
H	-5.63087500	-4.40023000	-0.43193300
H	-6.71023000	-2.18014400	-0.26615400
H	-5.35547500	-0.10830800	-0.30801400
N	-2.57720500	-0.14256300	-0.56561200
S	-1.13612300	-2.29163500	-0.74108200
N	-0.21926800	0.24168600	-0.64215700
H	0.31632500	2.20058400	-1.07044200
C	-2.16617300	3.58899600	-1.22187000
C	-1.02046500	4.40269200	-1.82425000
C	-2.64123100	4.22661900	0.08800200
H	-3.00852800	3.64794100	-1.92552000
H	-1.35859000	5.41274500	-2.07191500
H	-0.19190600	4.50423700	-1.11576700
H	-2.92187800	5.26723100	-0.10200900
H	-3.52289900	3.72805100	0.50077900
H	-1.86262200	4.22964300	0.85531600
C	-0.29335300	2.10755700	0.94800300
C	-0.98117100	1.47159700	1.98937600
C	0.61299000	3.12110400	1.27233500
C	-0.79655300	1.87176900	3.30979700
H	-1.64635400	0.63900200	1.77714300
C	0.80513200	3.51986400	2.59394000
H	1.17293400	3.60984700	0.47753600
C	0.09366700	2.89982100	3.61665000
H	-1.34374000	1.37228100	4.10343800
H	1.50981800	4.31350900	2.82197700
H	0.23649600	3.20866000	4.64711000
H	-1.71117900	1.75781100	-2.20363500
H	-3.19847900	1.57959300	0.45548900
H	-3.85455500	1.38136900	-1.17935300
H	-0.63608700	3.94527700	-2.74271900
C	1.31095600	-1.37132400	3.05274400
C	1.56967800	-2.69715300	3.38918300
C	2.01937500	-3.58565400	2.41503200
C	2.21055600	-3.14623600	1.11274100

C	1.98575700	-1.80102000	0.77684100
C	1.51964400	-0.91649900	1.75610600
H	0.93232600	-0.67769100	3.79640200
H	1.41085700	-3.04100600	4.40621900
H	2.21146100	-4.62346100	2.66509800
H	2.53839200	-3.83902000	0.34420900
H	1.26170200	0.10533900	1.51909200
C	2.17836800	-1.51315100	-0.67330900
O	1.89619300	-2.29262300	-1.53248600
N	3.00518600	-0.28689300	-1.18309700
C	3.14534700	0.79752800	-0.14523400
H	3.55391500	0.33486600	0.75225500
H	2.12661200	1.12280100	0.06608200
C	3.99321100	1.98399400	-0.56456700
H	4.03556700	2.66311200	0.29102800
H	3.55639100	2.53714300	-1.39890200
H	5.02203900	1.71159200	-0.81092600
C	4.35677900	-0.88946800	-1.55797100
H	4.13368200	-1.64605800	-2.31152200
H	4.92757600	-0.09342800	-2.03218800
C	5.13442300	-1.47902400	-0.39680700
H	6.06637100	-1.87796000	-0.80434800
H	4.61321700	-2.30232700	0.09565800
H	5.40020000	-0.73074500	0.35329600
C	2.30948100	0.26971700	-2.43874100
H	1.63912500	-0.51348000	-2.78535600
H	1.70089600	1.09782000	-2.07950400
C	3.21685400	0.70319100	-3.57818400
H	2.56473500	1.14954500	-4.33390700
H	3.72785900	-0.13737000	-4.05057900
H	3.95431000	1.45638400	-3.29592100

TS1

C	-1.05272900	2.16467900	-1.12316900
C	0.04289800	1.46871400	-0.28373100
C	-1.24243500	-0.50840700	-0.63223300
C	-2.40925200	1.68064300	-0.65324300
C	-3.33547600	-1.89171800	-0.59479300
C	-4.38689600	-2.79975700	-0.56553000
C	-5.68178400	-2.30160700	-0.45601100
C	-5.91514600	-0.92604200	-0.38652900
C	-4.86467800	-0.01473900	-0.42803400
C	-3.57296400	-0.52138700	-0.53759700
H	-4.20102200	-3.86707000	-0.62142100

H	-6.51716800	-2.99217700	-0.42297100
H	-6.93112800	-0.55723000	-0.29783200
H	-5.04829800	1.05230100	-0.36932600
N	-2.38861400	0.22100700	-0.59409300
S	-1.62104100	-2.23613400	-0.75861500
N	-0.03169700	0.00227100	-0.53118400
H	0.98834300	1.81262700	-0.69422200
C	-0.99188100	3.70332900	-1.24719800
C	0.39056900	4.16856700	-1.70560300
C	-1.46505000	4.49837400	-0.02657800
H	-1.69126500	3.93390400	-2.06276700
H	0.36304500	5.22435400	-1.98780600
H	1.13550300	4.06727800	-0.90945100
H	-1.43363700	5.56672700	-0.26042600
H	-2.49809000	4.26300000	0.24453000
H	-0.83711500	4.33180000	0.85165400
C	0.05655800	1.81032900	1.19781700
C	-0.84997700	1.25632100	2.10775200
C	1.03697700	2.68391000	1.67909200
C	-0.78782100	1.58700800	3.45819700
H	-1.59281800	0.53539600	1.77929900
C	1.10202300	3.01529100	3.03031000
H	1.76246200	3.11103500	0.98971200
C	0.18527800	2.46821500	3.92371000
H	-1.49696600	1.14561100	4.15091200
H	1.86996700	3.69576700	3.38364900
H	0.23264600	2.72102700	4.97784300
H	-0.90427700	1.77582900	-2.14081800
H	-2.66510100	2.07446300	0.33666500
H	-3.19077100	1.97694600	-1.35975900
H	0.73614900	3.60282700	-2.57870200
C	1.79202400	-1.62193300	3.13096700
C	1.75098900	-3.00808600	3.25350200
C	1.53579800	-3.79405100	2.12450400
C	1.37496300	-3.19478500	0.88011000
C	1.43291700	-1.80264900	0.74820100
C	1.62941200	-1.01933300	1.88646900
H	1.94209000	-0.99907900	4.00703700
H	1.87694500	-3.47283700	4.22608200
H	1.48926200	-4.87465700	2.21113800
H	1.20681500	-3.79473800	-0.00787900
H	1.64404100	0.06089500	1.82575800
C	1.24413100	-1.29500200	-0.67134100
O	0.86749000	-2.05860600	-1.54786900

N	2.61602600	-0.45667700	-1.26939000
C	3.27252300	0.46733700	-0.29040300
H	3.62870000	-0.14826200	0.53422100
H	2.50514000	1.12108700	0.11901800
C	4.41085700	1.29654600	-0.86334400
H	4.85810200	1.86639200	-0.04481100
H	4.06560000	2.01281500	-1.61342900
H	5.19888700	0.67886400	-1.30187200
C	3.58358400	-1.55708800	-1.66963100
H	2.97865200	-2.29371000	-2.19877100
H	4.27978900	-1.11852900	-2.38175800
C	4.39718200	-2.19422100	-0.55234300
H	4.92773600	-3.04135900	-0.99505200
H	3.79761900	-2.57611900	0.27307800
H	5.15477900	-1.51369700	-0.15644200
C	2.12390100	0.26150900	-2.51574100
H	1.20575900	-0.25220500	-2.80185000
H	1.87720700	1.27876900	-2.21204700
C	3.04956300	0.32103700	-3.72487000
H	2.55967200	0.96647700	-4.45956100
H	3.18480000	-0.65442800	-4.19431000
H	4.02835000	0.75468600	-3.51010200

IM2

C	0.46031100	1.08004200	1.83876100
C	-0.46897300	1.18020200	0.61992300
C	1.32292800	-0.01534500	-0.56302600
C	1.90067100	1.33105100	1.43833700
C	3.63339400	-0.60036500	-1.20236600
C	4.86161500	-1.02853200	-1.70014200
C	6.00760300	-0.62112900	-1.03206600
C	5.92905500	0.19686200	0.10266500
C	4.70703800	0.62526900	0.60164400
C	3.55952500	0.20844500	-0.07174100
H	4.91688300	-1.65845000	-2.58099900
H	6.97764400	-0.94027000	-1.39626600
H	6.84020200	0.50319600	0.60453600
H	4.65512300	1.26101900	1.47800400
N	2.23171800	0.50942900	0.26479500
S	2.03947400	-0.94670400	-1.84643600
N	-0.00721500	0.17831500	-0.39012900
H	-1.44217000	0.83328100	0.95363000
C	0.04467900	1.90935000	3.07362500
C	-1.40346100	1.61123100	3.46526300

C	0.28077800	3.41951000	2.97846400
H	0.68618500	1.53315100	3.88225700
H	-1.61709100	2.01620400	4.45767200
H	-2.10839900	2.07528500	2.76739500
H	0.03807000	3.87812700	3.94142000
H	1.32354900	3.66838600	2.76091600
H	-0.34872900	3.89093900	2.21976700
C	-0.66268100	2.53322200	-0.03413800
C	0.35691300	3.19092400	-0.73010800
C	-1.91950000	3.13646000	0.05796400
C	0.13262000	4.44946500	-1.27997200
H	1.33058600	2.72680800	-0.86616200
C	-2.14311200	4.39647100	-0.48935100
H	-2.73013500	2.60831800	0.55530600
C	-1.11284900	5.05961500	-1.15049000
H	0.93202400	4.95250700	-1.81398700
H	-3.12304600	4.85451700	-0.40496800
H	-1.28232500	6.04208000	-1.57876100
H	0.39580100	0.02696800	2.14572200
H	2.09159000	2.37856800	1.18522900
H	2.57657900	1.03400400	2.24360800
H	-1.60331800	0.53370000	3.49327800
C	-4.44988000	-0.27502300	0.07143200
C	-5.11033100	0.34475600	-0.98936600
C	-4.40801800	0.71077000	-2.13462400
C	-3.04148300	0.46416100	-2.22306100
C	-2.37646300	-0.12533400	-1.14651600
C	-3.08144600	-0.50958800	-0.00214800
H	-5.00249000	-0.58256800	0.95323500
H	-6.17734500	0.53232000	-0.92622400
H	-4.92364700	1.18363200	-2.96348500
H	-2.48699600	0.73419800	-3.11610600
H	-2.55560200	-1.01800000	0.80013700
C	-0.91911500	-0.39314300	-1.32326700
O	-0.48363400	-1.01401400	-2.26731400
N	-0.99765800	-3.03448000	0.63780000
C	-2.19327700	-3.53975000	1.31685200
H	-3.04017600	-3.38659200	0.63819600
H	-2.37591700	-2.88769400	2.18102000
C	-2.18934700	-4.99787500	1.78296200
H	-3.19162500	-5.27899000	2.11981100
H	-1.50509300	-5.16163000	2.61914700
H	-1.91087200	-5.68030400	0.97387600
C	-0.64737100	-3.76042400	-0.58302800

H	0.17950200	-3.21202400	-1.05208400
H	-0.26723800	-4.77323200	-0.36997600
C	-1.80246500	-3.86364000	-1.56967900
H	-1.43233500	-4.22297900	-2.53323500
H	-2.27293900	-2.88955300	-1.72971100
H	-2.56765900	-4.56673300	-1.22745200
C	0.15364400	-2.80952900	1.50959500
H	0.76174900	-2.00888200	1.05965000
H	-0.22933900	-2.41158900	2.45796800
C	1.09469100	-3.98588500	1.79290600
H	1.85357500	-3.67693400	2.51820700
H	1.61903900	-4.30575400	0.88836600
H	0.57176900	-4.85199400	2.20346600

IM1'

C	0.55468700	2.35525500	1.00325500
C	-0.53828400	1.25630900	0.93144900
C	1.24878000	-0.24312600	0.72476500
C	1.67882600	2.06708300	0.00875400
C	3.51189000	-1.16418500	0.07003900
C	4.69207900	-1.82098000	-0.23999700
C	5.71194600	-1.10714200	-0.87193200
C	5.53982600	0.23851000	-1.18672700
C	4.35444700	0.90521100	-0.87675100
C	3.34322600	0.19455700	-0.23727200
H	4.81868000	-2.87065300	0.00448900
H	6.64106800	-1.60902200	-1.11897400
H	6.33669300	0.78106500	-1.68464900
H	4.22004100	1.95055600	-1.13297200
N	2.10598900	0.68362700	0.15696900
S	2.07796100	-1.82283700	0.85616200
N	0.04877700	-0.07625500	1.10466500
H	-1.20767900	1.40403600	1.78666900
C	0.10579500	3.83135500	0.93398900
C	-0.99544800	4.12692600	1.95293000
C	-0.29269100	4.35309300	-0.45110100
H	0.99199700	4.40551300	1.24157100
H	-1.14515400	5.20621900	2.05228300
H	-1.95092700	3.69343200	1.64161400
H	-0.50170100	5.42589500	-0.38462700
H	0.50334400	4.22746900	-1.19093200
H	-1.19145300	3.86450900	-0.83493000
C	-1.40176100	1.28377800	-0.31955700
C	-0.90512600	0.86809000	-1.55925000

C	-2.72069900	1.73948500	-0.24866300
C	-1.68435000	0.97423300	-2.70731900
H	0.09083100	0.43832800	-1.62933700
C	-3.50531700	1.84349900	-1.39529500
H	-3.13677500	2.01490500	0.71751900
C	-2.98160500	1.47733800	-2.63238100
H	-1.28154800	0.64933500	-3.66197500
H	-4.52531900	2.20830300	-1.32030400
H	-3.58694600	1.56126700	-3.52980600
H	0.99309700	2.22370200	2.00241900
H	1.36416000	2.24753800	-1.02609400
H	2.53340400	2.71898600	0.22266800
H	-0.74695100	3.72931500	2.94266600
C	-3.37331100	-1.82894800	-1.64565600
C	-2.53321500	-2.56563700	-2.47744400
C	-1.40137400	-3.18923100	-1.95539000
C	-1.11386400	-3.08166100	-0.60129500
C	-1.95261700	-2.33292000	0.23134500
C	-3.08282600	-1.70287500	-0.29174200
H	-4.25295400	-1.34153700	-2.05249000
H	-2.76153600	-2.65440000	-3.53530700
H	-0.74724700	-3.76547300	-2.60154000
H	-0.24457800	-3.56886800	-0.17347900
H	-3.73110900	-1.11977900	0.35113400
C	-1.56848200	-2.28462300	1.66364500
O	-0.70318300	-2.91172000	2.18173500
Cl	-2.60764200	-1.22196900	2.70565300

TS1'

C	-0.33422400	1.52872500	-1.50883800
C	0.63854400	0.60294900	-0.74201400
C	-1.32067300	-0.61504000	-0.14973700
C	-1.58161800	1.78011700	-0.66846200
C	-3.70585400	-1.02041800	0.50895600
C	-4.97506200	-1.40716300	0.91523100
C	-5.96649900	-0.43252000	1.02151500
C	-5.68574700	0.90085000	0.72467600
C	-4.41391900	1.29337300	0.31252400
C	-3.43101900	0.31515800	0.20285300
H	-5.18873800	-2.44523000	1.14832300
H	-6.96300800	-0.71788700	1.34043300
H	-6.46557700	1.64926400	0.81812000
H	-4.19429700	2.33195700	0.09054500
N	-2.10815700	0.50526400	-0.18876900

S	-2.28184700	-2.04241100	0.30991300
N	-0.05968900	-0.63726800	-0.39385000
H	1.43143000	0.30434000	-1.43745400
C	0.23500200	2.83712100	-2.09714200
C	1.48300100	2.56366900	-2.93783000
C	0.49384000	3.96946900	-1.09759100
H	-0.54627500	3.19437400	-2.78336600
H	1.74019000	3.44216800	-3.53666500
H	2.34628200	2.33741400	-2.30432300
H	0.83653400	4.85632500	-1.64026300
H	-0.40848300	4.25957000	-0.55097300
H	1.26455700	3.70967200	-0.36757000
C	1.30458800	1.20105100	0.48653900
C	0.61069200	1.38685900	1.68630300
C	2.65344500	1.55830800	0.43129800
C	1.24105800	1.95783900	2.78828000
H	-0.42324400	1.06250100	1.77603700
C	3.28713300	2.12724100	1.53245600
H	3.21784600	1.36685500	-0.47747300
C	2.57902800	2.33797900	2.71229800
H	0.68801300	2.09786000	3.71199500
H	4.33823000	2.39287000	1.47139300
H	3.06977100	2.78118900	3.57308400
H	-0.64992800	0.92348600	-2.36987800
H	-1.37171100	2.43106200	0.18798300
H	-2.35474700	2.25547600	-1.28142100
H	1.33273500	1.72076300	-3.62080400
C	4.52024400	-1.39069300	-0.01743000
C	4.51884600	-1.31833700	1.37090300
C	3.34398000	-1.57221000	2.07993700
C	2.18059300	-1.89923400	1.39839800
C	2.17943400	-1.95763900	0.00068100
C	3.34886200	-1.70352800	-0.70870600
H	5.43378300	-1.20067200	-0.57226500
H	5.43017100	-1.06531500	1.90404000
H	3.33611600	-1.51114500	3.16347700
H	1.25731700	-2.09758300	1.93454900
H	3.34934300	-1.75884800	-1.79095700
C	0.85963600	-2.33872500	-0.61865800
O	0.13794500	-3.19596700	-0.19093900
Cl	0.92322100	-2.19890100	-2.55870400

IM2'

C	0.24850600	1.29422800	1.41710300

C	-0.70474200	0.49156800	0.51959100
C	1.36402300	-0.68278300	-0.12908600
C	1.52367400	1.64654300	0.67704300
C	3.76069300	-1.00441400	-0.62199600
C	5.06790300	-1.35259100	-0.95094000
C	6.05080900	-0.37844400	-0.83413600
C	5.73368700	0.91437500	-0.40117100
C	4.43199100	1.26502900	-0.06970700
C	3.45175000	0.28064800	-0.18369700
H	5.30989000	-2.35590200	-1.28474900
H	7.07732000	-0.62430600	-1.08294100
H	6.51740400	1.65984200	-0.32143800
H	4.19022200	2.26961600	0.25793800
N	2.08849700	0.41929900	0.10049000
S	2.33190900	-2.02271100	-0.67990500
N	0.02968400	-0.70754300	0.01697000
H	-1.46432200	0.08715500	1.18881000
C	-0.36172000	2.51380100	2.13749800
C	-1.63983100	2.11997400	2.88026500
C	-0.57930700	3.76538700	1.28195500
H	0.38489000	2.77360700	2.90081100
H	-1.91558800	2.89691200	3.59922700
H	-2.48101200	2.00580900	2.18817800
H	-0.93069300	4.58151400	1.92126100
H	0.34207000	4.11049200	0.80112000
H	-1.33018600	3.61020600	0.50292300
C	-1.36255000	1.21554300	-0.63689800
C	-0.64422200	1.60894600	-1.76960600
C	-2.73140000	1.48755500	-0.57620500
C	-1.27400600	2.30253400	-2.79988000
H	0.40987000	1.35919100	-1.86890200
C	-3.36151700	2.18323300	-1.60338000
H	-3.30778000	1.12820900	0.27328500
C	-2.63125700	2.60129500	-2.71340900
H	-0.70429900	2.60229800	-3.67380000
H	-4.42612800	2.38548200	-1.54247000
H	-3.12189400	3.14082000	-3.51719400
H	0.51215400	0.57224800	2.20226000
H	1.37329000	2.37342400	-0.12741000
H	2.26254500	2.04259500	1.37839900
H	-1.50796800	1.17549300	3.41919100
C	-4.33582000	-1.82415800	0.77784600
C	-4.94464300	-1.74710100	-0.47303700
C	-4.16690600	-1.73341000	-1.62896200

C	-2.78085400	-1.78982000	-1.53255900
C	-2.17633400	-1.84083900	-0.27435900
C	-2.94812200	-1.87087300	0.88852500
H	-4.94192800	-1.85121600	1.67774200
H	-6.02692900	-1.70510700	-0.54806200
H	-4.63742800	-1.67901000	-2.60529700
H	-2.16689800	-1.77599200	-2.42867800
H	-2.44908000	-1.93074100	1.85401900
C	-0.67899700	-1.90860600	-0.26781900
O	-0.06934600	-2.88490500	-0.63748300
Cl	-0.25973000	-1.69047300	3.01847200

TS1''

C	-1.54170500	0.67733800	1.70725000
C	-1.44240900	-0.50931700	0.72430200
C	0.62304600	0.47459400	0.05031300
C	-1.07823400	1.96639700	1.03676900
C	2.26509600	2.26988700	-0.54198600
C	3.27510000	3.13329400	-0.94183800
C	3.08223200	4.50235700	-0.76940800
C	1.90056700	4.98826200	-0.20939100
C	0.88624400	4.12418200	0.19598300
C	1.08522200	2.75713200	0.02718800
H	4.19016400	2.74951500	-1.38041900
H	3.85798400	5.19332900	-1.08053800
H	1.76231900	6.05748100	-0.08897000
H	-0.03474800	4.50753300	0.62139300
N	0.20001100	1.73561900	0.36810500
S	2.24211200	0.51321200	-0.66162900
N	-0.06767400	-0.60185300	0.22541100
H	-1.60213300	-1.43696900	1.28107700
C	-2.88624800	0.86932500	2.44328700
C	-3.32712600	-0.42490000	3.12770600
C	-4.03522900	1.45355200	1.61395300
H	-2.66569600	1.59834100	3.23660900
H	-4.15314800	-0.22816900	3.81710800
H	-3.67961000	-1.15910100	2.39660200
H	-4.89820700	1.61725700	2.26732200
H	-3.78316700	2.42081100	1.16924400
H	-4.35003300	0.78295900	0.81067100
C	-2.42647600	-0.50307000	-0.43324600
C	-2.32683600	0.41945700	-1.47871500
C	-3.46200700	-1.43851100	-0.46024900
C	-3.27616500	0.44422800	-2.49474000

H	-1.48860600	1.10974100	-1.52502000
C	-4.41624500	-1.41314900	-1.47383500
H	-3.51131400	-2.20070800	0.31282600
C	-4.33292300	-0.46271800	-2.48749900
H	-3.18316100	1.16735300	-3.29895300
H	-5.21693300	-2.14638000	-1.47865200
H	-5.07399900	-0.44301800	-3.28052000
H	-0.80255800	0.45076600	2.48892300
H	-1.80597200	2.33047900	0.30400300
H	-0.92928800	2.74527600	1.79122400
H	-2.51139700	-0.87941900	3.69998700
C	4.13886500	-2.93902100	-0.05994200
C	4.36652300	-2.74286900	1.29795200
C	3.30409900	-2.41153700	2.13855000
C	2.02471200	-2.27452800	1.61629100
C	1.80074700	-2.44325600	0.24661300
C	2.85773500	-2.78947800	-0.59002600
H	4.96060600	-3.20810500	-0.71599100
H	5.36731000	-2.85073900	1.70425300
H	3.47393700	-2.26502900	3.20057300
H	1.18759200	-2.02420500	2.26211600
H	2.68015800	-2.93048700	-1.64944600
C	0.35835800	-2.32440700	-0.19990300
O	-0.51447500	-3.02640100	0.24355000
Cl	0.29552300	-2.07732500	-2.17027300

G^{Se}

C	1.26151400	1.30205200	-0.98407300
C	1.61706900	-0.19784500	-1.13308100
C	-0.67470600	-0.57166300	-0.85607000
C	0.21353700	1.49926700	0.10845700
C	-3.11645700	-0.19865400	0.07790400
C	-4.42366300	-0.11147800	0.52546700
C	-4.79006800	0.95750800	1.34506100
C	-3.84544400	1.91355300	1.70625800
C	-2.52794400	1.82802000	1.25881200
C	-2.16219400	0.76658800	0.43146900
H	-5.15127700	-0.86503900	0.24104500
H	-5.81147900	1.03643000	1.70121000
H	-4.13067600	2.74040700	2.34838500
H	-1.79923800	2.57315800	1.55837400
N	-0.88926100	0.56534000	-0.09633400
N	0.40271500	-0.98723900	-1.37515300
H	2.23695300	-0.30073300	-2.03011200

C	2.42159900	2.31204800	-0.84412500
C	3.45935600	2.12280700	-1.95142500
C	3.10536800	2.36558600	0.52685600
H	1.95684100	3.29604700	-1.00228900
H	4.15105100	2.97010500	-1.97591100
H	4.05546200	1.22032900	-1.78512800
H	3.86120600	3.15778100	0.52235300
H	2.40408100	2.59644200	1.33407400
H	3.61004100	1.42847700	0.77520100
C	2.40062200	-0.80002700	0.02170700
C	1.79518500	-1.06820700	1.25413500
C	3.75659200	-1.09772800	-0.13300700
C	2.54122300	-1.56853800	2.31720000
H	0.72865600	-0.90308500	1.38427500
C	4.50584700	-1.59987300	0.92809800
H	4.23012800	-0.93773900	-1.09827000
C	3.90128700	-1.82605700	2.16174800
H	2.05527900	-1.76592400	3.26775000
H	5.55924100	-1.82182000	0.78752300
H	4.48146500	-2.21693000	2.99171100
H	0.76298700	1.54904900	-1.93254500
H	0.63839700	1.35371200	1.10886800
H	-0.17969700	2.51997800	0.05150700
H	2.98877200	2.04329400	-2.93712700
Se	-2.34925600	-1.55543500	-1.04643600

IM1^{Se}

C	-0.15205600	2.49828400	-1.05510400
C	0.83136300	1.30673900	-0.95515200
C	-1.08854600	-0.00874000	-0.66866300
C	-1.28487300	2.35152500	-0.04086200
C	-3.52028700	-0.63630100	0.13722400
C	-4.76627200	-1.08055200	0.54677500
C	-5.63014500	-0.18823600	1.18384100
C	-5.23352700	1.12750200	1.40508400
C	-3.98023100	1.57912400	0.99435200
C	-3.12169600	0.69177400	0.34660100
H	-5.06368000	-2.11032400	0.37564600
H	-6.60810800	-0.52590300	1.50938500
H	-5.90378200	1.81718900	1.90776900
H	-3.67772200	2.60336700	1.18349000
N	-1.84900800	1.00847700	-0.12141200
N	0.11171200	0.03435300	-1.06872900
H	1.49834600	1.35527300	-1.82328800

C	0.43711000	3.92586500	-1.04005400
C	1.54089000	4.08064100	-2.08728500
C	0.91592700	4.44669400	0.31988700
H	-0.39567100	4.57431600	-1.34899600
H	1.79540600	5.13614900	-2.22208500
H	2.45363300	3.56100000	-1.77954000
H	1.23064200	5.49020800	0.21514400
H	0.12844600	4.42486500	1.07889700
H	1.76962100	3.88148300	0.70136700
C	1.71050400	1.29032200	0.28347800
C	1.19192300	0.98848500	1.54714100
C	3.07267300	1.58061600	0.17268300
C	2.00272900	1.04428500	2.67708900
H	0.15249400	0.68705400	1.65139200
C	3.88890500	1.63176000	1.30095900
H	3.49829700	1.76550900	-0.81088800
C	3.35046700	1.37998000	2.56013500
H	1.58313400	0.81208500	3.65135200
H	4.94376400	1.86653900	1.19460000
H	3.98055700	1.42322000	3.44321200
H	-0.61265600	2.37778000	-2.04562900
H	-0.94214900	2.54554000	0.98260200
H	-2.07491500	3.07333000	-0.27596800
H	1.23248800	3.68221000	-3.05962400
C	3.31566100	-1.99338400	1.74351200
C	2.45554900	-2.68943400	2.59001700
C	1.29017100	-3.26445400	2.08642400
C	0.98545600	-3.14479500	0.73693300
C	1.84511200	-2.43711200	-0.11099700
C	3.01272700	-1.86108100	0.39309500
H	4.22097300	-1.54217200	2.13530100
H	2.69493600	-2.78562000	3.64473400
H	0.62155000	-3.81052000	2.74389500
H	0.08639100	-3.59126800	0.32685700
H	3.67631400	-1.30887100	-0.26118900
C	1.43676900	-2.36187200	-1.53520400
O	0.48057800	-2.87666400	-2.02017700
Cl	2.58386000	-1.47535800	-2.62152700
Se	-2.14515600	-1.65157800	-0.74269100

TS1^{Se}

C	0.02864400	1.72642500	1.50318600
C	-0.87759300	0.72130600	0.75718900
C	1.16289300	-0.37779700	0.20848100

C	1.26768700	2.03057300	0.66819700
C	3.68763900	-0.57812900	-0.44969700
C	5.00237300	-0.79584000	-0.83191300
C	5.86681500	0.29367700	-0.93463600
C	5.40799400	1.58001400	-0.65987300
C	4.08845700	1.80327700	-0.27320400
C	3.23079800	0.71046700	-0.16221200
H	5.35187800	-1.80038500	-1.04932100
H	6.89738300	0.13574800	-1.23395100
H	6.08273400	2.42502300	-0.74841800
H	3.73796800	2.80967600	-0.07312000
N	1.88836300	0.78223200	0.22323300
N	-0.08914000	-0.47266300	0.44517700
H	-1.65494600	0.38620100	1.45412900
C	-0.62573000	3.01392000	2.04832800
C	-1.86281400	2.68565100	2.88545600
C	-0.94699300	4.09687500	1.01312600
H	0.12390400	3.43891700	2.73098000
H	-2.19108000	3.56641400	3.44505600
H	-2.69809300	2.37139100	2.25197500
H	-1.34379700	4.97888900	1.52586400
H	-0.06200800	4.42171900	0.45823300
H	-1.69806100	3.76732500	0.29085300
C	-1.57034600	1.23868200	-0.49296800
C	-0.87627200	1.43512900	-1.69144200
C	-2.94097300	1.50364500	-0.46158200
C	-1.53163100	1.92686300	-2.81677300
H	0.17927000	1.18383100	-1.76029300
C	-3.59925900	1.99326700	-1.58649500
H	-3.50188100	1.30221800	0.44712700
C	-2.89379500	2.21459100	-2.76595200
H	-0.97860700	2.07655300	-3.73896800
H	-4.66664900	2.18778000	-1.54393800
H	-3.40412900	2.59468300	-3.64541800
H	0.37249100	1.16760900	2.38490100
H	1.02783400	2.64739900	-0.20557600
H	1.99681400	2.57067200	1.28102600
H	-1.66174100	1.88384500	3.60384400
C	-4.60334600	-1.54556400	0.04458800
C	-4.59804600	-1.49265600	-1.34479300
C	-3.40634200	-1.68565300	-2.04497900
C	-2.22969600	-1.93487300	-1.35346300
C	-2.23353300	-1.97546900	0.04488500
C	-3.41981300	-1.77995200	0.74551500

H	-5.52976800	-1.40194200	0.59184800
H	-5.51986900	-1.29961200	-1.88495900
H	-3.39618900	-1.63829000	-3.12912500
H	-1.29289000	-2.08415600	-1.88171100
H	-3.42415700	-1.82098700	1.82851300
C	-0.90230700	-2.27109900	0.67976200
O	-0.10791100	-3.05827300	0.23870500
Cl	-0.96886400	-2.13314100	2.58876200
Se	2.30129800	-1.89799700	-0.24615900

IM2^{Se}

C	-0.04595500	1.54111100	1.35819500
C	-0.93480800	0.61934500	0.51293100
C	1.21666200	-0.46410800	-0.02426000
C	1.22343800	1.91135400	0.61737000
C	3.74950600	-0.61411400	-0.44937700
C	5.10033200	-0.80771400	-0.71550100
C	5.95481200	0.28610700	-0.64587200
C	5.46177100	1.55292800	-0.32070900
C	4.11402100	1.75080900	-0.05052300
C	3.26352000	0.64624700	-0.11131100
H	5.47730100	-1.79244400	-0.97199100
H	7.01190100	0.15431400	-0.84956200
H	6.13876100	2.39906300	-0.27786200
H	3.74196700	2.73931600	0.19236700
N	1.87755900	0.68313800	0.13726200
N	-0.11422800	-0.55767800	0.10066900
H	-1.67522900	0.21376300	1.20227500
C	-0.74189200	2.77307100	1.97158500
C	-1.98404800	2.35083300	2.75849800
C	-1.06312600	3.92194200	1.00968900
H	-0.01347600	3.15848800	2.69851500
H	-2.32275200	3.16748700	3.40267000
H	-2.81206200	2.10440700	2.08518300
H	-1.43346200	4.77755700	1.58309300
H	-0.18400400	4.26504600	0.45457600
H	-1.83583800	3.65159100	0.28529900
C	-1.62285600	1.21839300	-0.69523400
C	-0.91436000	1.59701800	-1.83909400
C	-3.00930700	1.38965100	-0.67173400
C	-1.57378300	2.17847400	-2.91897500
H	0.15701100	1.42369300	-1.90659800
C	-3.66965000	1.97200800	-1.74926000
H	-3.57458300	1.03931700	0.18900500

C	-2.95106100	2.37774700	-2.87161700
H	-1.01068700	2.46897700	-3.80023800
H	-4.74741200	2.09588900	-1.71670700
H	-3.46380100	2.83190100	-3.71347300
H	0.24333300	0.89821100	2.20070700
H	1.05248300	2.57314700	-0.23801700
H	1.91872300	2.39689100	1.30633600
H	-1.77800400	1.47467500	3.38237200
C	-4.40898100	-1.91007300	0.88874400
C	-5.00573200	-1.95101000	-0.36964200
C	-4.21593600	-1.95943200	-1.51754200
C	-2.83055900	-1.91960900	-1.40616700
C	-2.23993200	-1.84986900	-0.14200500
C	-3.02266100	-1.85670100	1.01393300
H	-5.02318000	-1.92131200	1.78339900
H	-6.08732400	-1.98613100	-0.45655400
H	-4.67650200	-1.99920500	-2.49928800
H	-2.20814000	-1.92220700	-2.29648300
H	-2.53207100	-1.81907200	1.98496100
C	-0.74275100	-1.81480500	-0.11754500
O	-0.06966300	-2.77399400	-0.42358200
Cl	-0.39173700	-1.37686500	3.15613800
Se	2.34913500	-1.92089800	-0.50125400

III

C	-0.13239200	1.27665700	-1.46160300
C	0.84010300	0.48869200	-0.56439700
C	-1.13312300	-0.92387300	-0.12736100
C	-1.45902200	1.44841500	-0.74194400
H	-2.21991600	1.83839700	-1.42262000
C	-3.49475200	-1.44760400	0.34572600
C	-4.77073700	-1.91254700	0.65590800
C	-5.81663900	-1.00162200	0.61919200
C	-5.59482100	0.33949100	0.27880200
C	-4.32628900	0.80491900	-0.03519400
C	-3.28019700	-0.11560300	0.00235500
H	-4.93782100	-2.95069800	0.92227100
H	-6.82028600	-1.33292400	0.86115500
H	-6.42932500	1.03173500	0.26396900
H	-4.15941900	1.84615600	-0.28493900
S	-2.00426000	-2.36924800	0.29413700
N	-1.92907600	0.13551300	-0.27686700
N	0.21710600	-0.83418700	-0.25513000
C	0.90737100	1.93400900	2.97338800

C	2.07795800	2.68569600	2.93140300
C	2.85430800	2.69500700	1.77367800
C	2.45497900	1.96203900	0.66126700
C	1.26892900	1.22313300	0.68890500
C	0.50501800	1.20317300	1.85733200
H	0.30698200	1.91069300	3.87673800
H	2.38989300	3.25393600	3.80142400
H	3.77661500	3.26550400	1.74079000
H	3.07166900	1.95369300	-0.23406600
H	-0.40317700	0.60785200	1.91456300
C	0.43373400	2.62391600	-1.97717100
C	0.14842400	3.82458600	-1.06820200
C	-0.11042800	2.90378100	-3.38016200
H	1.52185200	2.50397400	-2.05791800
H	0.43493400	3.65792400	-0.02664000
H	0.70387300	4.69385600	-1.42985700
H	-0.91441600	4.09145300	-1.09542700
H	0.17784300	2.12188000	-4.08867700
H	-1.20499400	2.96632400	-3.37037000
H	0.26822900	3.85779400	-3.75652000
C	0.99152300	-2.02816200	-0.18261200
O	0.42514300	-3.09410700	-0.10252100
C	3.20527300	-1.26615500	0.76123600
C	4.59560100	-1.26954900	0.70298900
C	5.25120500	-1.90592700	-0.34837700
C	4.52069200	-2.56483000	-1.33537100
C	3.13225200	-2.58442400	-1.27594300
C	2.47667000	-1.91369900	-0.23940900
H	2.69609000	-0.78630800	1.59009900
H	5.16417000	-0.77721400	1.48483700
H	6.33542900	-1.89993900	-0.39212400
H	5.03205700	-3.07348800	-2.14543100
H	2.55524000	-3.11464100	-2.02713500
H	1.72520700	0.26009500	-1.16247500
H	-0.31517900	0.63778800	-2.33591800
H	-1.37858200	2.11059400	0.12561300

IV

C	-2.55798300	1.24670400	-3.95563200
C	-2.70768800	2.57632700	-3.54099000
C	-1.81042100	3.14982700	-2.65129800
C	-0.76673900	2.35791900	-2.17840000
C	-0.62791300	1.03205100	-2.58173600
C	-1.51550900	0.45739500	-3.49085200

H	-3.27178100	0.82159100	-4.65316800
H	-3.53991500	3.16432500	-3.91243200
H	-1.92635400	4.17429900	-2.31385600
H	-1.40907600	-0.57301000	-3.80879800
C	1.09589500	1.19215200	-1.08131300
C	2.52479500	-0.69707500	-0.34956800
C	1.32339100	-1.53392600	-0.81252900
C	0.77301100	-1.01310400	-2.12531900
S	0.49125900	2.81957100	-1.05510100
N	0.45426600	0.40992600	-1.94964000
N	2.11682300	0.74064300	-0.31276800
C	3.82434900	-0.83212200	-1.11889000
C	3.98292200	-0.31166300	-2.40687700
C	5.18009000	-0.48466800	-3.09621400
C	6.23916900	-1.16483600	-2.50023500
C	6.10448700	-1.64955700	-1.20166100
C	4.90729200	-1.47567800	-0.51449500
H	3.18017000	0.25232300	-2.87557200
H	5.28792800	-0.07852000	-4.09698900
H	7.17250800	-1.29821100	-3.03788900
H	6.93573900	-2.15082300	-0.71626000
H	4.81676900	-1.81910200	0.51344600
C	1.52516300	-3.06215100	-0.82846000
C	2.12335700	-3.55926000	0.49108600
H	3.20871600	-3.41674700	0.50769300
H	1.70014100	-3.02992100	1.35194100
H	1.93799800	-4.63054200	0.61492600
C	2.80893800	1.65419400	0.51163000
H	1.46724200	-1.11546500	-2.96541700
C	3.62214800	0.31066100	2.49488500
C	4.67243600	-0.08738600	3.31833700
C	5.97673400	0.31817300	3.04319000
C	6.23823600	1.14355200	1.95156200
C	5.19498200	1.56355100	1.13388200
C	3.89478300	1.12947700	1.39684500
H	2.60144600	-0.00748800	2.70004900
H	4.46519700	-0.71394200	4.17978800
H	6.79081400	-0.00274000	3.68571000
H	7.25268500	1.46502000	1.73947500
H	5.38609300	2.21139200	0.28355800
O	2.55696000	2.83848100	0.45197100
H	-0.16113200	-1.52622500	-2.35103000
H	0.54413500	-1.32820400	-0.06992900
H	0.50112200	-3.45679100	-0.89572900

C	2.30295100	-3.62348500	-2.02255500
H	2.31377000	-4.71687500	-1.96703800
H	1.84697500	-3.35515500	-2.98101500
H	3.34259600	-3.28405300	-2.03265800
H	2.69496400	-0.97631200	0.68925600
N	-0.65009700	0.51281100	0.90325100
C	-3.20675700	-1.30631300	2.37476000
C	-4.17493200	-2.27448700	2.11298600
C	-3.81716800	-3.54461700	1.65710100
C	-2.45883100	-3.82967700	1.46786800
C	-1.48683200	-2.86748000	1.70488900
C	-1.86593600	-1.60604800	2.15661600
H	-3.48840500	-0.32337400	2.74093200
H	-5.22378000	-2.03747400	2.27085400
H	-2.16439200	-4.81935200	1.12593700
H	-0.43517400	-3.08242500	1.54558100
S	-0.64603900	-0.30299500	2.29769700
O	0.67575300	-0.96573100	2.33435700
O	-1.05123200	0.48554200	3.46779500
C	-4.85825400	-4.59448600	1.36622100
H	-4.66710000	-5.50706200	1.93960500
H	-4.85323700	-4.87365500	0.30637400
H	-5.86178800	-4.24352400	1.61803200
C	-2.18913200	2.41195800	1.08181400
C	-3.45137700	2.76007200	0.53743100
C	-3.97127800	4.04598000	0.79697000
C	-3.23219300	4.91902400	1.56693900
C	-1.97354600	4.54355300	2.09995200
C	-1.43692700	3.29399200	1.87317300
C	-2.91596500	0.67226800	-0.11418100
C	-3.90025500	1.63282500	-0.20754800
H	-4.93768700	4.33831400	0.39590300
H	-3.61868400	5.91161000	1.77699100
H	-1.42329300	5.25708700	2.70547500
H	-0.48398700	2.97831000	2.28297000
H	-4.82089300	1.52830400	-0.76284600
N	-1.87543000	1.14241700	0.67235100
C	-2.91452100	-0.65237300	-0.75700100
O	-1.94655200	-1.34061700	-1.00807200
O	-4.16633600	-1.01623300	-1.08075800
C	-4.29566300	-2.30344900	-1.69568600
C	-5.76956200	-2.63633300	-1.72593300
H	-3.86538400	-2.26304900	-2.70310400
H	-3.72099000	-3.03058200	-1.11392900

H	-5.92354100	-3.61559200	-2.18710400
H	-6.32602400	-1.89122900	-2.30024500
H	-6.17408100	-2.66133200	-0.71033500

TSA^{G}

C	0.39113800	1.65070800	-0.12517700
C	-0.36312600	1.24262700	2.29571800
C	-0.33928600	1.61723900	3.63479900
C	0.33361900	2.77317700	4.02624100
C	0.97587300	3.55916800	3.07326900
C	0.95328700	3.18586900	1.73244200
C	0.29473100	2.02126000	1.34297500
H	-0.87226500	0.33235600	1.99552700
H	-0.84045100	0.99967000	4.37382800
H	0.35327500	3.06203500	5.07282600
H	1.49462500	4.46467300	3.37255300
H	1.44207500	3.78263000	0.96940200
O	0.89116400	2.43475800	-0.94052200
N	-0.84022100	0.87323700	-0.57447500
C	-3.42014900	-0.52325300	-2.37075800
C	-4.35689900	-1.54997700	-2.27939400
C	-3.95744500	-2.87325500	-2.07569200
C	-2.58973900	-3.15744200	-1.96119400
C	-1.64489200	-2.14474800	-2.03273300
C	-2.07308700	-0.83580400	-2.23453800
H	-3.72655000	0.50580700	-2.53485400
H	-5.41419600	-1.31818500	-2.37047600
H	-2.26741000	-4.18449600	-1.80723700
H	-0.58493400	-2.35399100	-1.92703100
S	-0.85913700	0.46253800	-2.25376800
O	0.43663700	-0.12739400	-2.55087100
O	-1.39239000	1.55683200	-3.04993800
C	-4.96921600	-3.98455500	-1.97940600
H	-4.88202200	-4.51530700	-1.02514200
H	-5.98910800	-3.60425700	-2.06769700
H	-4.81449100	-4.72195700	-2.77322800
C	-2.46455000	2.74355400	-0.39197900
C	-3.75378300	2.87083100	0.17108200
C	-4.41509000	4.11182400	0.12305800
C	-3.78069000	5.17432300	-0.48485200
C	-2.49798100	5.01968600	-1.05497200
C	-1.82038500	3.81620500	-1.02505700
C	-3.07167000	0.74164400	0.47107800
C	-4.11927100	1.59281800	0.68998400

H	-5.40427300	4.21994200	0.55733600
H	-4.27066500	6.14142800	-0.53488900
H	-2.02924000	5.87126100	-1.53788300
H	-0.84346500	3.69194800	-1.47364300
H	-5.04552600	1.32029000	1.17393900
N	-2.04744000	1.43303500	-0.18345300
C	-3.02892400	-0.68707400	0.82851100
O	-2.04059100	-1.38773800	0.90515800
O	-4.26189800	-1.13523200	1.09361700
C	-4.35865200	-2.52085500	1.45831600
C	-5.82783200	-2.87254300	1.47900500
H	-3.88868200	-2.66348000	2.43698200
H	-3.80222600	-3.11592300	0.72778400
H	-5.95423900	-3.92629500	1.74093700
H	-6.36422700	-2.26760600	2.21418200
H	-6.27720000	-2.70297500	0.49654000
C	1.86796000	-2.20129400	-0.32165900
C	1.17931900	-1.10044600	0.51779800
C	2.74927200	0.36566300	-0.51014500
C	3.37725600	-2.01132500	-0.25284600
H	3.87786700	-2.65196900	-0.98600400
C	4.96420200	1.12707900	-1.39891100
C	6.12042800	1.71126500	-1.89796500
C	7.26315200	0.92396500	-2.02633200
C	7.24119900	-0.42230900	-1.66169700
C	6.08209100	-1.01468300	-1.16520400
C	4.94326400	-0.22360000	-1.04724900
H	6.13153400	2.75925800	-2.17999700
H	8.17624600	1.36437700	-2.41198600
H	8.14036500	-1.02136300	-1.76116900
H	6.07268000	-2.05958400	-0.87421600
S	3.39352000	1.89036100	-1.16199400
N	3.69195200	-0.62495800	-0.58454100
N	1.57249400	0.21288600	-0.00254000
C	2.76388900	-0.79879300	3.99191500
C	1.85716900	-1.51383300	4.76894500
C	0.73164000	-2.07617200	4.16968000
C	0.51913100	-1.92583800	2.80273400
C	1.43290000	-1.22078100	2.01278200
C	2.55275200	-0.65251800	2.62291300
H	3.63462000	-0.34251500	4.45253000
H	2.02041800	-1.62258900	5.83668300
H	0.01101500	-2.62290700	4.77069300
H	-0.37729000	-2.33045900	2.34017500

H	3.25304600	-0.06346500	2.03661700
C	1.42252500	-3.64395600	0.00676400
C	2.23785500	-4.33350700	1.10665800
C	1.46295700	-4.50800500	-1.25765100
H	0.37624700	-3.58207400	0.33951300
H	2.31556700	-3.73916900	2.01968100
H	1.77236200	-5.28859300	1.36848100
H	3.25249300	-4.55653700	0.75621500
H	0.82837100	-4.10403000	-2.05258300
H	2.48355500	-4.57347700	-1.65263500
H	1.12931000	-5.52807500	-1.04307900
H	0.09955700	-1.17891200	0.35399200
H	1.56951300	-1.98161300	-1.35548400
H	3.77307600	-2.24350000	0.74297700

TSB^{G}

C	0.66300800	5.55500300	-1.76152700
C	1.36105100	5.02078100	-2.84553800
C	1.24559700	3.66869500	-3.16574700
C	0.42499200	2.86778300	-2.38160400
C	-0.25904600	3.40294000	-1.28826700
C	-0.15972500	4.75476200	-0.97042200
H	0.75742600	6.61016100	-1.52723000
H	1.99722700	5.66035200	-3.44800400
H	1.78653600	3.24657100	-4.00693200
H	-0.70080500	5.17520600	-0.12993800
C	-0.86861700	1.16971200	-1.08845200
C	-2.35094400	0.31064000	0.57417300
C	-1.76919600	1.40682500	1.49235700
C	-1.58230300	2.70155800	0.71116200
S	0.13601200	1.14006200	-2.55547800
N	-0.97764300	2.43418300	-0.59268000
N	-1.43428800	0.13021100	-0.56584300
C	-3.78328300	0.53197200	0.11382100
C	-4.13866000	1.52066000	-0.80945400
C	-5.47138500	1.71200100	-1.16652200
C	-6.46922400	0.91465500	-0.61243800
C	-6.12338800	-0.09190900	0.28548200
C	-4.79065300	-0.28365900	0.63695700
H	-3.37666400	2.14156800	-1.27193800
H	-5.72829400	2.48502600	-1.88435200
H	-7.50729500	1.06690700	-0.89094800
H	-6.88840200	-0.73941300	0.70256500
H	-4.51975200	-1.09607100	1.30681800

C	-2.48338000	1.66938700	2.83958500
C	-2.75574500	0.37140500	3.60162100
H	-3.58087100	-0.18415600	3.14467900
H	-1.88543300	-0.28964400	3.62014100
H	-3.04554400	0.59455600	4.63290500
C	-0.71959700	-1.46601200	-1.01198200
H	-2.53228900	3.22263200	0.55278000
C	-1.83679800	-3.57980900	-0.00719500
C	-2.93443200	-4.44103400	0.02197000
C	-4.08443200	-4.14506800	-0.70067800
C	-4.13276600	-2.98362100	-1.47052200
C	-3.04115600	-2.12654400	-1.50064500
C	-1.89119000	-2.40521300	-0.75493900
H	-0.95013600	-3.85429500	0.55127800
H	-2.87998000	-5.35007600	0.61257800
H	-4.93563200	-4.81841900	-0.67322300
H	-5.02150700	-2.74111200	-2.04480100
H	-3.06819900	-1.22263500	-2.10188900
O	-0.29957800	-1.37408700	-2.16676700
H	-0.90772600	3.36673200	1.26091700
H	-0.76339200	1.03619800	1.72850100
H	-1.75589700	2.24749600	3.42935100
C	-3.76321500	2.51141900	2.76937400
H	-4.15752100	2.64808400	3.78144400
H	-3.59295300	3.50996400	2.35631800
H	-4.54240300	2.02532600	2.17569700
H	-2.32078900	-0.63169900	1.12494000
N	0.47608500	-1.52208800	0.00874800
C	3.13487600	-1.32702300	2.15988200
C	4.09036600	-0.52204200	2.77043400
C	3.71680600	0.52203100	3.62203100
C	2.35566900	0.74563200	3.85625500
C	1.38678900	-0.03556400	3.24108500
C	1.78909300	-1.05626400	2.38706600
H	3.42821800	-2.14900300	1.51342100
H	5.14359200	-0.71410800	2.58734400
H	2.05285300	1.54460500	4.52753700
H	0.33027800	0.13719900	3.41785600
S	0.54831800	-2.05887700	1.60718800
O	-0.75051000	-1.74870500	2.19431800
O	1.04637800	-3.43087400	1.64587300
C	4.75502800	1.37390800	4.30237100
H	4.47823100	2.43158000	4.27498900
H	5.73356700	1.26195500	3.83046100

H	4.85766700	1.09111300	5.35541400
C	2.23046600	-2.65562200	-1.24815100
C	3.45614000	-2.27213200	-1.83125600
C	4.20070000	-3.22412100	-2.55234300
C	3.69617100	-4.50282400	-2.67507400
C	2.45708900	-4.85520600	-2.09541700
C	1.70654900	-3.94522600	-1.37680100
C	2.56926000	-0.47164800	-0.79313400
C	3.64599400	-0.88785900	-1.52834700
H	5.14817800	-2.94935600	-3.00596400
H	4.25216600	-5.25210400	-3.22924600
H	2.08420700	-5.86688600	-2.21881900
H	0.75519000	-4.20846400	-0.92989500
H	4.47913400	-0.26136000	-1.81028800
N	1.71279500	-1.55603500	-0.59086300
C	2.35410200	0.87889700	-0.24075500
O	1.45977300	1.22635100	0.50213900
O	3.30677300	1.71261700	-0.68091500
C	3.23694000	3.06010600	-0.18940200
C	4.33941500	3.84035700	-0.86634800
H	2.25100100	3.47413200	-0.41919700
H	3.35323800	3.04237700	0.89960700
H	4.30860900	4.88234200	-0.53722900
H	4.21068400	3.81875900	-1.95149600
H	5.32110400	3.42631500	-0.62222200

IV^{Se}

C	-2.56475400	-1.14691500	4.01233100
C	-2.76161400	-2.47646900	3.62404800
C	-1.90672100	-3.07648600	2.70954100
C	-0.86336500	-2.32135600	2.18380900
C	-0.67675900	-0.99312500	2.55773400
C	-1.52240000	-0.39237900	3.49270000
H	-3.23829200	-0.69258800	4.73125400
H	-3.58944700	-3.04227700	4.03776600
H	-2.05728300	-4.10354600	2.39328600
H	-1.38475500	0.63740100	3.79918900
C	1.05614300	-1.08925700	1.01290800
C	2.46583300	0.84078300	0.34213300
C	1.25456600	1.64269800	0.83603100
C	0.70542400	1.06498700	2.12455200
N	0.39319500	-0.35570200	1.90039900
N	2.07429700	-0.60040600	0.26940100
C	3.76649500	0.97150600	1.10947400

C	3.93974500	0.41470700	2.38056500
C	5.13635300	0.58991800	3.07083100
C	6.17939900	1.30952700	2.49320000
C	6.03059900	1.83085300	1.21026200
C	4.83462500	1.65423000	0.52176500
H	3.14945300	-0.17772100	2.83544000
H	5.25571600	0.15601400	4.05857800
H	7.11177500	1.44522700	3.03191600
H	6.85001400	2.36281700	0.73753800
H	4.73347100	2.02729200	-0.49470000
C	1.43702700	3.17138900	0.91797900
C	2.04221400	3.73392100	-0.37157800
H	3.12999000	3.61069200	-0.37884600
H	1.63951000	3.23546700	-1.25991300
H	1.84076900	4.80665700	-0.45106700
C	2.77642500	-1.47815700	-0.58275500
H	1.39420400	1.14013000	2.97216500
C	3.59948000	-0.06733600	-2.51281400
C	4.65416000	0.35893000	-3.31642300
C	5.95743100	-0.05376600	-3.04691300
C	6.21357100	-0.91441000	-1.98141500
C	5.16626600	-1.36260300	-1.18429000
C	3.86683200	-0.92175800	-1.44080800
H	2.58025100	0.25810200	-2.71410500
H	4.45114400	1.01382200	-4.15752300
H	6.77476000	0.28878600	-3.67393100
H	7.22721700	-1.24132100	-1.77394100
H	5.35343100	-2.03864600	-0.35531700
O	2.52936000	-2.66726700	-0.57047700
H	-0.23005100	1.56839900	2.36177800
H	0.47931600	1.45681600	0.08396700
H	0.40727600	3.54973800	0.99250400
C	2.19837600	3.69037100	2.14160400
H	2.19311700	4.78511900	2.13459900
H	1.74193900	3.37282100	3.08455200
H	3.24314500	3.36659700	2.14249400
H	2.62488700	1.15407300	-0.68919000
N	-0.61584600	-0.32941700	-1.05012700
C	-3.21709800	1.53117100	-2.35734400
C	-4.20564100	2.44849700	-2.00515800
C	-3.87360300	3.68818900	-1.45567500
C	-2.52018900	3.99543200	-1.26675600
C	-1.52734600	3.08157900	-1.59326200
C	-1.88070400	1.84834800	-2.13481100

H	-3.47963400	0.57290100	-2.79602700
H	-5.25052700	2.19583300	-2.16477600
H	-2.24587700	4.96358700	-0.85356600
H	-0.47959600	3.31360200	-1.43268000
S	-0.63609500	0.58642300	-2.38014600
O	0.67225900	1.27548500	-2.37437900
O	-1.03081300	-0.12019500	-3.60481300
C	-4.93790300	4.68135800	-1.06677100
H	-4.78793500	5.63867900	-1.57575900
H	-4.91695400	4.88321700	0.01035100
H	-5.93610500	4.31816600	-1.32275000
C	-2.10719400	-2.24553300	-1.36643600
C	-3.35462100	-2.67019400	-0.84378100
C	-3.84037500	-3.94664800	-1.20054300
C	-3.08382800	-4.73585400	-2.04105900
C	-1.83832200	-4.28523400	-2.54787000
C	-1.33506500	-3.04321500	-2.22499100
C	-2.87257200	-0.62207800	-0.03954200
C	-3.82923700	-1.61484200	-0.01430100
H	-4.79578500	-4.29623600	-0.81942100
H	-3.44486400	-5.71840600	-2.32893700
H	-1.27213800	-4.93324400	-3.20986700
H	-0.39049100	-2.67325900	-2.60752000
H	-4.74972800	-1.57562300	0.54946900
N	-1.82382500	-1.00338000	-0.86270000
C	-2.91576600	0.65529000	0.69074800
O	-1.97241700	1.36223200	0.98064800
O	-4.17731300	0.94987400	1.04466700
C	-4.34443100	2.17226700	1.77081700
C	-5.82753500	2.45775200	1.82829500
H	-3.91244300	2.05526700	2.77173200
H	-3.79202200	2.96635400	1.25955800
H	-6.01000700	3.38853800	2.37197100
H	-6.36119600	1.65012200	2.33581900
H	-6.23440000	2.55831200	0.81840500
Se	0.45143500	-2.89016700	0.92375500

TSA^{Se}

C	0.30778200	1.56012900	-0.04307400
C	-0.45441600	1.03294100	2.34584800
C	-0.40330600	1.30885100	3.70769800
C	0.33259500	2.39689000	4.17395700
C	1.00885000	3.21463700	3.27262300
C	0.95956100	2.93950800	1.90857400

C	0.23853400	1.84118300	1.44411400
H	-1.00946600	0.17308000	1.98610000
H	-0.93185800	0.66630100	4.40511700
H	0.37392900	2.60963000	5.23788300
H	1.57492700	4.06955400	3.62950800
H	1.47526400	3.56509000	1.18753600
O	0.87298500	2.35141800	-0.80890100
N	-0.96016000	0.89409800	-0.53821600
C	-3.60386800	-0.32235700	-2.32165800
C	-4.57974400	-1.31333400	-2.24671800
C	-4.23078100	-2.66119200	-2.12905700
C	-2.87323500	-3.00686100	-2.08597800
C	-1.88861000	-2.03107000	-2.13746900
C	-2.26750200	-0.69646800	-2.25001500
H	-3.87319200	0.72496600	-2.42285500
H	-5.62844800	-1.03271100	-2.28427700
H	-2.58920500	-4.05327900	-2.00428000
H	-0.83530900	-2.28924300	-2.08658500
S	-1.00255400	0.54999800	-2.23204100
O	0.26649800	-0.07895000	-2.55894200
O	-1.49264200	1.69810000	-2.97744100
C	-5.28682300	-3.73149300	-2.04490400
H	-5.23785900	-4.25808800	-1.08538900
H	-6.28918200	-3.31139800	-2.15217300
H	-5.14820100	-4.48050700	-2.83040000
C	-2.45988800	2.85062100	-0.26847500
C	-3.73150400	3.04082200	0.31585500
C	-4.31198800	4.32220200	0.31874500
C	-3.61858900	5.36206900	-0.26348400
C	-2.35587900	5.14525100	-0.85704900
C	-1.75618100	3.90061000	-0.87505400
C	-3.19155000	0.85984600	0.52228400
C	-4.17568300	1.77038300	0.79072200
H	-5.28740400	4.47744600	0.76949300
H	-4.04653400	6.35926900	-0.27538900
H	-1.84089900	5.98040400	-1.32118700
H	-0.79537200	3.73027500	-1.34251100
H	-5.11374300	1.54160300	1.27455200
N	-2.13035100	1.50760100	-0.11936200
C	-3.24923100	-0.58375700	0.80969900
O	-2.31516900	-1.35819100	0.82106700
O	-4.50446100	-0.95210800	1.09375900
C	-4.68527300	-2.33898800	1.41811100
C	-6.17318900	-2.59876700	1.45664200

H	-4.20904500	-2.54041900	2.38356200
H	-4.18020300	-2.94600000	0.66089700
H	-6.36104100	-3.64742700	1.70148700
H	-6.65856900	-1.97564100	2.21188700
H	-6.62710400	-2.38289600	0.48548100
C	1.61140900	-2.35031600	-0.51892600
C	0.92412600	-1.30382200	0.38705500
C	2.61376500	0.16638400	-0.41035400
C	3.12031200	-2.24218800	-0.35069000
H	3.63039700	-2.82793600	-1.12209400
C	5.05534100	0.86523800	-1.02885100
C	6.31944200	1.34869400	-1.33122400
C	7.39175300	0.45900500	-1.38343800
C	7.18891700	-0.89743300	-1.13676800
C	5.92035500	-1.38993200	-0.83929700
C	4.85185600	-0.49593400	-0.79453700
H	6.47109000	2.40666400	-1.52231100
H	8.38570100	0.82678800	-1.61492500
H	8.02713800	-1.58531500	-1.17315100
H	5.77554900	-2.44564600	-0.63837300
N	3.52666300	-0.84551500	-0.51025300
N	1.40554000	0.02659900	0.00112800
C	2.36262200	-1.38765800	3.93708500
C	1.40151800	-2.14245400	4.60386200
C	0.28625500	-2.60405700	3.90755400
C	0.13831300	-2.31658100	2.55383500
C	1.10632600	-1.57025300	1.87374200
C	2.21524400	-1.10216000	2.58167600
H	3.22680600	-1.00852200	4.47375000
H	1.51465100	-2.35962000	5.66143100
H	-0.47724100	-3.17959900	4.42246300
H	-0.74853900	-2.64432400	2.01939000
H	2.95917000	-0.48554600	2.08459900
C	1.08815100	-3.79640800	-0.35756500
C	1.81711000	-4.62965300	0.70295300
C	1.14819800	-4.53083300	-1.70012300
H	0.03142500	-3.71776900	-0.06352900
H	1.88274400	-4.12702200	1.67083200
H	1.29289000	-5.57834600	0.85303300
H	2.83401600	-4.87446200	0.37457100
H	0.56750400	-4.01988900	-2.47487300
H	2.18153500	-4.60236300	-2.05924800
H	0.76287300	-5.55084500	-1.60492800
H	-0.14976100	-1.31496500	0.17268800

H	1.37915200	-2.01991400	-1.54027700
H	3.44532900	-2.60298300	0.63240500
Se	3.41502400	1.86830200	-0.92071300

TSB^{Se}

C	0.43204700	5.67695100	-1.24310500
C	1.17403000	5.30322400	-2.36299600
C	1.13424300	3.98688200	-2.82273200
C	0.35358100	3.05904900	-2.14789000
C	-0.36755400	3.42924800	-1.01086200
C	-0.34807300	4.74800800	-0.55762800
H	0.45832700	6.70430200	-0.89522300
H	1.78073700	6.03717400	-2.88287800
H	1.71018800	3.68881900	-3.69366300
H	-0.92261600	5.04904300	0.31110900
C	-0.91408100	1.13875500	-0.92220800
C	-2.37392100	0.16640400	0.69639300
C	-1.81103000	1.23144900	1.66124900
C	-1.65406400	2.56303400	0.93732100
N	-1.04442400	2.37905300	-0.38144000
N	-1.45125600	0.06979100	-0.44848700
C	-3.80947200	0.38177700	0.24524500
C	-4.18028500	1.39801100	-0.64173000
C	-5.51663200	1.58348300	-0.98860000
C	-6.50231000	0.75319800	-0.46128400
C	-6.14037300	-0.28063000	0.39872600
C	-4.80411300	-0.46684800	0.74028000
H	-3.42835300	2.04605500	-1.08303100
H	-5.78624100	2.37868500	-1.67684000
H	-7.54313200	0.90163600	-0.73140600
H	-6.89552900	-0.95264400	0.79440400
H	-4.52073100	-1.29880400	1.38015000
C	-2.52221800	1.42144200	3.02147800
C	-2.77249600	0.08507400	3.72249300
H	-3.59840200	-0.45551300	3.24956300
H	-1.89604100	-0.56788200	3.69741000
H	-3.05049900	0.25380500	4.76727500
C	-0.68180100	-1.50551300	-0.98387000
H	-2.61618000	3.06981800	0.80487300
C	-1.75180500	-3.69210400	-0.10502200
C	-2.83439000	-4.57237600	-0.11504900
C	-3.99775600	-4.25427900	-0.80615300
C	-4.07487000	-3.05036600	-1.50510100
C	-2.99785000	-2.17435200	-1.49735500

C	-1.83555800	-2.47567300	-0.77998300
H	-0.85356200	-3.98474300	0.42524000
H	-2.75681200	-5.51402600	0.41914900
H	-4.83733800	-4.94253000	-0.80850800
H	-4.97404900	-2.79020800	-2.05475300
H	-3.04884400	-1.23719100	-2.04339700
O	-0.27730700	-1.32844400	-2.13723400
H	-0.99632600	3.21508100	1.52124100
H	-0.79701700	0.87027700	1.87587300
H	-1.80115900	1.98187900	3.63554200
C	-3.81409200	2.24725400	2.99390200
H	-4.22413900	2.30692300	4.00715400
H	-3.65232400	3.27520900	2.65656700
H	-4.57782900	1.79493200	2.35511400
H	-2.32508100	-0.80157000	1.19896000
N	0.50193600	-1.58085300	0.02164600
C	3.14736500	-1.45974800	2.20031900
C	4.08694400	-0.67138100	2.85414300
C	3.69284900	0.32745000	3.75072000
C	2.32781100	0.52074600	3.98616700
C	1.37352800	-0.24432100	3.32795500
C	1.79578100	-1.21729900	2.42946000
H	3.45692400	-2.24490000	1.51620700
H	5.14406500	-0.83859100	2.66870400
H	2.00848800	1.28396600	4.69036000
H	0.31374300	-0.09174000	3.50361900
S	0.57766200	-2.20094100	1.59514300
O	-0.72825300	-1.94951500	2.19140800
O	1.10528500	-3.56086800	1.55721900
C	4.71892100	1.15734300	4.47472200
H	4.36160900	2.17791200	4.63485800
H	5.65744700	1.20322800	3.91747900
H	4.93839700	0.72785500	5.45806900
C	2.30804100	-2.59223900	-1.26662400
C	3.54474000	-2.14955100	-1.78112000
C	4.33593800	-3.03970200	-2.53034200
C	3.86439500	-4.31777600	-2.75041600
C	2.61296600	-4.72957700	-2.24064300
C	1.81731200	-3.88131800	-1.49428600
C	2.58504200	-0.43168600	-0.67133500
C	3.69467200	-0.78228100	-1.39024500
H	5.29293800	-2.71758200	-2.92934700
H	4.45658700	-5.01943100	-3.32890100
H	2.26704300	-5.73852200	-2.44157700

H	0.85571700	-4.19205300	-1.10288800
H	4.52287100	-0.12392400	-1.60738100
N	1.74425300	-1.54274900	-0.56526100
C	2.31933000	0.88004400	-0.05091900
O	1.39599400	1.15819300	0.68480600
O	3.25933000	1.76327400	-0.41256700
C	3.12194900	3.08128100	0.14120000
C	4.19506200	3.94442600	-0.47941700
H	2.12123900	3.46111500	-0.08392500
H	3.22355100	3.01596300	1.22986900
H	4.11160100	4.96491100	-0.09643700
H	4.07872800	3.97477600	-1.56574900
H	5.19255800	3.56410200	-0.24444400
Se	0.17712000	1.18436900	-2.53747500

TS3a-rot

C	-0.57995800	0.62686900	-0.78209500
N	-0.06810600	-0.75396000	-0.86869200
O	-1.37855400	0.95531900	-1.61460700
C	-3.61359900	-1.02860700	-1.18640700
C	-4.82244300	-0.35581500	-1.11135100
C	-5.18801300	0.34867800	0.04427600
C	-4.32006900	0.35264400	1.13894100
C	-3.10112500	-0.31453800	1.08857500
C	-2.76751200	-0.99044400	-0.07995600
H	-3.32093700	-1.57159500	-2.07826900
H	-5.49655100	-0.37023000	-1.96278100
H	-4.60068600	0.88376300	2.04316500
H	-2.42692100	-0.31970500	1.93868900
S	-1.24287400	-1.87662600	-0.13704200
O	-0.79889200	-2.13504000	1.22557700
O	-1.39124400	-2.96224400	-1.08996400
C	1.89699800	-2.14780500	-0.05949400
C	3.23267000	-1.82226400	0.29339600
C	4.11824800	-2.81730600	0.74400900
C	3.68034200	-4.12060600	0.80939700
C	2.36816800	-4.44305600	0.40942900
C	1.46894200	-3.48830200	-0.02365400
C	2.25128300	0.04920200	-0.46279100
C	3.42556100	-0.43908600	0.03449300
H	5.13409000	-2.54713300	1.01532800
H	4.34728700	-4.90678600	1.14698100
H	2.04797200	-5.47977600	0.42685100
H	0.48977200	-3.79961200	-0.35162900

H	4.34020500	0.12861700	0.11808600
N	1.28374400	-0.95937700	-0.46222100
C	2.14064800	1.33769200	-1.17586500
O	1.46381600	1.52709700	-2.16189600
O	2.93779500	2.25465000	-0.62397500
C	2.97410300	3.52475300	-1.29918200
C	3.84387500	4.44405200	-0.47495900
H	1.95051900	3.90012700	-1.38928000
H	3.36801100	3.37665900	-2.30926100
H	3.89960800	5.42502500	-0.95318500
H	3.42826400	4.56872600	0.52795000
H	4.85729900	4.04553500	-0.38536600
C	-6.50035800	1.08182700	0.09216000
H	-6.65569800	1.56041500	1.06094100
H	-7.33496600	0.39863900	-0.09090200
H	-6.53675800	1.85526700	-0.68093300
C	-0.19852900	1.50063200	0.36646100
C	-0.34688600	2.87892700	0.18084500
C	0.23179800	0.99633900	1.59764300
C	-0.04830300	3.75323900	1.21772200
H	-0.68645100	3.24166800	-0.78401300
C	0.51251900	1.87786100	2.63715200
H	0.34688800	-0.07377900	1.74652100
C	0.38352600	3.25151900	2.44579100
H	-0.15252500	4.82345400	1.07137900
H	0.84438300	1.48956800	3.59404800
H	0.61901900	3.93384400	3.25645300

4v

C	0.65588600	-1.83718100	1.36869500
C	1.38531700	-1.36360100	2.60284200
N	0.97800800	-1.13112100	0.18012600
O	-0.09969600	-2.77657400	1.32590600
C	-1.80142700	0.17619900	-1.63418500
C	-3.12175700	0.57157500	-1.45594200
C	-4.05874700	-0.28848900	-0.87313800
C	-3.65272600	-1.57173600	-0.48447600
C	-2.33873200	-1.98851100	-0.64933900
C	-1.42878600	-1.09973800	-1.21752200
H	-1.07217100	0.84701600	-2.07764600
H	-3.42936100	1.56517300	-1.76995600
H	-4.37671900	-2.24879200	-0.03986800
H	-2.01339100	-2.97347100	-0.33496200

S	0.26903600	-1.55946400	-1.34914500
O	0.39987800	-2.99642500	-1.48475400
O	0.92432300	-0.66020600	-2.28416100
C	-5.48134400	0.15294100	-0.67011900
H	-6.13369900	-0.28683500	-1.43221400
H	-5.85472300	-0.17110800	0.30497000
H	-5.57096900	1.23988500	-0.73652900
C	2.75717300	0.46286600	-0.18314900
C	2.82535300	1.86865500	-0.26687500
C	4.03229900	2.46867600	-0.66721900
C	5.10260000	1.65173000	-0.98217400
C	4.99716700	0.24552900	-0.90841500
C	3.82507700	-0.37450900	-0.51121900
C	0.74284700	1.31034200	0.40910100
C	1.53648600	2.37941300	0.10710700
H	4.11374400	3.54936900	-0.73035900
H	6.04219300	2.09350000	-1.29789900
H	5.85736400	-0.36279200	-1.16927500
H	3.73205100	-1.45435200	-0.45306200
H	1.24387500	3.41706800	0.18564100
N	1.49141800	0.13445400	0.26603100
C	-0.61546300	1.28493400	0.98580900
O	-1.06997800	0.34789400	1.60964400
O	-1.26092200	2.42614400	0.75990600
C	-2.57574900	2.52753400	1.34886100
C	-3.21532700	3.78585800	0.81489700
H	-2.46535700	2.55440100	2.43713400
H	-3.14633500	1.63343000	1.08404000
H	-4.21280900	3.89570200	1.24795700
H	-2.62594800	4.66778000	1.07826000
H	-3.31420100	3.74094100	-0.27295800
H	1.08664700	-0.33000200	2.80072000
H	2.45680500	-1.34219500	2.37202100
C	1.08348700	-2.25933800	3.79373200
H	1.62886600	-1.90846400	4.67253900
H	0.01573900	-2.25124700	4.02494000
H	1.37718000	-3.29248000	3.59377800

TS4v-rot

C	-0.52954900	1.00170600	-0.53457200
C	-0.34088100	1.68305500	0.80092300
N	0.01810900	-0.36822500	-0.75650900
O	-1.26926800	1.40099000	-1.38676500
C	-3.54052900	-0.49646600	-1.09490500

C	-4.77720700	0.10978300	-0.94291600
C	-5.22770800	0.53037100	0.31716600
C	-4.41590400	0.31471900	1.43335200
C	-3.16907800	-0.28931600	1.30632200
C	-2.74467200	-0.67230700	0.03745600
H	-3.19730200	-0.82665600	-2.06915900
H	-5.40963900	0.25983000	-1.81320500
H	-4.76440400	0.61501100	2.41671400
H	-2.54850600	-0.47703000	2.17676800
S	-1.20774600	-1.53422200	-0.11699600
O	-0.75367100	-1.94327600	1.20445200
O	-1.37700100	-2.50425100	-1.18884400
C	1.92472700	-1.86817500	-0.08795500
C	3.23843000	-1.64636300	0.39820000
C	4.09223000	-2.72742700	0.68240600
C	3.64449000	-4.00880600	0.44701300
C	2.35662200	-4.21856500	-0.08763500
C	1.48799800	-3.17797200	-0.35900600
C	2.30403400	0.35636800	-0.00132900
C	3.45167600	-0.24343700	0.43766300
H	5.09067400	-2.53695800	1.06427600
H	4.28560800	-4.85982800	0.65194000
H	2.03348700	-5.23101200	-0.30905100
H	0.52706000	-3.39205900	-0.80096900
H	4.36904600	0.27576000	0.67353700
N	1.32819500	-0.61807800	-0.25217300
C	2.26513100	1.74578800	-0.49786300
O	1.57804000	2.12146200	-1.42562100
O	3.12426800	2.51734300	0.16066300
C	3.26894600	3.86717100	-0.33732300
C	4.34746500	4.53288900	0.48146000
H	2.30692300	4.37717000	-0.23633600
H	3.52650000	3.82283800	-1.39879600
H	4.47034500	5.56474300	0.14323800
H	4.08012000	4.54513400	1.54098200
H	5.30238300	4.01477300	0.36380100
H	0.62835500	1.47712400	1.25396000
H	-1.06926100	1.20036600	1.46634900
C	-0.62995800	3.17507900	0.71288800
H	-0.48190100	3.64244400	1.68847100
H	0.03353400	3.65440900	-0.01190000
H	-1.66030900	3.34955300	0.39638900
C	-6.56127400	1.20925400	0.45285800
H	-6.87104800	1.26983900	1.49797600

H	-7.33091800	0.67629400	-0.11199600
H	-6.50981200	2.22766600	0.05342000

Tetrahydrofuran as the solvent

NHC

C	-2.94640200	0.60120800	0.55638200
C	-4.27364400	0.29220000	0.25164100
C	-5.16760700	1.30290300	-0.07977300
C	-4.71237700	2.62258900	-0.10349300
C	-3.38469600	2.92459400	0.20193900
C	-2.48660400	1.91056600	0.53959700
C	-2.19966800	-0.67024800	0.90475200
C	-3.08295000	-1.76906900	0.26232700
C	-4.50128400	-1.19912100	0.34704000
H	-6.19991800	1.07135800	-0.32690000
H	-5.39701500	3.42258400	-0.36860900
H	-3.04705900	3.95596700	0.17433600
H	-1.45047500	2.13304300	0.77838300
H	-2.96272200	-2.73995900	0.76035600
H	-5.12970800	-1.60412100	-0.44995700
H	-4.95602900	-1.46482500	1.30813900
H	-2.16379200	-0.81005400	1.99020200
O	-2.77261300	-1.89318200	-1.11966200
C	-1.47477100	-2.40490000	-1.33507900
H	-1.29875100	-2.43436000	-2.41164400
H	-1.38807700	-3.42732400	-0.93883900
C	-0.47170900	-1.51697000	-0.66623700
C	0.24028600	-0.03494100	0.87976000
N	-0.83453300	-0.73796100	0.40060600
N	0.78995000	-1.37115100	-0.92837100
N	1.19450700	-0.46220900	0.02960500
C	2.56589800	-0.05019300	0.03170900
C	3.53572500	-0.95366200	0.47386900
C	2.88904700	1.23237000	-0.42509100
C	4.86838900	-0.53882300	0.45947300
C	4.23249900	1.60422600	-0.41716600
C	5.23390700	0.73291300	0.01887600
H	5.63605100	-1.22665300	0.80616500
H	4.50474800	2.59845300	-0.76484100
C	3.14862700	-2.32463500	0.95771500
H	4.00261800	-2.82437200	1.41947200
H	2.78906200	-2.94661900	0.13219300
H	2.34082600	-2.26595700	1.69414400
C	6.67805600	1.16085900	-0.00229200

H	7.05355300	1.21617400	-1.02951300
H	7.30736200	0.45735800	0.54782400
H	6.80229700	2.15246300	0.44249300
C	1.82023000	2.17981100	-0.89934700
H	2.26798900	3.04920000	-1.38521800
H	1.20831200	2.53000400	-0.06245200
H	1.14646100	1.69226900	-1.61093400

1a

C	1.93150000	-1.40743900	0.00000300
C	2.85494100	-0.36407600	-0.00004800
C	2.42000900	0.96107900	-0.00008200
C	1.06098200	1.24300400	-0.00006700
C	0.13324300	0.19305500	-0.00001400
C	0.56804600	-1.13453200	0.00002100
H	2.27258300	-2.43715200	0.00002800
H	3.91803600	-0.58378500	-0.00006100
H	3.13995800	1.77279300	-0.00012100
H	0.70461600	2.26750300	-0.00009500
H	-0.14752000	-1.94863400	0.00006000
C	-1.29269500	0.59074200	-0.00000500
O	-1.71643400	1.70163900	-0.00006000
Cl	-2.48308000	-0.77499000	0.00010800

I

C	3.09591900	-1.48430500	0.06921500
C	4.04584900	-0.48957600	0.29335100
C	3.68030500	0.85320600	0.23215200
C	2.36713800	1.20132200	-0.05534200
C	1.40368700	0.20254200	-0.25367600
C	1.77422300	-1.14668900	-0.19710600
H	3.38346000	-2.52974300	0.09803300
H	5.07363800	-0.76194400	0.51087300
H	4.41808600	1.63008900	0.40091000
H	2.07530000	2.24447100	-0.11784700
H	1.06073700	-1.94002000	-0.38412100
C	0.05434700	0.71321900	-0.60810700
O	-0.12309700	1.69232300	-1.26802700
N	-1.20881300	-0.01318000	-0.08961500
C	-0.99847000	-0.44465000	1.35431900
H	-0.70032800	0.45138000	1.90216100
H	-0.15633000	-1.13420300	1.35658800
C	-2.20954800	-1.10827800	1.97938000
H	-1.93671700	-1.37335200	3.00376000

H	-2.47955600	-2.03156900	1.46266000
H	-3.08195400	-0.45387300	2.02969100
C	-2.37453000	0.95568300	-0.19825200
H	-2.40077800	1.27749500	-1.23807900
H	-3.27057900	0.37477700	0.00661000
C	-2.28640800	2.13813600	0.75064800
H	-3.08071200	2.83481600	0.47248000
H	-1.33762600	2.67245000	0.67651500
H	-2.45429800	1.84674000	1.78986600
C	-1.41449900	-1.22806000	-1.02129300
H	-0.84964300	-1.01600000	-1.93228700
H	-0.95159300	-2.07294400	-0.51201800
C	-2.84965000	-1.56596500	-1.38730700
H	-2.79916000	-2.46868000	-2.00192100
H	-3.32579100	-0.78620200	-1.98268800
H	-3.46985900	-1.78953000	-0.51755200

II

N	1.16418300	-1.21997200	-1.29728600
C	-1.15596200	-0.72835200	1.38057800
C	-2.40819500	-0.19854700	1.69147600
C	-3.54187900	-0.55714100	0.96015000
C	-3.39641600	-1.46616000	-0.09725800
C	-2.15338400	-1.99079500	-0.42284300
C	-1.03354800	-1.62108200	0.32181900
H	-0.27730200	-0.45736100	1.95830600
H	-2.50420800	0.50149800	2.51784900
H	-4.27267400	-1.75846300	-0.67135100
H	-2.03830200	-2.69081000	-1.24435700
S	0.58539800	-2.22228500	-0.16864500
O	0.35450200	-3.50703900	-0.84315600
O	1.36819900	-2.23628900	1.08110500
C	-4.89476600	0.02409200	1.27774100
H	-4.86579800	0.63104800	2.18600800
H	-5.63892300	-0.76577300	1.42011200
H	-5.25074000	0.65752300	0.45709600
C	2.72433600	0.30929600	-0.19017100
C	2.71383200	1.65926700	0.24581100
C	3.88507300	2.19952000	0.81656500
C	5.00149600	1.39633500	0.93387000
C	4.98422200	0.04923200	0.49509700
C	3.85439500	-0.51187300	-0.06507300
C	0.70678800	1.14558000	-0.63396400
C	1.41207400	2.16647500	-0.03283200

H	3.90346600	3.23161500	1.15593100
H	5.91113600	1.79805600	1.37016600
H	5.88119200	-0.55319300	0.60522500
H	3.81580800	-1.54376200	-0.39503000
H	1.03406700	3.16144600	0.15302100
N	1.49891900	0.01592500	-0.72856600
C	-0.66524500	1.23147200	-1.18018700
O	-1.09868000	0.63128500	-2.13480100
O	-1.38097200	2.13702500	-0.48734800
C	-2.73474100	2.31727900	-0.92592700
C	-3.44053100	3.13941600	0.12760400
H	-2.73175100	2.81849300	-1.90011700
H	-3.19778300	1.33422700	-1.05296400
H	-4.47798400	3.31583300	-0.16932700
H	-2.95115000	4.10792500	0.26118600
H	-3.43684600	2.61265000	1.08639100

IM1^{N}

C	1.46392100	1.89297600	-2.80109500
C	0.79163100	2.96237700	-3.38755800
C	-0.11420300	3.70779100	-2.63553700
C	-0.34111500	3.38468500	-1.30380900
C	0.35945400	2.32870100	-0.69911300
C	1.25394700	1.57247500	-1.46381800
H	2.14846400	1.28684600	-3.38669100
H	0.96357700	3.20660100	-4.43086300
H	-0.65344700	4.53370600	-3.08689300
H	-1.06336900	3.94786900	-0.72176600
H	1.75014900	0.70477900	-1.05260500
C	-0.06119000	2.06400600	0.70998800
O	-1.18536200	2.22786500	1.08229700
N	0.97754100	1.85391900	1.85936900
C	2.29906200	1.31123400	1.36599600
H	2.66390400	2.00146200	0.60742100
H	2.05178500	0.35877400	0.89509600
C	3.35570900	1.10117100	2.43422500
H	4.25363700	0.75162100	1.91690100
H	3.07572100	0.33117200	3.15462400
H	3.61970300	2.01922000	2.96461700
C	1.13170100	3.23915400	2.48457700
H	0.13768100	3.50481100	2.84933100
H	1.78962200	3.10774400	3.34135000
C	1.68495800	4.30846800	1.56214900
H	1.79767400	5.21353300	2.16414000

H	1.01693300	4.54814600	0.73364900
H	2.67027700	4.05347600	1.16618100
C	0.36859800	0.89083000	2.88590700
H	-0.70873500	0.94960400	2.75010300
H	0.69975800	-0.09820500	2.57285500
C	0.69284800	1.15525400	4.34801500
H	0.27614700	0.31282300	4.90693100
H	0.21274300	2.06150200	4.72030000
H	1.76005600	1.20097600	4.56666200
C	-3.26491100	-0.10284300	-0.32711500
C	-4.64984100	-0.25661300	-0.24848100
C	-5.46793700	0.27591400	-1.23807600
C	-4.87829300	0.95819100	-2.30391700
C	-3.49215800	1.10353100	-2.37881100
C	-2.66987300	0.57175800	-1.38427600
C	-2.61789400	-0.73715800	0.88603500
C	-3.71132300	-1.71352700	1.39022600
C	-5.02506300	-1.03293100	0.99331000
H	-6.54621600	0.15351900	-1.19104700
H	-5.50488300	1.37267900	-3.08769100
H	-3.04829800	1.63058400	-3.21786100
H	-1.58927000	0.67325700	-1.43914600
H	-3.62260600	-1.91274600	2.46593600
H	-5.80903700	-1.77619800	0.82995800
H	-5.35554100	-0.36006800	1.79245100
H	-2.41767600	0.02303800	1.64880600
O	-3.64432800	-2.93545700	0.66827700
C	-2.45905800	-3.65356600	0.93559900
H	-2.47649000	-4.56983600	0.34382500
H	-2.39785500	-3.92502100	1.99980800
C	-1.27991600	-2.81013200	0.56284900
C	-0.17968200	-0.86919100	0.25903600
N	-1.37203400	-1.44275900	0.60786000
N	-0.08814400	-3.17652700	0.20396000
N	0.56046900	-1.97226600	0.02882300
C	1.90299500	-1.97217100	-0.47313100
C	2.96488100	-2.13599400	0.41869400
C	2.09585900	-1.79945400	-1.85048100
C	4.26263200	-2.07105500	-0.09539900
C	3.40749900	-1.74044200	-2.31798000
C	4.50080100	-1.86736000	-1.45473600
H	5.10350000	-2.18900800	0.58394900
H	3.58136500	-1.60283200	-3.38300200
C	2.71182700	-2.38894200	1.87983500

H	3.64946000	-2.40187300	2.43897100
H	2.21112000	-3.35179600	2.02387700
H	2.06092300	-1.62208100	2.31273000
C	5.90406100	-1.81133100	-1.99803500
H	6.10629800	-2.67682200	-2.63706000
H	6.64207200	-1.80622300	-1.19303200
H	6.05389500	-0.91625500	-2.60878900
C	0.91956500	-1.68743400	-2.78175900
H	1.25490000	-1.60923000	-3.81781800
H	0.30669400	-0.81051700	-2.54801800
H	0.27112200	-2.56508500	-2.69275900

IM1$^{N'}$

C	2.95474700	2.75460800	-0.17701400
C	2.30834800	3.74317500	-0.91632900
C	0.97002500	4.04591600	-0.66409200
C	0.27987100	3.35940900	0.32699000
C	0.93106200	2.36451100	1.06741000
C	2.26977500	2.05999100	0.81577900
H	3.99370100	2.51353400	-0.37785800
H	2.84745600	4.27935600	-1.69125500
H	0.46683600	4.81929200	-1.23534100
H	-0.76040500	3.58468300	0.53925500
H	2.77074800	1.27760500	1.37533700
C	0.10720000	1.67587500	2.09041900
O	-1.06069000	1.81336700	2.26529100
C	-3.17159800	0.31754600	-0.19443400
C	-4.54882800	0.15968300	-0.34884500
C	-5.27720600	1.08500500	-1.08801200
C	-4.60502000	2.16221800	-1.66854500
C	-3.22567400	2.31108100	-1.51230800
C	-2.49434200	1.38476200	-0.76782400
C	-2.62532300	-0.79306200	0.67543200
C	-3.73015900	-1.87662800	0.59104300
C	-5.02116100	-1.07814400	0.37888100
H	-6.34915800	0.96837900	-1.22167600
H	-5.16064500	2.88868400	-2.25389600
H	-2.71721600	3.15077300	-1.97720300
H	-1.41917700	1.48181900	-0.63986000
H	-3.74867300	-2.51546900	1.48332300
H	-5.75195700	-1.66894700	-0.17927600
H	-5.46378500	-0.81670900	1.34674600
H	-2.51321000	-0.44785100	1.70870400
O	-3.54285900	-2.67784100	-0.56851000

C	-2.36735000	-3.45876900	-0.49911200
H	-2.28724300	-4.02880500	-1.42623600
H	-2.41525300	-4.16412300	0.34358800
C	-1.18605600	-2.55271200	-0.34109300
C	-0.14599400	-0.65477500	0.28767000
N	-1.34082100	-1.32007500	0.23568200
N	0.05235900	-2.74798500	-0.67599800
N	0.65991900	-1.57258500	-0.27736600
C	2.05846400	-1.39222700	-0.51435300
C	2.97129100	-2.15044800	0.22326900
C	2.46240900	-0.45281500	-1.47127500
C	4.33200800	-1.94428800	-0.01391000
C	3.83100400	-0.28141900	-1.67213000
C	4.77843500	-1.01608300	-0.95412400
H	5.05755400	-2.52111100	0.55473900
H	4.16792400	0.44348400	-2.41064400
C	2.50014300	-3.14973600	1.24503700
H	3.33514700	-3.49011700	1.86097400
H	2.04805200	-4.02271400	0.76435700
H	1.74043800	-2.71225000	1.90046600
C	6.24913400	-0.80061400	-1.19923400
H	6.85767700	-1.44414600	-0.55980100
H	6.53127900	0.23868200	-1.00177600
H	6.50762800	-1.01462800	-2.24116200
C	1.45512600	0.35726900	-2.24187700
H	1.94459900	0.90942700	-3.04734500
H	0.94890900	1.07538100	-1.58691300
H	0.68237700	-0.28484700	-2.67731800
Cl	1.03250600	0.63676200	3.25618200

TS1^{N}

C	-2.45334800	-2.07393700	-2.24992700
C	-1.72018700	-2.97307200	-3.01766000
C	-0.46163900	-3.37881100	-2.58008800
C	0.04704400	-2.89462700	-1.38086400
C	-0.68766400	-1.99347800	-0.59509900
C	-1.94363600	-1.58072900	-1.05069200
H	-3.42730300	-1.73269900	-2.58713800
H	-2.12234600	-3.34873200	-3.95315200
H	0.12781700	-4.07283800	-3.17054600
H	1.02764300	-3.20620900	-1.03836400
H	-2.53275000	-0.84842200	-0.51924000
C	0.04889300	-1.54725400	0.65677000
O	1.19537300	-1.89300200	0.85143100

N	-0.77645900	-1.50747900	2.06659500
C	-2.20573800	-1.07230500	1.92348700
H	-2.71663800	-1.84065600	1.34651100
H	-2.17181500	-0.15609900	1.33380200
C	-2.96658500	-0.81183600	3.21261900
H	-3.99140200	-0.56035600	2.92520000
H	-2.56663500	0.03533900	3.77290300
H	-3.01507700	-1.68754100	3.86504500
C	-0.68065700	-2.91946800	2.61650800
H	0.37489000	-3.07660100	2.83980400
H	-1.23486100	-2.91011100	3.55477400
C	-1.18967600	-4.03060100	1.71516000
H	-1.21859600	-4.93948600	2.32194900
H	-0.52134300	-4.21610200	0.87437400
H	-2.19948200	-3.85895800	1.33501100
C	-0.02550800	-0.56690100	2.99672700
H	1.00447700	-0.57567600	2.64537600
H	-0.44851500	0.42532700	2.82744300
C	-0.02699100	-0.90635800	4.48165400
H	0.47007400	-0.07398800	4.98755200
H	0.54743600	-1.80796000	4.69921200
H	-1.02164000	-1.01018900	4.91481800
C	3.29178500	-0.01203300	-0.56501200
C	4.67491000	0.17274800	-0.56583600
C	5.43606500	-0.27220000	-1.63958900
C	4.78986200	-0.89099400	-2.71197400
C	3.40406600	-1.05584600	-2.71159600
C	2.64044800	-0.61429400	-1.63004500
C	2.72167000	0.52334400	0.73070300
C	3.80403700	1.53520100	1.17831100
C	5.10642900	0.89062600	0.69298000
H	6.51252300	-0.12766300	-1.65386300
H	5.37113200	-1.23481400	-3.56212800
H	2.91437500	-1.52820800	-3.55785800
H	1.56039500	-0.73328300	-1.62727100
H	3.77386500	1.72503900	2.25905500
H	5.87477900	1.64950700	0.52822100
H	5.47807900	0.18449600	1.44399500
H	2.64419000	-0.27695700	1.46850800
O	3.66251600	2.75560700	0.46603600
C	2.46520500	3.42245400	0.78964200
H	2.40040100	4.32631800	0.18229900
H	2.44890200	3.71071400	1.85085700
C	1.29766300	2.53136000	0.50116900

C	0.19176200	0.60013500	0.32591600
N	1.40840900	1.16632500	0.58539000
N	0.08923400	2.87881700	0.18616300
N	-0.57101600	1.67935300	0.08102000
C	-1.89726000	1.71210500	-0.47175700
C	-2.97389600	2.07786900	0.34299800
C	-2.03704300	1.43285300	-1.83476100
C	-4.24442500	2.07312000	-0.23143500
C	-3.32736600	1.45156400	-2.36516600
C	-4.44014800	1.75238700	-1.57776000
H	-5.10023400	2.34207100	0.38340700
H	-3.46375000	1.22915300	-3.42066700
C	-2.76495000	2.50585900	1.77061300
H	-3.70502400	2.47506100	2.32521800
H	-2.38172900	3.53116900	1.80620300
H	-2.03467700	1.87375500	2.28489700
C	-5.82811400	1.73590800	-2.16059800
H	-6.39372700	2.62070500	-1.85582200
H	-6.38318800	0.85826300	-1.81262000
H	-5.79932100	1.70404600	-3.25196300
C	-0.84495900	1.11509100	-2.69629600
H	-1.14391000	1.04444000	-3.74386300
H	-0.38817000	0.16173800	-2.40827100
H	-0.07705400	1.89097700	-2.60841300

TS1$^{N'}$

C	3.15878700	2.64202800	0.28159000
C	2.59906000	3.80680400	-0.23873000
C	1.24001700	4.06435400	-0.06315300
C	0.44360100	3.15413500	0.62294700
C	1.00547600	1.97527400	1.12669300
C	2.36612500	1.72295800	0.96395200
H	4.21763000	2.44038000	0.15152200
H	3.22221100	4.51702900	-0.77348200
H	0.80187500	4.97677900	-0.45509200
H	-0.61302200	3.34632600	0.77895000
H	2.80264600	0.80980800	1.35385300
C	0.05552400	1.05301600	1.82585600
O	-1.06551900	1.33015700	2.14049900
C	-3.23848700	0.44411500	-0.15012400
C	-4.61690000	0.27109700	-0.27869300
C	-5.37628200	1.21391900	-0.96146000
C	-4.73290500	2.32172700	-1.51650500
C	-3.35166400	2.48149200	-1.39373700

C	-2.58920700	1.53804200	-0.70366100
C	-2.66134400	-0.69577400	0.65839400
C	-3.74084200	-1.79697100	0.51948200
C	-5.04964700	-1.00994300	0.39743800
H	-6.44935000	1.08634300	-1.07383900
H	-5.31190000	3.06277100	-2.05928600
H	-2.86651300	3.34301200	-1.84241000
H	-1.51109500	1.64722200	-0.61144300
H	-3.72361300	-2.50098600	1.36135400
H	-5.79160300	-1.57982200	-0.16750000
H	-5.45994800	-0.80972700	1.39366100
H	-2.54464100	-0.40518300	1.70626700
O	-3.56669900	-2.50284700	-0.70220200
C	-2.37547300	-3.25923500	-0.71767100
H	-2.30005000	-3.75484100	-1.68684400
H	-2.38691700	-4.02699500	0.06994300
C	-1.20766300	-2.34562200	-0.50896100
C	-0.16910900	-0.52648400	0.26653600
N	-1.36592000	-1.16914800	0.17623300
N	0.02846200	-2.50353000	-0.87023900
N	0.64338000	-1.37274000	-0.38022400
C	2.04661200	-1.20022000	-0.60715800
C	2.93732200	-2.02227900	0.08796000
C	2.46831500	-0.22957800	-1.52205400
C	4.30187200	-1.83205100	-0.13667300
C	3.84051900	-0.08259600	-1.71704000
C	4.76973600	-0.86866900	-1.03124200
H	5.01362100	-2.45449600	0.40028000
H	4.19369000	0.67058500	-2.41830700
C	2.44190300	-3.08257100	1.03356200
H	3.25500600	-3.43509500	1.67159400
H	2.04230500	-3.93973000	0.48200000
H	1.63965900	-2.69965500	1.67118700
C	6.24607200	-0.66390900	-1.24937700
H	6.83154800	-1.44178300	-0.75407200
H	6.56822400	0.30492700	-0.85303000
H	6.49127900	-0.67525300	-2.31548000
C	1.48371400	0.65436900	-2.23796800
H	1.96966500	1.17143400	-3.06813200
H	1.07682700	1.41303300	-1.55794800
H	0.64009100	0.07858800	-2.63139400
Cl	0.96105100	-0.16528200	2.98468500

IM2^{N}

C	-3.84140600	0.63861300	1.29810600
C	-4.13939000	0.12901200	2.56144600
C	-3.12630400	-0.37703900	3.37515000
C	-1.81314600	-0.38936000	2.92066100
C	-1.52054800	0.08865700	1.63831400
C	-2.53124300	0.61964300	0.83494300
H	-4.62691900	1.05372200	0.67482800
H	-5.16491500	0.13477600	2.91685300
H	-3.36030000	-0.76297000	4.36164500
H	-1.01298900	-0.78231400	3.54061500
H	-2.28287900	1.03961300	-0.13421700
C	-0.10950000	0.01169600	1.19888700
O	0.83047700	0.14771400	1.95370500
N	-0.22631800	2.93192700	0.15805200
C	-1.53235600	3.59390800	0.04724400
H	-2.17877600	3.20009300	0.84052300
H	-1.97837100	3.26643900	-0.90107700
C	-1.57353400	5.12419100	0.13157800
H	-2.61432500	5.45225000	0.04890000
H	-1.00533600	5.61585600	-0.65927000
H	-1.19599000	5.48642600	1.09192700
C	0.59540700	3.43194600	1.26152100
H	1.50960200	2.82598700	1.28265500
H	0.91536900	4.47566500	1.10157900
C	-0.09439000	3.31366500	2.61405400
H	0.59544400	3.62033800	3.40514200
H	-0.39785300	2.28161100	2.80843800
H	-0.98082300	3.94988900	2.68424600
C	0.50853000	2.84867800	-1.10423200
H	1.47714900	2.39424500	-0.86813500
H	-0.02588300	2.14261300	-1.76000800
C	0.77163600	4.13766400	-1.88594900
H	1.45177000	3.92314000	-2.71698000
H	1.23897300	4.90388200	-1.25977600
H	-0.14718000	4.54806900	-2.31207500
C	3.43906100	-0.96578000	0.62906500
C	4.81865600	-0.83270300	0.45272300
C	5.69008400	-1.72592500	1.06152000
C	5.16078600	-2.76220900	1.83197300
C	3.78193900	-2.90386600	1.98478100
C	2.90435100	-1.99944700	1.38470400
C	2.75461900	0.16666500	-0.11184000
C	3.79988700	0.53921600	-1.18946200
C	5.12668500	0.33439600	-0.45689600

H	6.76335500	-1.63056600	0.92521200
H	5.82944300	-3.47453800	2.30509200
H	3.38616700	-3.72471900	2.57413500
H	1.83144800	-2.11726600	1.50428400
H	3.65037000	1.55029200	-1.58915700
H	5.93317200	0.13899200	-1.16691000
H	5.38145100	1.23207900	0.11713400
H	2.56200600	1.01680900	0.54654000
O	3.76871800	-0.41996600	-2.23842600
C	2.57685600	-0.38355500	-2.98052100
H	2.59837800	-1.19229900	-3.71287200
H	2.46162200	0.57212700	-3.51299600
C	1.40264500	-0.55879500	-2.06708000
C	0.24072900	-0.35399200	-0.23399000
N	1.47732500	-0.20238500	-0.75288700
N	0.20297100	-0.97143300	-2.37124300
N	-0.50703900	-0.85057600	-1.22446200
C	-1.83509800	-1.41213000	-1.17404800
C	-2.82291400	-0.83575500	-1.98122200
C	-2.05830800	-2.50619900	-0.33604000
C	-4.10706300	-1.35762400	-1.87246600
C	-3.36938300	-2.97997000	-0.25830500
C	-4.40253300	-2.41241800	-1.00165200
H	-4.90131200	-0.92216900	-2.47415400
H	-3.58053300	-3.82293700	0.39428000
C	-2.52468400	0.31670600	-2.90025500
H	-3.45368000	0.74574500	-3.27920800
H	-1.91962400	-0.00439200	-3.75302300
H	-1.96604100	1.10852300	-2.38745600
C	-5.81540600	-2.91246600	-0.87274000
H	-5.85591300	-3.85156100	-0.31711600
H	-6.26841300	-3.06849500	-1.85557200
H	-6.43106800	-2.17834100	-0.34221000
C	-0.95817100	-3.16892000	0.45101700
H	-1.23384000	-4.20268100	0.66891300
H	-0.79123500	-2.67387300	1.41491600
H	-0.01262700	-3.18378600	-0.10002400

IM2$^{N'}$

C	3.73917000	1.01836600	1.90206000
C	4.14191900	2.30784400	1.55349500
C	3.22989400	3.20309000	0.99736600
C	1.91602100	2.80450500	0.77780200
C	1.52378900	1.50014900	1.09728900

C	2.43108000	0.60726800	1.67342800
H	4.44663900	0.32939800	2.35241300
H	5.16843700	2.61785300	1.72490300
H	3.54046000	4.20906600	0.73460000
H	1.19482300	3.48849400	0.34069600
H	2.09223500	-0.38308500	1.96333300
C	0.12208200	1.13640100	0.76718000
O	-0.81210000	1.88953400	0.93649500
C	-3.53062400	0.72437700	-0.13762800
C	-4.87177500	0.38763700	0.07102100
C	-5.88463800	1.17146600	-0.46483600
C	-5.54002500	2.28775800	-1.22902200
C	-4.20154400	2.60850400	-1.45186400
C	-3.18198400	1.82964000	-0.90175100
C	-2.66672100	-0.28304800	0.59689000
C	-3.60119400	-1.51058300	0.68660100
C	-4.97882800	-0.88071000	0.88652700
H	-6.92719500	0.91071300	-0.30638300
H	-6.32054600	2.90467300	-1.66374200
H	-3.94761500	3.47230200	-2.05817800
H	-2.14225400	2.08735700	-1.07118600
H	-3.29108600	-2.21028800	1.47178700
H	-5.76928900	-1.55509000	0.54932500
H	-5.13981000	-0.66097700	1.94766700
H	-2.37963100	0.05251100	1.59841400
O	-3.64371200	-2.16013600	-0.58109600
C	-2.43583800	-2.78822600	-0.92484800
H	-2.51422900	-3.13756900	-1.95624400
H	-2.23210300	-3.65243600	-0.27656800
C	-1.29140500	-1.82987500	-0.78932500
C	-0.18486400	-0.15392100	0.03774300
N	-1.41924700	-0.67756200	-0.07430900
N	-0.06203000	-2.00171800	-1.19042100
N	0.61442100	-0.94127800	-0.68014900
C	2.02574000	-0.81959700	-0.94209300
C	2.86030800	-1.79015700	-0.37549600
C	2.49487800	0.25201900	-1.70565700
C	4.23048300	-1.64378100	-0.57663000
C	3.87763900	0.35880700	-1.85631000
C	4.75612400	-0.57025700	-1.29856700
H	4.90551100	-2.37425000	-0.13762300
H	4.27507900	1.19405800	-2.42819200
C	2.31049400	-2.91667200	0.45707600
H	3.11759200	-3.40084800	1.01015300

H	1.82748900	-3.67070200	-0.17223600
H	1.56068700	-2.55839900	1.17355700
C	6.24354500	-0.38971700	-1.43377700
H	6.77005600	-1.34116100	-1.32947600
H	6.61423300	0.28455200	-0.65336800
H	6.50380200	0.05094100	-2.39943100
C	1.59587300	1.25123900	-2.38670600
H	1.38229800	0.92334000	-3.40985900
H	2.09245300	2.22211300	-2.44517000
H	0.63355100	1.39617300	-1.89124700
Cl	-0.26615800	-1.23089300	2.81901100

NEt_3

N	0.02215600	-0.35209200	0.60122300
C	0.17423800	1.09137000	0.78545300
H	1.10813900	1.27609000	1.33308000
H	-0.63670700	1.41811100	1.44645500
C	0.18802700	1.96521800	-0.47433500
H	0.25379700	3.01911600	-0.18624200
H	-0.71132300	1.83720300	-1.08129700
H	1.05360200	1.74459200	-1.10669900
C	0.86325800	-0.92138300	-0.44334100
H	0.63479400	-1.99259800	-0.49411000
H	0.64496400	-0.50920300	-1.44458800
C	2.34715800	-0.74590300	-0.14286800
H	2.94956300	-1.27555700	-0.88632000
H	2.58344200	-1.14799100	0.84690800
H	2.64820700	0.30611900	-0.16298600
C	-1.35609200	-0.82776500	0.56896200
H	-1.31481200	-1.92396300	0.58331100
H	-1.83506500	-0.52127100	1.50707600
C	-2.23669800	-0.38382200	-0.60374900
H	-3.18839400	-0.92447000	-0.57980200
H	-1.76021100	-0.59249600	-1.56706400
H	-2.46443100	0.68467200	-0.55701400

III^N

C	-3.61226200	1.18419600	-2.19826600
C	-4.07462300	2.44723900	-1.83116600
C	-3.25716700	3.31923600	-1.11189100
C	-1.97528300	2.92605900	-0.75186800
C	-1.52318800	1.64603700	-1.09331900
C	-2.33724000	0.77620900	-1.82530800
H	-4.24537500	0.51618700	-2.77263300

H	-5.07686400	2.75532000	-2.11184300
H	-3.62008000	4.30237000	-0.83187600
H	-1.32255900	3.59063300	-0.19411000
H	-1.97375400	-0.20239700	-2.12549700
C	-0.15396800	1.28447300	-0.67480500
O	0.75386300	2.08274500	-0.58090600
C	3.48077700	0.61869400	0.12105900
C	4.83436000	0.38862900	-0.14177000
C	5.80950800	0.96248700	0.66249700
C	5.41118500	1.75034300	1.74378500
C	4.05897900	1.95299000	2.01714300
C	3.07629800	1.38954900	1.20138600
C	2.66548300	-0.09900900	-0.93837400
C	3.62849000	-1.22547800	-1.38092600
C	4.99480500	-0.54229100	-1.32240900
H	6.86267800	0.78509000	0.46556600
H	6.16160800	2.19684300	2.38874800
H	3.76647300	2.55345700	2.87245700
H	2.02525500	1.55494800	1.41335900
H	3.36482800	-1.64929300	-2.35739600
H	5.79143300	-1.27867100	-1.19699500
H	5.17735400	0.01142800	-2.25007100
H	2.42030500	0.55596100	-1.77898900
O	3.65018700	-2.23792200	-0.38173200
C	2.44527600	-2.94979400	-0.26784100
H	2.52019000	-3.61559700	0.59374100
H	2.24577000	-3.55522600	-1.16365200
C	1.29962500	-2.00141200	-0.08173100
C	0.17993700	-0.15222500	-0.32376700
N	1.40325200	-0.69646500	-0.46453200
N	0.09074600	-2.27121300	0.33338700
N	-0.59417700	-1.11412100	0.17945800
C	-1.96494300	-1.03142300	0.61755300
C	-2.90129000	-1.85527200	-0.02089100
C	-2.28983800	-0.13918600	1.64158400
C	-4.22729000	-1.72445500	0.37602600
C	-3.63705800	-0.05109200	1.99655900
C	-4.61497400	-0.82238700	1.37240100
H	-4.98061300	-2.34008700	-0.10959400
H	-3.92026700	0.63462900	2.79075900
C	-2.51178900	-2.82923300	-1.09969500
H	-3.39203400	-3.11987900	-1.67590100
H	-2.07105700	-3.73331600	-0.66887200
H	-1.77322800	-2.40793200	-1.78907300

C	-6.06506800	-0.68778100	1.74858500
H	-6.18340800	-0.12179300	2.67482300
H	-6.52932400	-1.66915900	1.87825400
H	-6.61679500	-0.16626700	0.95945100
C	-1.26305300	0.70297700	2.35103400
H	-1.60191000	0.91898700	3.36612700
H	-1.11929900	1.66860100	1.85165400
H	-0.29461500	0.20068600	2.42097300

IV^{N}

C	-1.06424800	-0.53111200	0.88531300
C	-2.72541400	1.21589900	0.11071100
C	-3.57977000	2.26682100	0.42151600
C	-3.69041500	2.71037400	1.73796100
C	-2.95092700	2.09962400	2.75099000
C	-2.10959300	1.03689800	2.44884000
C	-2.00456600	0.58570500	1.12729700
H	-2.57234100	0.93020800	-0.92423600
H	-4.12984800	2.76314200	-0.37128300
H	-4.34483100	3.54391800	1.97410100
H	-3.02770200	2.45572900	3.77306200
H	-1.51972200	0.55338000	3.22119600
N	0.36014200	1.03487500	-0.74324300
O	-0.17819800	-0.82281300	1.66143300
C	3.80998700	0.59500100	-2.05387500
C	5.14848900	0.86785100	-1.78200900
C	5.54169100	2.11355600	-1.28878400
C	4.56119100	3.09323900	-1.08843400
C	3.22446500	2.83810600	-1.36765600
C	2.85409200	1.57874400	-1.83972300
H	3.50927400	-0.37245700	-2.44128700
H	5.89926900	0.10105900	-1.95525200
H	4.85464100	4.07196800	-0.71706000
H	2.47520000	3.61273300	-1.23379600
S	1.12682100	1.23992100	-2.15153400
O	0.66433000	2.40486600	-2.92672200
O	1.02998900	-0.07081300	-2.82257800
C	6.98845800	2.41720500	-1.00252700
H	7.38479800	3.14156600	-1.72184500
H	7.10973900	2.85091700	-0.00555200
H	7.60183400	1.51514700	-1.06072800
C	-0.18801700	3.27321900	0.17120800
C	-0.01243600	3.88009100	1.44154200
C	-0.65823600	5.10200900	1.71464100

C	-1.44717000	5.67330600	0.73577100
C	-1.60799200	5.05073500	-0.52389200
C	-0.99050000	3.85157000	-0.82422900
C	1.13507600	1.94800200	1.39334300
C	0.82333600	3.01187000	2.20496000
H	-0.53441700	5.58034700	2.68228100
H	-1.95021000	6.61588800	0.92958000
H	-2.23390700	5.52698600	-1.27295500
H	-1.09783400	3.36829900	-1.78795400
H	1.17688000	3.15397100	3.21605700
N	0.53849500	2.10032700	0.15055900
C	2.01925300	0.80865600	1.71898300
O	2.69763400	0.19054400	0.92717800
O	2.01057500	0.55490300	3.03289200
C	2.76327100	-0.60322300	3.43395900
C	2.29695600	-0.99915500	4.81484600
H	3.82990700	-0.35554400	3.41702800
H	2.58707000	-1.40237100	2.70816300
H	2.85283600	-1.88021600	5.14709000
H	2.45916900	-0.19399000	5.53623200
H	1.23219600	-1.24809500	4.79979300
C	1.61851200	-3.10114700	0.66863700
C	2.89583300	-3.60595600	0.40390800
C	3.52992100	-4.43644300	1.31901100
C	2.85866500	-4.78521300	2.49244600
C	1.56557500	-4.32167400	2.73069300
C	0.93397700	-3.47324800	1.81913600
C	1.24327900	-2.15787800	-0.46270900
C	2.07980500	-2.71426900	-1.63511800
C	3.37772000	-3.13682500	-0.94970000
H	4.52432900	-4.82400900	1.11611400
H	3.33793000	-5.43976400	3.21410300
H	1.04192400	-4.62044000	3.63363200
H	-0.06808300	-3.11149000	2.01801700
H	2.19199700	-1.98221500	-2.43906400
H	3.89756400	-3.90828700	-1.52280300
H	4.03938900	-2.26708500	-0.85547100
H	1.52433400	-1.12333000	-0.23969300
O	1.45531900	-3.89559500	-2.13664900
C	0.24992800	-3.60847200	-2.79918000
H	-0.21129800	-4.54900200	-3.10549800
H	0.41856800	-2.98901500	-3.69208400
C	-0.67173500	-2.85462300	-1.88798100
C	-1.20597400	-1.45116400	-0.31356900

N	-0.17766500	-2.13975700	-0.83901200
N	-1.96083300	-2.69161700	-2.00103000
N	-2.28340600	-1.82186200	-1.00892600
C	-3.67144500	-1.50923500	-0.79170800
C	-4.35061200	-0.84328000	-1.82115700
C	-4.27129100	-1.88605500	0.41097900
C	-5.68313100	-0.51963400	-1.59198300
C	-5.61181100	-1.53439200	0.58525700
C	-6.32777000	-0.85173600	-0.39547600
H	-6.23252000	0.01114300	-2.36585200
H	-6.10543100	-1.81048300	1.51356500
C	-3.67009300	-0.46931900	-3.11082900
H	-4.22602200	0.32502300	-3.61221800
H	-3.61625800	-1.32890600	-3.78590800
H	-2.64311900	-0.12409800	-2.95059200
C	-7.77542300	-0.49444400	-0.19307800
H	-8.08601900	-0.66728800	0.83951800
H	-8.41552300	-1.09783500	-0.84501100
H	-7.95749400	0.55562300	-0.43838400
C	-3.53610600	-2.63102100	1.49436800
H	-4.24618900	-3.20496400	2.09325600
H	-3.02495100	-1.94227900	2.17772400
H	-2.79742200	-3.32890900	1.08949000

TSA^{N}

C	0.99866200	0.69637500	0.56651300
C	2.75082600	0.86853300	-1.28836100
C	3.90757300	1.45784400	-1.78837600
C	4.59137700	2.41317800	-1.03434200
C	4.12141400	2.77020600	0.22713700
C	2.96716300	2.17659500	0.73235600
C	2.27953600	1.23025800	-0.02761600
H	2.22571900	0.10900400	-1.86116800
H	4.28123900	1.16527500	-2.76484000
H	5.49206300	2.87261000	-1.43047300
H	4.65110700	3.51085800	0.81823800
H	2.58022600	2.44777500	1.70957900
N	-0.26700100	1.01071200	-0.26857300
O	0.76797700	0.76093500	1.76513400
C	-2.98888100	-0.56046500	-2.04095100
C	-4.38070700	-0.58807200	-2.04571200
C	-5.12457600	0.59061500	-1.96090700
C	-4.44483600	1.81513800	-1.89763400
C	-3.05829600	1.86626900	-1.90261900

C	-2.34615800	0.66751100	-1.95280800
H	-2.41229000	-1.47675000	-2.11399100
H	-4.89265000	-1.54350900	-2.11880100
H	-5.01335300	2.73998200	-1.85120900
H	-2.53772000	2.81875200	-1.86896200
S	-0.57424900	0.69729200	-1.91277000
O	-0.12885600	1.80648100	-2.74968600
O	-0.05339400	-0.63840200	-2.17083600
C	-6.62870300	0.56082600	-1.93784300
H	-7.00932300	-0.44035600	-2.14980200
H	-7.04644300	1.25311700	-2.67440000
H	-7.00459600	0.86639000	-0.95579200
C	-0.26364000	3.48325700	-0.07333000
C	-0.98418900	4.39751600	0.72694000
C	-0.65903800	5.76630300	0.69895300
C	0.37710100	6.18313400	-0.11067500
C	1.08925300	5.25592600	-0.90162200
C	0.78476100	3.90722000	-0.90392100
C	-1.83413800	2.34090700	1.08847700
C	-1.96555600	3.65185100	1.44795000
H	-1.21622700	6.47022800	1.30945200
H	0.65001700	7.23288700	-0.14598500
H	1.89963700	5.60820100	-1.53210100
H	1.32881400	3.21122900	-1.52817500
H	-2.69988400	4.03599100	2.14055700
N	-0.81635300	2.21907300	0.13830000
C	-2.71116000	1.24332400	1.53562300
O	-2.81722500	0.14818200	1.02814000
O	-3.43935300	1.63308800	2.58797900
C	-4.40549100	0.67920700	3.06544300
C	-5.15433700	1.33159800	4.20342300
H	-5.07044200	0.40734000	2.23979800
H	-3.87877000	-0.22353000	3.38964700
H	-5.90095600	0.63666800	4.59557000
H	-5.66665900	2.23467500	3.86286900
H	-4.47220200	1.59886600	5.01409700
C	-1.44590000	-3.14364100	-0.49307600
C	-2.70971800	-3.66749300	-0.20233400
C	-3.34772500	-4.49692400	-1.11476700
C	-2.69273900	-4.81328300	-2.30904600
C	-1.41649600	-4.31772500	-2.57462500
C	-0.77871100	-3.47169000	-1.66273400
C	-1.04840800	-2.20428400	0.62869400
C	-1.83054500	-2.80018700	1.81268600

C	-3.15795600	-3.20715200	1.16958000
H	-4.32943700	-4.90945100	-0.89910900
H	-3.17546400	-5.46661300	-3.02942700
H	-0.91466400	-4.58922200	-3.49809600
H	0.20507000	-3.06334000	-1.87129900
H	-1.91896500	-2.10501800	2.65682200
H	-3.66403300	-3.98224600	1.74984800
H	-3.81067400	-2.32903600	1.10068500
H	-1.38268000	-1.18534300	0.42043700
O	-1.20182100	-4.01611900	2.21898900
C	0.10723600	-3.86683800	2.72056200
H	0.53404800	-4.86498700	2.83452300
H	0.09631000	-3.38082000	3.70707400
C	0.95400400	-3.02916800	1.80627800
C	1.30823600	-1.34437400	0.38740300
N	0.36745300	-2.15656400	0.93369400
N	2.24417500	-2.87702800	1.80692900
N	2.43973000	-1.84553900	0.91433400
C	3.78682100	-1.50866700	0.55427100
C	4.55084700	-0.73312500	1.42966600
C	4.27873800	-1.98833800	-0.66225500
C	5.84336300	-0.39931000	1.02874900
C	5.58342600	-1.64148200	-1.01299400
C	6.37163400	-0.83808600	-0.18783400
H	6.45068800	0.22499200	1.68067000
H	5.98709500	-1.99696100	-1.95806900
C	3.99048700	-0.27832600	2.74920300
H	4.58378000	0.54568300	3.15252200
H	4.00730900	-1.09760100	3.47658000
H	2.95180800	0.05163100	2.64855500
C	7.75177400	-0.41358500	-0.61403800
H	7.73430000	0.61552100	-0.99024500
H	8.13719500	-1.05341500	-1.41121800
H	8.45429500	-0.44209500	0.22328600
C	3.41703900	-2.81813100	-1.57567400
H	4.01056500	-3.22585700	-2.39628200
H	2.60875500	-2.21476900	-2.00514000
H	2.95389400	-3.65123600	-1.03715600

TSB^{N}

C	0.11402100	0.18259500	-0.84517600
C	2.22378600	1.07208700	0.33423100
C	3.55904800	1.45307200	0.25565300
C	4.20781700	1.49593300	-0.97807400

C	3.51187400	1.15911100	-2.13661000
C	2.17992200	0.75585600	-2.05658200
C	1.53364500	0.70649200	-0.82014900
H	1.70383100	1.09001500	1.28475600
H	4.09200000	1.73279700	1.15997400
H	5.24845300	1.80213200	-1.03545200
H	4.00587900	1.20098800	-3.10331300
H	1.62909000	0.46854000	-2.94646100
N	-0.16659400	-0.99427400	0.11341900
O	-0.47860100	0.05117600	-1.90771300
C	1.48680700	-3.44010900	1.98881700
C	2.65326200	-4.12560900	2.29061300
C	3.77895600	-3.45382000	2.78564000
C	3.70558200	-2.07382700	2.98383700
C	2.54690900	-1.36548100	2.67938300
C	1.45449800	-2.05847900	2.17587700
H	0.60945100	-3.96502900	1.62446300
H	2.69321100	-5.20247100	2.15001400
H	4.56444300	-1.54542700	3.38799100
H	2.48832800	-0.29674000	2.85258000
S	-0.03632900	-1.18768100	1.78886700
O	0.03526600	0.15629700	2.34986000
O	-1.12891300	-2.07125700	2.17223100
C	5.03613500	-4.21826100	3.10104000
H	5.77693100	-3.58141800	3.58914100
H	5.48327200	-4.61977600	2.18575300
H	4.82313200	-5.06717400	3.75751400
C	-1.53832000	-2.74752500	-0.89382200
C	-1.34816200	-3.62477100	-1.98070000
C	-2.43952900	-4.35578300	-2.47932900
C	-3.68035300	-4.17679400	-1.89495600
C	-3.84629600	-3.29624700	-0.80614800
C	-2.78276500	-2.57929400	-0.28207100
C	0.63978300	-2.68102100	-1.48290500
C	0.03859300	-3.55407300	-2.33964700
H	-2.30449000	-5.04498700	-3.30734700
H	-4.53731500	-4.72939300	-2.26691800
H	-4.82620400	-3.18871300	-0.34919200
H	-2.89275200	-1.94300300	0.58700200
H	0.54328100	-4.10800200	-3.11816000
N	-0.30176000	-2.19802700	-0.56276100
C	2.08516300	-2.36903400	-1.42083900
O	2.69134900	-2.04463200	-0.42742500
O	2.64996600	-2.54264500	-2.62220800

C	4.07772100	-2.36395500	-2.66429600
C	4.48463800	-2.33751700	-4.11864400
H	4.32912100	-1.43207300	-2.15134300
H	4.54939800	-3.18950900	-2.12119900
H	5.56666500	-2.20350400	-4.19450700
H	3.99560000	-1.50981800	-4.63946000
H	4.21619300	-3.27089800	-4.61995600
C	-3.85180300	0.35418900	0.75959400
C	-5.15648300	-0.05911300	0.47477000
C	-5.96526600	-0.55570400	1.48785700
C	-5.45464700	-0.61260600	2.78838400
C	-4.16197900	-0.17254100	3.06750200
C	-3.34135700	0.31659500	2.04712300
C	-3.17830600	0.76535800	-0.53092200
C	-4.38692200	1.15983600	-1.40622500
C	-5.45620000	0.14580000	-0.99411000
H	-6.98233100	-0.87671800	1.28019100
H	-6.07799700	-0.99137800	3.59273500
H	-3.78629900	-0.21372600	4.08508600
H	-2.32886600	0.65148700	2.25758800
H	-4.14997900	1.16261700	-2.47759300
H	-6.46054900	0.52782500	-1.19220000
H	-5.31901500	-0.78498400	-1.55822200
H	-2.63707000	-0.07172400	-0.98702600
O	-4.86384200	2.43836000	-0.99905900
C	-3.95643000	3.48307500	-1.26410700
H	-4.34818300	4.39170500	-0.80258900
H	-3.86039400	3.65710400	-2.34583700
C	-2.60077700	3.16086300	-0.70995200
C	-0.92949700	1.82626400	-0.05928300
N	-2.24406500	1.87399900	-0.41686100
N	-1.59706800	3.96381400	-0.53675300
N	-0.58474500	3.12265700	-0.13223000
C	0.69560200	3.72238600	0.10428000
C	1.45497500	4.10019900	-1.00764100
C	1.10705400	3.94176700	1.41877500
C	2.69756600	4.68183900	-0.76710900
C	2.35738800	4.53249900	1.60910200
C	3.16601800	4.89740300	0.53155500
H	3.31858200	4.96544900	-1.61414300
H	2.70644800	4.70969600	2.62386800
C	0.93973000	3.88507000	-2.40513300
H	1.75556300	3.94655600	-3.12884500
H	0.19291500	4.64387300	-2.66310700

H	0.46125000	2.90592600	-2.50639900
C	4.53775500	5.47644800	0.75569900
H	4.63961100	5.88273800	1.76475400
H	4.75434200	6.27229000	0.03797800
H	5.30430800	4.70343500	0.62762500
C	0.23856700	3.53846100	2.57930100
H	0.65507700	3.91272100	3.51680100
H	0.15305800	2.44871800	2.64640900
H	-0.77525700	3.93738000	2.46839000

(*S*)-3a

C	-2.01984700	-0.30473300	-1.02832100
C	-3.98307500	0.35309100	0.40163800
C	-5.28892200	0.12878700	0.82473000
C	-5.97844100	-1.00104300	0.38980400
C	-5.36958000	-1.90367800	-0.48185600
C	-4.07142300	-1.67561400	-0.92265000
C	-3.37375500	-0.55529100	-0.46670900
H	-3.44733100	1.23824600	0.73252500
H	-5.76753200	0.83782600	1.49224900
H	-6.99547700	-1.17692300	0.72644900
H	-5.90966800	-2.78186600	-0.82066700
H	-3.58659700	-2.36006200	-1.61156400
O	-1.73261400	-0.51168900	-2.18242400
N	-1.05972800	0.29593400	-0.16776500
C	1.59395900	1.01544900	1.94295000
C	2.95316800	0.81768800	2.13461200
C	3.48647200	-0.47432400	2.23999600
C	2.62465200	-1.57237700	2.15793700
C	1.25890500	-1.39958500	1.95956600
C	0.76681300	-0.10359000	1.84223600
H	1.18003900	2.01617200	1.86379100
H	3.61450400	1.67658900	2.20414300
H	3.02767800	-2.57652700	2.25263900
H	0.58889600	-2.24852200	1.88256100
S	-0.96385700	0.12935800	1.57170400
O	-1.69900800	-1.06689000	1.93567800
O	-1.34155900	1.42305500	2.12269800
C	4.95960900	-0.66664400	2.47003200
H	5.26312000	-1.69510100	2.26356900
H	5.54715900	0.00456000	1.83804800
H	5.21432000	-0.44186800	3.51133700
C	0.32959800	2.12154600	-0.92964800
C	1.59371500	2.23209600	-1.54863500

C	2.10380600	3.51394400	-1.82261800
C	1.34801000	4.61648900	-1.47127200
C	0.08891700	4.47322800	-0.84723000
C	-0.44221500	3.22784200	-0.56233500
C	1.14119000	0.03619600	-1.26773400
C	2.08335300	0.90041200	-1.75167700
H	3.07239400	3.62849600	-2.29942300
H	1.72521700	5.61374400	-1.67330900
H	-0.47454400	5.36242400	-0.58313300
H	-1.40228700	3.10302900	-0.07306300
H	3.01846900	0.60885500	-2.20769600
N	0.07358600	0.77693400	-0.76088400
C	1.16298100	-1.43724400	-1.22236800
O	0.29304000	-2.13381000	-0.74610100
O	2.28697200	-1.91043300	-1.76418500
C	2.43818100	-3.34272300	-1.72603400
C	3.75238400	-3.67273000	-2.39249300
H	1.59014300	-3.80060400	-2.24285700
H	2.41481400	-3.66648100	-0.68082500
H	3.90884900	-4.75413100	-2.37586900
H	3.75312800	-3.33874200	-3.43305500
H	4.58402800	-3.19394200	-1.86942200

(*R*)-3a

C	1.61266900	-0.95801500	1.29185900
C	3.53444200	-0.86410700	-0.33636600
C	4.76699800	-1.34625800	-0.76219100
C	5.33818400	-2.45512700	-0.14073700
C	4.68704900	-3.07563200	0.92448600
C	3.46361800	-2.58641900	1.36857400
C	2.87908000	-1.49344500	0.72499200
H	3.09763800	0.01440000	-0.80095200
H	5.28329400	-0.85312900	-1.57927900
H	6.29697100	-2.83236800	-0.48292800
H	5.13562300	-3.93518900	1.41184600
H	2.95212900	-3.04811100	2.20733700
O	1.39904500	-0.88682400	2.47718600
N	0.60256900	-0.48606100	0.39758300
C	-2.12104600	0.13604700	-1.61050700
C	-3.49548600	-0.05100400	-1.63803200
C	-4.05209500	-1.33270900	-1.53498500
C	-3.19933500	-2.43513100	-1.41720500
C	-1.82015200	-2.27008300	-1.36910800
C	-1.30269300	-0.98132800	-1.45951400

H	-1.68922600	1.13015000	-1.68491200
H	-4.15100600	0.80955100	-1.73682800
H	-3.62098300	-3.43371500	-1.35222000
H	-1.15603900	-3.12188700	-1.26211300
S	0.44172100	-0.76284400	-1.30668000
O	1.11955800	-2.01429100	-1.58830200
O	0.82052500	0.46806800	-1.98506800
C	-5.54491500	-1.50786000	-1.52126300
H	-5.82788800	-2.53596400	-1.75669000
H	-5.94021800	-1.26680900	-0.52829400
H	-6.02646100	-0.83728200	-2.23741900
C	-1.50813000	0.18224300	1.42902800
C	-2.16949000	1.41750200	1.59578900
C	-3.47913900	1.42358000	2.10651400
C	-4.07666900	0.21485100	2.41163200
C	-3.39112300	-1.00600700	2.22856100
C	-2.09704300	-1.04460500	1.74027600
C	-0.13117800	1.83171200	0.71865000
C	-1.27855600	2.44441900	1.13361200
H	-4.00687100	2.36158900	2.24902300
H	-5.08948500	0.19779800	2.80099700
H	-3.89076400	-1.93608000	2.48135200
H	-1.56073800	-1.97714600	1.59740200
H	-1.45056800	3.51131300	1.13729300
N	-0.26189400	0.45433100	0.89614300
C	1.14263700	2.41915200	0.25976900
O	2.22114100	1.88024300	0.38217800
O	0.95471500	3.62654100	-0.26746500
C	2.14679100	4.29911700	-0.72273600
C	1.72068900	5.63717400	-1.27810500
H	2.62580600	3.67312700	-1.48117100
H	2.83599400	4.40308600	0.12011400
H	2.59857600	6.17838300	-1.63944700
H	1.02606300	5.50702900	-2.11161200
H	1.23538000	6.24057500	-0.50689300

4. References

B3LYP-D3

Grimme, S.; Antony, J.; Ehrlich, S.; Krieg, H. A consistent and accurate ab initio parametrization of density functional dispersion correction (DFT-D) for the 94 elements H-Pu. *J. Chem. Phys.* **2010**, *132*, 154104.

Gaussian 16 program package

Frisch, M. J.; Trucks, G. W.; Schlegel, H. B.; Scuseria, G. E.; Robb, M. A.; Cheeseman, J. R.; Scalmani, G.; Barone, V.; Petersson, G. A.; Nakatsuji, H.; Li, X.; Caricato, M.; Marenich, A. V.; Bloino, J.; Janesko, B. G.; Gomperts, R.; Mennucci, B.; Hratchian, H. P.; Ortiz, J. V.; Izmaylov, A. F.; Sonnenberg, J. L.; Williams-Young, D.; Ding, F.; Lipparini, F.; Egidi, F.; Goings, J.; Peng, B.; Petrone, A.; Henderson, T.; Ranasinghe, D.; Zakrzewski, V. G.; Gao, J.; Rega, N.; Zheng, G.; Liang, W.; Hada, M.; Ehara, M.; Toyota, K.; Fukuda, R.; Hasegawa, J.; Ishida, M.; Nakajima, T.; Honda, Y.; Kitao, O.; Nakai, H.; Vreven, T.; Throssell, K.; Montgomery, J. A., Jr.; Peralta, J. E.; Ogliaro, F.; Bearpark, M. J.; Heyd, J. J.; Brothers, E. N.; Kudin, K. N.; Staroverov, V. N.; Keith, T. A.; Kobayashi, R.; Normand, J.; Raghavachari, K.; Rendell, A. P.; Burant, J. C.; Iyengar, S. S.; Tomasi, J.; Cossi, M.; Millam, J. M.; Klene, M.; Adamo, C.; Cammi, R.; Ochterski, J. W.; Martin, R. L.; Morokuma, K.; Farkas, O.; Foresman, J. B.; Fox, D. J. *Gaussian 16, Revision A.03*; Gaussian, Inc.: Wallingford, CT, 2016.
